# Supplementary material for: Polysorbates’ effects on molecular and thermodynamic properties of phosphorodiamidate morpholino oligonucleotides’ structures
Source: Mol Ther Nucleic Acids. 2026 Jan 24;37(1):102845. doi: 10.1016/j.omtn.2026.102845 (PMC12914542; doi:10.1016/j.omtn.2026.102845)
Supplement: Document S1. Figures S1–S7 and Tables S1–S6 [file mmc1.pdf]

OMTN, Volume *37*

Supplemental information

Polysorbates' effects on molecular and thermodynamic properties of phosphorodiamidate morpholino oligonucleotides' structures

Evgenii Kliuchnikov, Daniel Pierson, Ying Chou, Willow DiLuzio, Kenneth A. Marx, Arani Chanda, and Valeri Barsegov

Supplemental Methods

Force field development for all-atom simulations of PMO with surfactant molecules: In our previous study [1], we derived the atomic partial charges and force-field parameters for the following three parts of the PMO structure: i) for the phosphorodiamidate group attached to the 5'-end and capping the HN atomic group attached to the N3' atom (mimicking the PMO 5'-end); ii) for the phosphorodiamidate group attached to the 3'-end and capping the HO atomic group attached to the O5' atom (mimicking the PMO 3'-end); and iii) for the phosphorodiamidate group attached to both 5'- and 3'-ends. In the same study [1], we also derived the force field parameters and partial charges for the morpholino triethylene glycol (TEG) piperazine linker attached to the 5'-end of PMO through the phosphorodiamidate group. For both surfactants Polysorbate 20 and Polysorbate 80, we first reconstructed the initial structure based on the 2D-schemes (Fig 1C in the main text) in GaussView 5.0 [2]. Next, we performed energy minimization and optimization of the structures, using Hartree-Fock (HF) theory with the 6-31G* basis set using the Gaussian 16 package [3]. In the derivation of partial charges for the PMO structure carried out in our previous study [1], as well as for the structures of Polysorbate 20 and Polysorbate 80, we employed the Restrained Electro Static Potential (PESP) charge fitting procedure [4], implemented in the RED server (RESP ESP charge Derive Server) [5]. We utilized the RESP method in conjunction with HF theory and the 6-31G* basis set. Several *ab initio* calculations were carried out for each fragment, including the morpholino ring (5'-, 3'-, and central) with guanine, cytosine, thymine, and adenine bases, with the TEG piperazine linker, and for the entire Polysorbate 20 and Polysorbate 80 molecules. For each fragment, we carried out 3 independent runs (total of 39 runs) over which the final partial charges were averaged. The Antechamber package [6] was employed to assign the all-atom types for atoms in these fragments. Molecular mechanical parameters were obtained from the bsc0$_{\chi OL3}$ force field for nucleic acids and from the ff14SB force field for proteins, for these atom types. These include the equilibrium bond distances (r_0), spring constants for covalent bonds (k_b), equilibrium bond angles (θ_0), and spring constants for bending of bond angles (k_θ), as well as torsional angle parameters (magnitude associated with torsion energy ($V_n/2$), phase offset (γ) and periodicity (n)). For the atom types not covered in the bsc0$_{\chi OL3}$ and ff14SB force fields, the molecular mechanical parameters were generated using the general Amber force field GAFF [7]. For model details of the force field development procedure, including the molecular mechanics parameters and partial charges, the reader should consult our previous work [1] (see also the SI in Ref. [1]). For each PMO molecule and for each surfactant molecule, we created the topology files for each of the "morpholino nucleotides", for the TEG piperazine linker, and for the surfactants. Next, we linked "morpholino nucleotides" together through the phosphorodiamidate groups and added the TEG piperazine linker at the 5'-end (see Fig. 1 in the main text). These structures were then used in all the MD simulations described in the main text part.

Analysis of MD simulation output: The results of MD simulations for PMO-surfactant complexes (coordinate and energy files) were used in data analysis and visualization. *The radius of gyration* R_g was calculated using the coordinates of all atoms,

$$R_g = (\sum_p m_p \boldsymbol{r}_p^2 / \sum_p m_p)^{1/2} \qquad (Equation\ S1)$$

where m_p is the mass and $\boldsymbol{r}_p$ is the position of atom p, relative to the center of mass of the molecule. *Base stacking and base pairing:* If three conditions are satisfied:

$$|z_{kj}|\ and\ |z_{jk}| > 2Å \qquad (Equation\ S2)$$

and

$$\rho_{kj}\ or\ \rho_{jk} < 2.5Å \qquad (Equation\ S3)$$

and

$$\left|\theta_{kj}\right| < 40° \qquad (Equation\ S4)$$

bases were categorized as stacked. Here,

$$\rho_{ij} = \sqrt{x_{kj}^2 + y_{kj}^2} \qquad (Equations\ S5)$$

where the x- and y-axes lie in the plane of the base (x_{kj} and y_{kj} are the distances between the centers of mass of the two bases along the x- and y-axes, respectively) and the z-axis is perpendicular to the xy-plane, z_{kj} is the distance between the centers of mass of the two bases, and θ_{kj} is the angle between the normal vectors of the two bases (see Fig. S1 in Ref. [8]). All the non-stacked bases are considered to be base-paired if $\left|\theta_{kj}\right| < 60°$ and there is at least one hydrogen bond (H-bond) between k-th and j-th bases (see Fig. S1 in Ref. [8]). The total number of base pairs and number of base stackings were calculated using Barnaba software [8] with the structure schematic shown in Fig. S1 in Ref. [1] guiding the calculation. *The Solvent Accessible Surface Area* (SASA) was estimated using the LCPO algorithm [9] implemented in the CPPTRAJ module [10] in AmberTools20 [11]. For RMSD analysis, all frames in the simulation trajectories were superimposed onto the initial energy-minimized structure of the PMO or the PMO–surfactant complex after removing all water molecules. The RMSD was then calculated using the atom selection keyword "nucleic" in VMD, which includes atoms from both the backbone and the nucleobases of the nucleic acid. We used the formula

$$RMSD = \sqrt{\frac{1}{N}\sum_{i=1}^{N} {\delta_i}^2} \qquad (Equation\ S6)$$

where δ_i is the distance between atom i in the current frame and its corresponding atom in the reference structure at t = 0, and N is the number of atoms in the nucleic acid selection. *PMO-PMO and PMO-surfactant interactions:* We assume that a pair of atomic groups (in modified nucleotides of PMOs and surfactant tails in Polysorbate 80 and Polysorbate 20) forms a contact if the distance between the center of mass of the modified nucleotide and any C or O atom in surfactant' tails $d_{RES} <$ 7.5Å-cutoff. The statistics of the distances was obtained with the MDAnalysis Python package [12,13]. *Hydrogen bonds:* We assume that the H-bond D–H…A between the hydrogen donor atom (D) and acceptor atom (A) is formed if the donor–acceptor distance $d_{DA} <$ 3.3Å-cutoff and if the bond angle is larger than the 140°-cutoff [8]. To find the time fraction for each interaction, we used the H-bond option in the CPPTRAJ module [14] implemented in AmberTools20 [11].

Classification of Polysorbate 80 conformations: Based on the numerical output from the all-atom MD simulations of Polysorbate 80, a total of 500 conformations (structure snapshots) of Polysorbate 80 structures were selected for the statistical modeling and classification. For each of these 500 conformations, we calculated the corresponding values of R_g, $SASA$, $RMSD$, L and W described in the main text. The reference structures for calculation of RMSD values for Polysorbate 80 were set to the initial structure of Polysorbate 80 (at time t = 0). By combining these values together, we constructed a dataset containing the values of R_g, $SASA$, $RMSD$, L and W for structure classification. We employed the Support Vector Machines (SVM) approach to perform data classification of Polysorbate 80 molecular conformations into the "collapsed conformations" and the "extended conformations". "Classify method" option implemented in Mathematica with radial basis function (RBF) kernel was used as a numerical implementation of the SVM algorithm [15]. The centroids of each class (central structures) for the extended and collapsed conformation class were constructed as the average collapsed conformation and the average extended conformation. The results obtained are displayed in Fig. 4B in the main text.

Supplemental references:

1. Maksudov, F., Kliuchnikov, E., Pierson, D., Ujwal, M.L., Marx, K.A., Chanda, A., and Barsegov, V. (2023). Therapeutic phosphorodiamidate morpholino oligonucleotides: Physical properties, solution structures, and folding thermodynamics. Molecular Therapy-Nucleic Acids *31*, 631–647.
2. Dennington, R., Keith, T., Millam, J., and others (2009). GaussView, version 5.
3. Frisch, M.J., Trucks, G.W., Schlegel, H.B., Scuseria, G.E., Robb, M.A., Cheeseman, J.R., Scalmani, G., Barone, V., Petersson, G.A., Nakatsuji, H., et al. (2016). Gaussian 16. Preprint at Gaussian, Inc. Wallingford, CT.
4. Cieplak, P., Cornell, W.D., Bayly, C., and Kollman, P.A. (1995). Application of the multimolecule and multiconformational RESP methodology to biopolymers: Charge derivation for DNA, RNA, and proteins. J Comput Chem *16*, 1357–1377.
5. Vanquelef, E., Simon, S., Marquant, G., Garcia, E., Klimerak, G., Delepine, J.C., Cieplak, P., and Dupradeau, F.-Y. (2011). RED Server: a web service for deriving RESP and ESP charges and building force field libraries for new molecules and molecular fragments. Nucleic Acids Res *39*, W511–W517.
6. Wang, J., Wang, W., Kollman, P.A., and Case, D.A. (2006). Automatic atom type and bond type perception in molecular mechanical calculations. J Mol Graph Model *25*, 247–260.
7. Wang, J., Wolf, R.M., Caldwell, J.W., Kollman, P.A., and Case, D.A. (2004). Development and testing of a general amber force field. J Comput Chem *25*, 1157–1174.
8. Bottaro, S., Bussi, G., Pinamonti, G., Reißer, S., Boomsma, W., and Lindorff-Larsen, K. (2019). Barnaba: software for analysis of nucleic acid structures and trajectories. Rna *25*, 219–231.
9. Weiser, J., Shenkin, P.S., and Still, W.C. (1999). Approximate atomic surfaces from linear combinations of pairwise overlaps (LCPO). J Comput Chem *20*, 217–230.
10. Roe, D.R., and Cheatham III, T.E. (2013). PTRAJ and CPPTRAJ: software for processing and analysis of molecular dynamics trajectory data. J Chem Theory Comput *9*, 3084–3095.
11. Case, D.A., Belfon, K., Ben-Shalom, I., Brozell, S.R., Cerutti, D., Cheatham, T., Cruzeiro, V.W.D., Darden, T., Duke, R.E., Giambasu, G., et al. (2020). Amber 2020. Preprint.
12. Michaud-Agrawal, N., Denning, E.J., Woolf, T.B., and Beckstein, O. (2011). MDAnalysis: a toolkit for the analysis of molecular dynamics simulations. J Comput Chem *32*, 2319–2327.
13. Gowers, R.J., Linke, M., Barnoud, J., Reddy, T.J.E., Melo, M.N., Seyler, S.L., Domanski, J., Dotson, D.L., Buchoux, S., Kenney, I.M., et al. (2016). MDAnalysis: a Python package for the rapid analysis of molecular dynamics simulations. In Proceedings of the 15th python in science conference, p. 105.
14. Roe, D.R., and Cheatham III, T.E. (2013). PTRAJ and CPPTRAJ: software for processing and analysis of molecular dynamics trajectory data. J Chem Theory Comput *9*, 3084–3095.
15. Suchok, S. (2015). Mathematica Data Analysis (Packt Publishing Ltd).

Supplemental Movies

Movie S1. 25-mer conformer III interacting with Polysorbate 80: The movie shows the molecular interactions between the 25-mer PMO and Polysorbate 80 as observed in a 1-μs MD simulation at $T = 300$ K. The MD simulation run was carried out in explicit water (cyan transparent spheres). The PMO molecule is shown in the Twister representation for the backbone (blue line) and paper chains for the nucleobases (red and green). Polysorbate 80 is shown in the Licorice representation (sticks). The length of the movie is 50 s (the movie is played 5×10^7 times slower than the computational experiment).

Movie S2. 30-mer conformer III interacting with Polysorbate 80: The movie shows the molecular interactions between the 30-mer PMO and Polysorbate 80 as observed in a 2.75-μs MD simulation at $T = 300$ K. The MD simulation run was carried out in explicit water (cyan transparent spheres). The PMO molecule is shown in the Twister representation for the backbone (blue line) and paper chains for the nucleobases (red and green). Polysorbate 80 is shown in the Licorice representation (sticks). The length of the movie is 138 s (the movie is played 5×10^7 times slower than the computational experiment).

Movie S3. Polysorbate 80 equilibrium dynamics: The movie shows the conformational fluctuations of Polysorbate 80 between the extended and collapsed conformations observed in a 1-μs MD simulation run at $T = 300$ K. The MD simulation run was carried out in explicit water (cyan transparent spheres). Polysorbate 80 is shown in the Licorice representation (sticks). The length of the movie is 39 s (the movie is played 3.9×10^7 times slower than the computational experiment).

Movie S4. 30-mer conformer I interacting with Polysorbate 80: The movie shows the molecular interactions between 30-mer conformer I PMO and Polysorbate 80 as observed in a 1-μs MD simulation at T = 300K. The MD simulation run was carried out in explicit water (cyan transparent spheres). The PMO molecule is shown in the Twister representation for the backbone (blue line) and paper chains for the nucleobases (red and green). Polysorbate 80 is shown in the Licorice representation (sticks). The length of the movie is 106 s (the movie is played 1.1×10^8 times slower than the computational experiment).

Supplemental Tables

Table S1. CAC, CMC and PMO: Surfactant interaction stoichiometry (*n*:1*)* analysis from surface tension data: Shown for the 22-mer, 25-mer, and 30-mer PMO solutions with Polysorbate 80 and Polysorbate 20 surfactants are the critical aggregation concentration (CAC), the critical micelle concentration (CMC), and the relative number of surfactant molecules per one PMO molecule *n* at CMC The CMC values are also given for solutions of both Polysorbate 80 and Polysorbate 20 surfactants in the absence of PMOs (molecular weights of Polysorbate 80 and Polysorbate 20 surfactants are 1310 amu and 1227.5 amu, respectively).

	Polysorbate 80			Polysorbate 20		
	CAC (mg/mL)	CMC (mg/mL)	*n*	CAC (mg/mL)	CMC (mg/mL)	*n*
No PMO	NA	0.016	NA	NA	0.068	NA
30-mer	0.0039	3.68	1.74	0.0094	2.10	2.86
25-mer	0.0035	3.93	1.93	0.0084	2.53	2.80
22-mer	0.0022	5.18	1.67	0.0059	4.22	1.92

Table S2. Molecular Mechanical parameters for covalent bonds in surfactants Polysorbate 20 and Polysorbate 80: Shown for each covalent bond are the equilibrium covalent bond distance r_0 and spring constant k_b. These force field parameters were determined for the surfactant molecules explored in this study (see Material and Methods in the main text). Atom types: OH - sp^3 hybridized oxygen in hydroxyl group; HO – hydrogen on oxygen; CT – sp^3 hybridized carbon with 4 explicit substituents; H1 – hydrogen on aliphatic carbon with 1 electron-withdrawing group; HC – hydrogen on aliphatic carbon; OS – ester oxygen; C - sp^2 hybridized carbon in C=O and C=S; O - sp^2 hybridized oxygen in C=O and COO^-; C2 – aliphatic sp^2 hybridized carbon.

Bond	k_b, kcal/(mol·Å^2)	r_0, Å
OH-HO	371.4	0.973
OH-CT	316.7	1.423
CT-CT	300.9	1.537
CT-H1	330.6	1.097
CT-HC	330.6	1.097
CT-OS	308.6	1.432
OS-C	390.8	1.358
C-O	637.7	1.218
C-CT	313.0	1.524
CT-C2	326.8	1.509
C2-C2	569.4	1.334
C2-HC	344.3	1.087

Table S3. Molecular Mechanical parameters for bond angles in surfactants: Shown for each bond angle are the equilibrium bond angle θ_0 and bending angle spring constant k_a.

Angle	k_a, kcal/(mol·rad^2)	θ_0,deg
HO-OH-CT	47.4	107.26
OH-CT-CT	67.5	110.19
OH-CT-HC	51.1	109.50
HC-CT-HC	39.4	107.58
CT-CT-HC	46.3	109.80
CT-CT-OS	68.0	107.97
OS-CT-HC	51.0	108.70
CT-OS-CT	62.7	112.48
CT-CT-CT	62.9	111.51
CT-OS-C	63.3	115.98
OS-C-O	75.3	123.25
O-C-CT	67.4	123.20
C-CT-HC	46.9	108.77
C-CT-CT	63.3	111.04
CT-C2-C2	64.1	123.63
C2-CT-HC	47.0	110.36
CT-C2-HC	45.1	120.00
C2-C2-HC	50.0	119.70

Table S4 Molecular Mechanical parameters for torsion angles in surfactant molecules: Shown for each torsion angle are the number of bond paths and the magnitude of torsion energy $V_n/2$, the phase offset γ, and the periodicity of torsion n. Atom X denotes any atom of the atom type described in the caption to Table S1.

Torsion angle	no. of paths	$V_n/2$, kcal/mol	γ, deg	n
HO-OH-CT-HC	3	0.500	0	3
HO-OH-CT-CT	1	0.250	0	1
X-CT-CT-X	1	0.250	0	1
OH-CT-CT-OS	1	1.175	0	2
CT-CT-OS-CT	1	0.100	180	2
X-OS-CT-X	3	1.150	0	3
OS-CT-CT-OS	1	1.175	0	2
HC-CT-CT-HC	1	0.150	0	3
X-CT-CT-X	9	1.400	0	3
CT-CT-CT-CT	1	0.200	180	1
CT-CT-CT-HC	1	0.160	0	3
CT-CT-OS-C	1	0.800	180	1
CT-OS-C-O	1	1.400	180	1
X-C-CT-X	6	0.000	180	2
O-C-CT-HC	1	0.080	180	3
X-CT-C2-X	6	0.000	0	2
CT-C2-C2-CT	1	1.900	180	1
CT-C2-C2-HC	4	26.600	180	2

Table S5. Dynamic properties of surfactant molecules obtained from MD simulations of PMO-surfactant complexes: Shown for each PMO-surfactant combination are the properties of surfactants: radius of gyration R_g, number of hydrogen bonds formed n_{hb}, solvent accessible surface area ($SASA$), root-mean-square deviation $RMSD$, length L, and width W (averages and standard deviations).

Surfactant	PMO	R_g, nm	n_{hb}	$SASA$, Å^2	$RMSD$, nm	L, nm	W, nm
Polysorbate 80	w/o PMO	0.77±0.08	0.10±0.06	1,757±177	1.22±0.15	1.49±0.61	1.58±0.56
Polysorbate 20	w/o PMO	0.77±0.08	0.10±0.07	1,696±171	1.01±0.12	1.48±0.62	1.56±0.55
Polysorbate 80	25mer I	0.88±0.11	0.07±0.05	1,282±219	2.70±0.60	1.78±0.69	1.75±0.53
Polysorbate 20	25mer I	0.84±0.10	0.08±0.07	1,185±209	2.53±0.32	1.76±0.76	1.57±0.56
Polysorbate 80	25mer II	0.92±0.11	0.07±0.06	1,221±168	2.21±0.40	1.98±0.66	1.79±0.61
Polysorbate 20	25mer II	0.85±0.09	0.06±0.05	1,156±262	2.60±0.49	1.79±0.67	1.75±0.56
Polysorbate 80	25mer III	0.86±0.09	0.08±0.05	1,234±184	1.92±0.52	1.68±0.64	1.70±0.52
Polysorbate 20	25mer III	0.87±0.08	0.07±0.05	1,180±172	2.05±0.41	1.92±0.62	1.69±0.54
Polysorbate 80	30mer I	0.87±0.10	0.06±0.05	1,161±208	2.49±0.58	1.71±0.64	1.73±0.53
Polysorbate 20	30mer I	0.86±0.10	0.09±0.07	1,087±160	2.44±0.50	1.94±0.77	1.69±0.56
Polysorbate 80	30mer II	0.88±0.11	0.07±0.06	1,243±202	2.62±0.69	1.74±0.66	1.74±0.56
Polysorbate 20	30mer II	0.87±0.10	0.07±0.05	1,161±220	2.09±0.44	2.14±0.70	1.71±0.54
Polysorbate 80	30mer III	0.86±0.10	0.09±0.06	1,093±250	2.49±0.42	1.82±0.65	1.65±0.57
Polysorbate 20	30mer III	0.89±0.09	0.07±0.05	1,077±241	2.37±0.38	2.00±0.81	1.63±0.54

Table S6. Interaction energies stabilizing the PMO-surfactant complexes: Shown are the components of the interaction energy (averages and standard deviations) between 25-mer and 30-mer PMO principal solution conformers I–III and surfactants Polysorbate 20 and Polysorbate 80: the van der Waals interaction energy ΔE_{vdW}; the electrostatic interaction energy ΔE_{el}, the solvation free energy ΔE_{solv}, and the total interaction energy ΔE.

PMO	Surfactant	ΔE_{vdW}, kcal/mol	ΔE_{el}, kcal/mol	ΔE_{solv}, kcal/mol	ΔE, kcal/mol
25mer I	Polysorbate 80	-73±24	-27±13	45±15	-55±21
25mer I	Polysorbate 20	-66±23	-23±14	41±15	-53±21
25mer II	Polysorbate 80	-81±21	-25±13	45±14	-64±17
25mer II	Polysorbate 20	-73±26	-25±14	43±16	-59±24
25mer III	Polysorbate 80	-76±23	-26±13	42±14	-64±21
25mer III	Polysorbate 20	-74±21	-28±13	45±14	-63±17
30mer I	Polysorbate 80	-85±26	-29±15	50±16	-64±23
30mer I	Polysorbate 20	-77±21	-25±12	44±13	-65±14
30mer II	Polysorbate 80	-76±20	-22±11	42±12	-60±16
30mer II	Polysorbate 20	-70±22	-21±11	40±13	-54±15
30mer III	Polysorbate 80	-86±25	-26±13	50±15	-62±18
30mer III	Polysorbate 20	-86±25	-31±13	54±17	-67±21

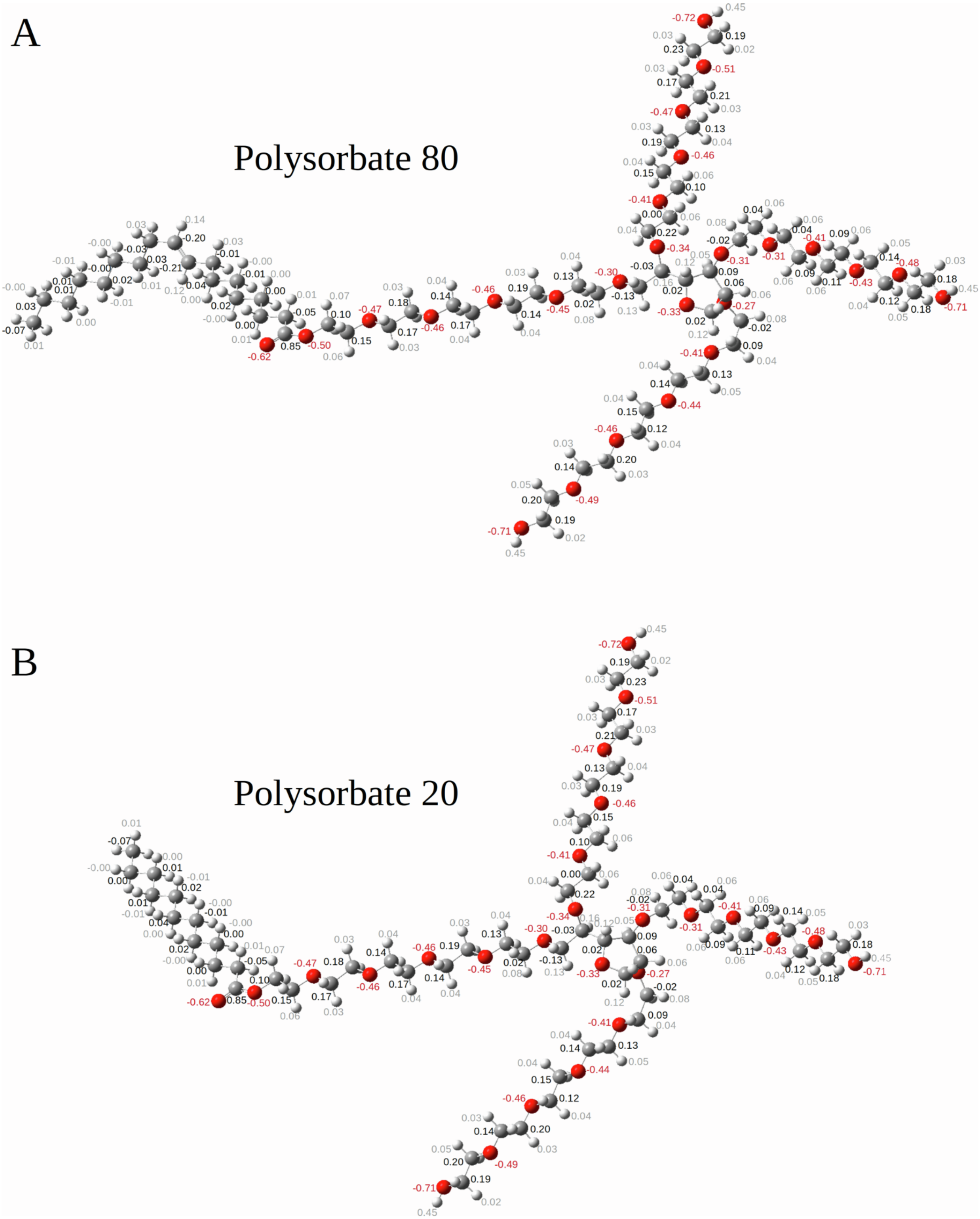

Figure S1. Atomic partial charges for surfactants Polysorbate 80 and Polysorbate 20. Panel **A**: Partial charges for Polysorbate 80. Panel **B**: Partial charges for Polysorbate 20. All atomic partial charges were calculated using the RESP method (see SI Methods).

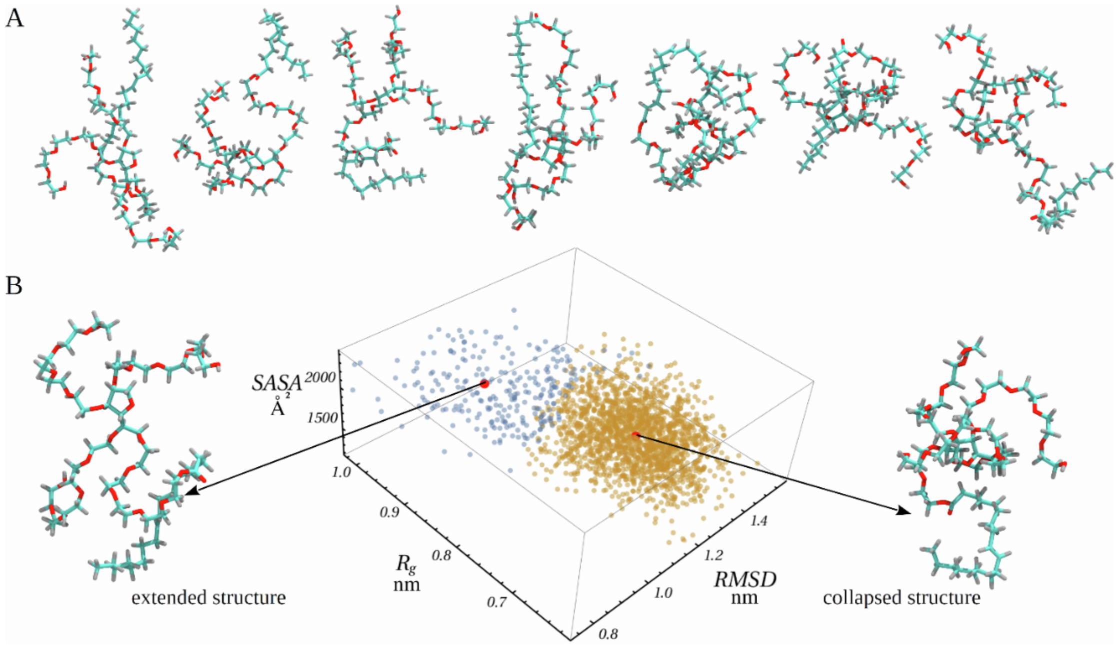

Figure S2. Polysorbate 80's conformations from 1-μs all-atom MD simulations. Seven randomly chosen conformations of Polysorbate 80 show that this surfactant is not able to form a stable structure (panel **A**). These structures were extracted from the set of a total of 500 structures of Polysorbate 80 generated in the 1-μs equilibrium MD simulation run. All 500 structures are shown in a form of 3D scatterplot of R_g vs. $RMSD$ and vs. $SASA$ (panel **B**). The Support Vector Classifier was used to separate the conformations that belong to the 'collapsed' class (yellow dots) and the 'extended' class of conformers (blue dots) as defined in SI. For each cluster, the centroids (red dots) were selected to be shown as the most representative structures.

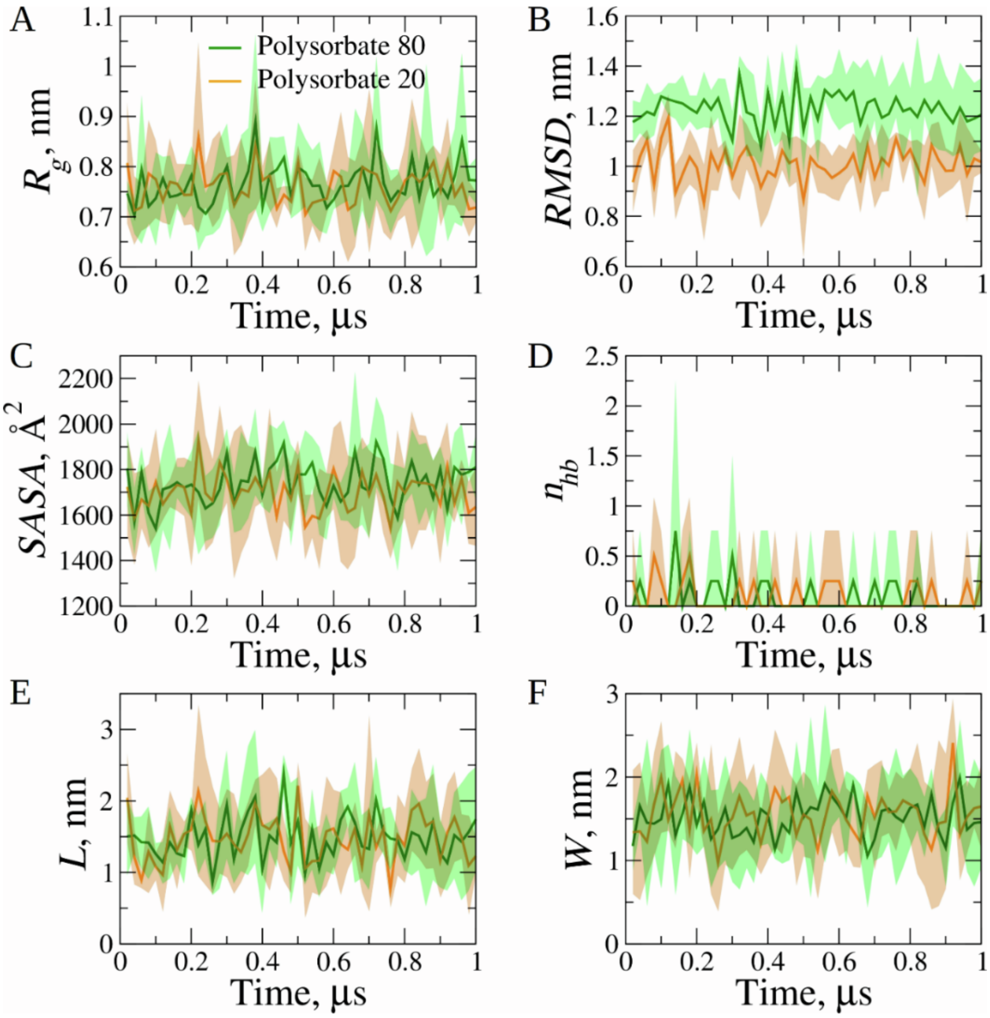

Figure S3. Dynamic structural properties of the surfactants Polysorbate 20 and Polysorbate 80. Time profiles of the radius of gyration R_g (panel **A**), $RMSD$ (panel **B**), $SASA$ (panel **C**), number of hydrogen bonds n_{hb} (panel **D**), length L (panel **E**), and width W (panel **F**). Shown are the averages and standard deviations for Polysorbate 80 (in green color) and Polysorbate 20 (in orange) from 1-μs long MD simulation runs for Polysorbate 20 and Polysorbate 80 (without PMO molecules).

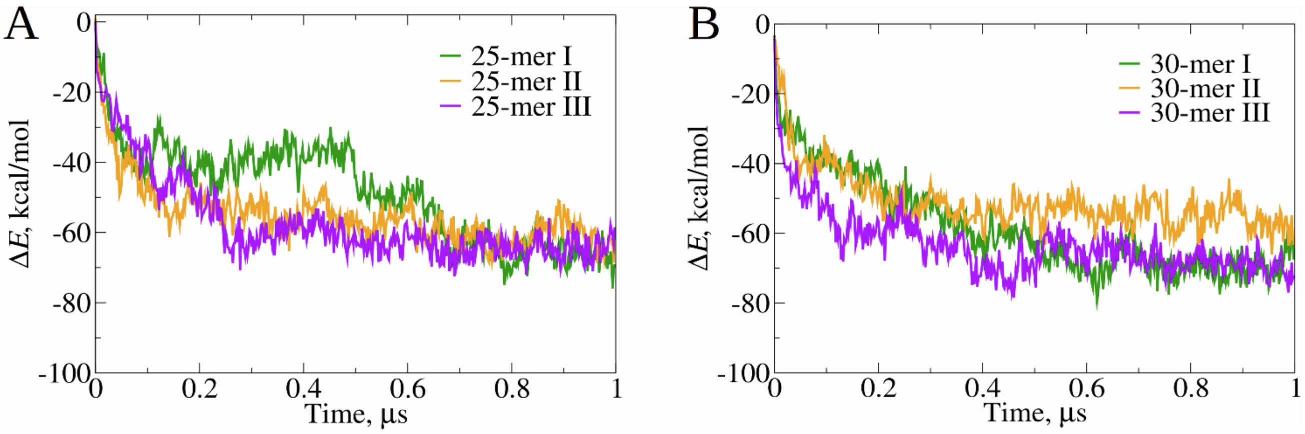

Figure S4. PMO-Polysorbate 20 interaction energies for 25-mer and 30-mer PMOs. Displayed are the time profiles of the total interaction energy ΔE between Polysorbate 20 and the 25-mer PMO conformer I (green), conformer II (orange) and conformer III (purple; panel **A**), and the 30-mer PMO conformer I (green), conformer II (orange) and conformer III (purple; panel **B**). Shown are the averages obtained from 10 all-atom MD simulation runs (see Materials and Methods in the main text). The PMO-Polysorbate 80 interaction energies for the 25-mer and 30-mer PMOs are shown in Fig. 5 in the main text.

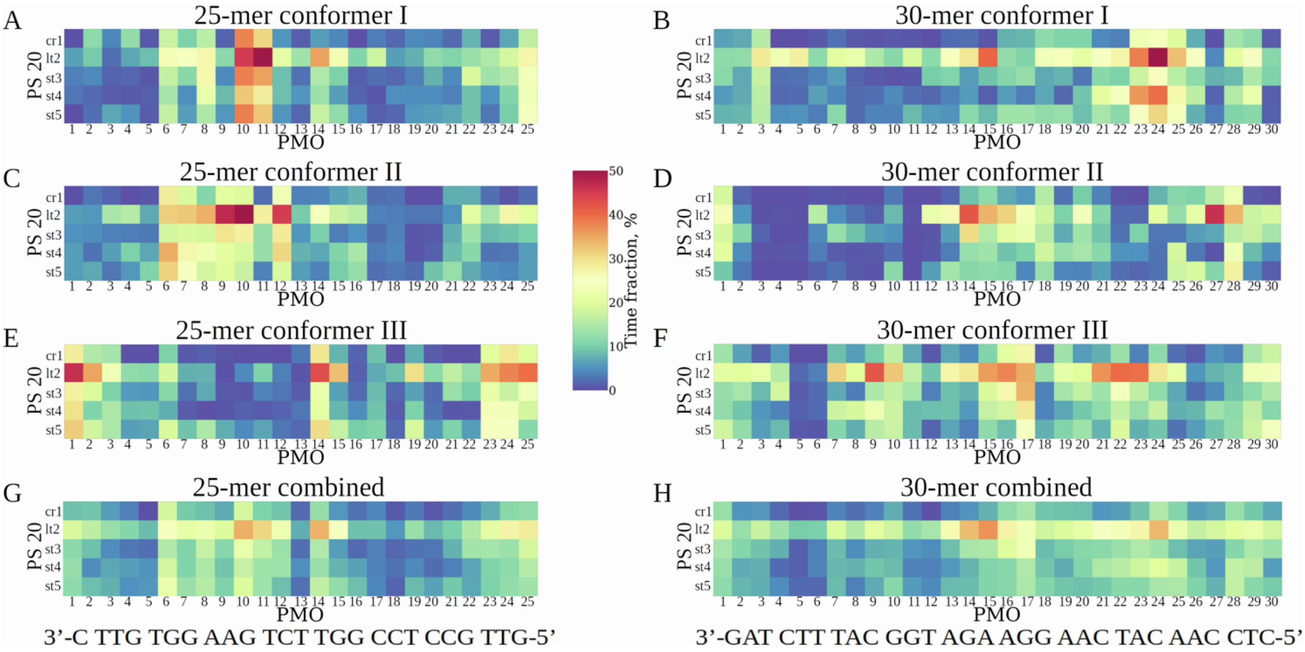

Figure S5. PMO-Polysorbate 20 interactions. Displayed are maps showing the time fraction (percentage of total time) of persistent interactions between PMO bases and surfactant side chains for the 25-mer PMO conformer I (panel **A**), conformer II (panel **C**) and conformer III (panel **E**) as well as the for the 30-mer PMO conformer I (panel **B**), conformer II (panel **D**) and conformer III (panel **F**). Also shown are the interactions maps for all three conformers of 25-mer (panel **G**) and 30-mer (panel **H**) combined. The nomenclature for PMO bases starts from the 3'-end of the PMO, and for the surfactant, cr1 is the central ring (see Fig. 1D), lt2 is the long hydrophobic tail (see Fig. 1D), and st3, st4 and st5 are the short tails (see Fig. 1D). The PMO-Polysorbate 80 interaction maps are shown in Fig. 6 in the main text. PS = Polysorbate.

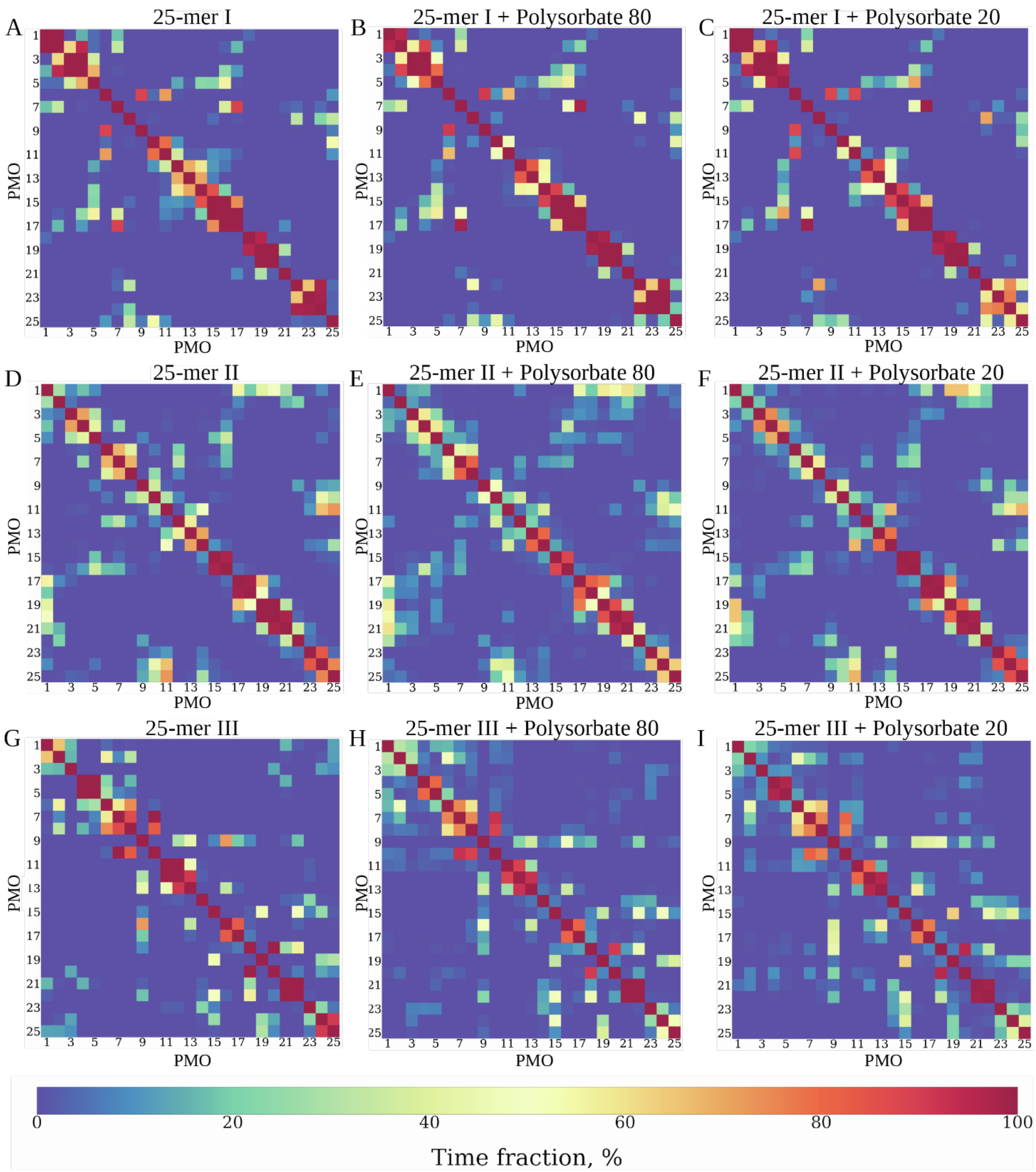

Figure S6. Intramolecular 25-mer-PMO interactions in the presence of Polysorbate 20 and Polysorbate 80. Displayed are maps showing the time fraction of intramolecular interactions between PMO nucleotides obtained from 1-μs MD simulations in the absence of surfactants for the 25-mer PMO conformer I (panel **A**), conformer II (panel **D**) and conformer III (panel **G**), in the presence of Polysorbate 80 for 25-mer PMO conformer I (panel **B**), conformer II (panel **E**) and conformer III (panel **H**), and in the presence of Polysorbate 20 for 25-mer PMO conformer I (panel **C**), conformer II (panel **F**) and conformer III (panel **I**). The numbering for PMO bases starts from the 3'-end of the PMO.

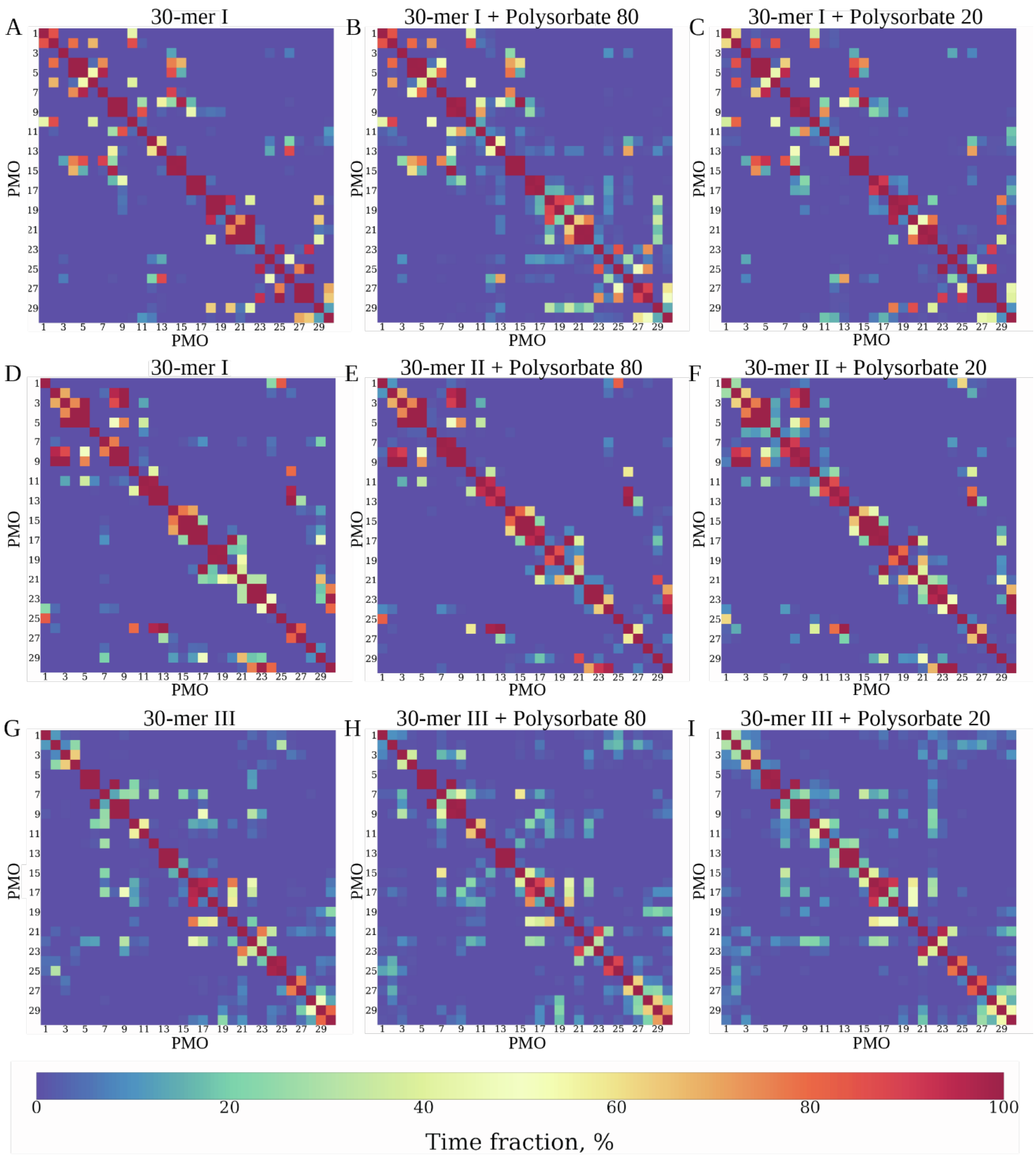

Figure S7. Intramolecular 30-mer-PMO interactions in the presence of Polysorbate 20 and Polysorbate 80. Displayed are maps showing the time fraction of intramolecular interactions between PMO nucleotides obtained from 1-μs MD simulations in the absence of surfactants for the 30-mer PMO conformer I (panel **A**), conformer II (panel **D**) and conformer III (panel **G**), in the presence of Polysorbate 80 for the 30-mer PMO conformer I (panel **B**), conformer II (panel **E**) and conformer III (panel **H**), and in the presence of Polysorbate 20 for the 30-mer PMO conformer I (panel **C**), conformer II (panel **F**) and conformer III (panel **I**). The numbering for PMO bases starts from the 3'-end of the PMO.
