# Supplementary material for: Polysorbates’ effects on molecular and thermodynamic properties of phosphorodiamidate morpholino oligonucleotides’ structures
Source: Mol Ther Nucleic Acids. 2026 Jan 24;37(1):102845. doi: 10.1016/j.omtn.2026.102845 (PMC12914542; doi:10.1016/j.omtn.2026.102845)
Supplement: Document S2. Article plus supplemental information [file mmc6.pdf]

Molecular Therapy
Nucleic Acids
Original Article

Polysorbates' effects on molecular and thermodynamic properties of phosphorodiamidate morpholino oligonucleotides' structures

Evgenii Kliuchnikov,[1,3] Daniel Pierson,[2] Ying Chou,[1] Willow DiLuzio,[2] Kenneth A. Marx,[1] Arani Chanda,[2] and Valeri Barsegov[1]

[1]Department of Chemistry, University of Massachusetts, Lowell, MA 01854, USA; [2]Technical Operations, Sarepta Therapeutics, Cambridge, MA 02142, USA

Elucidating the structure-function relationships of phosphorodiamidate morpholino oligonucleotides (PMOs) is challenging due to limited structural data. We combined surface tension and circular dichroism (CD) spectroscopy with molecular dynamics simulations to investigate how two different PMO molecules interact with Polysorbate 80 and Polysorbate 20. In simulations of 1:1 stoichiometry complexes, we observed strong, staged, concentration-dependent PMO-surfactant interactions, with interaction energies of −60 to −80 kcal/mol for 25-mer conformers and −50 to −70 kcal/mol for 30-mer conformers. Surfactants primarily associate through surface binding but can also insert into PMO structures, preventing unfolding. Structural analysis revealed that position-dependent contacts, especially with hydrophobic surfactant tails, drive these interactions. Importantly, PMO-surfactant binding does not disrupt base pairing, base stacking, or overall chirality, consistent with CD spectra, though it slightly enlarges the tertiary structure. Surfactants reduce solvent exposure of PMO surfaces within complexes, decreasing intermolecular interactions, yet the overall PMO-surfactant complex remains more solvent-exposed. Occasionally, surfactants act in a "chaperone-like" manner, enabling refolding into more compact structures. Together, these findings highlight how surfactants stabilize PMO conformers without disrupting their essential structure. This improved understanding of PMO-surfactant interactions broadens insight into PMO physicochemical behavior and supports the rational design of RNA-mimic therapeutics.

INTRODUCTION

Antisense oligonucleotide (ASO)-based therapeutics are synthetic DNA/RNA mimetics that bind to mRNA *via* traditional complementary base-pairing interactions, but they have physicochemical and biological properties that are different from those of canonical DNA/RNA.[1] Over the last several decades, careful design of these molecules has been very successful, leading to more than a dozen commercially approved drug products to treat or manage a wide range of diseases.[2] Backbone modification of ASOs has been one of the approaches used to overcome the undesired *in vivo* and unfavorable physicochemical and biochemical properties of naturally occurring DNA and RNA.[3,4]

Phosphorodiamidate morpholino oligonucleotides (PMOs) are single-stranded DNA/RNA analogs where the five-membered ribosyl ring is replaced with a six-membered morpholino ring, and phosphate linkages are replaced with uncharged phosphorodiamidates. This backbone makes PMOs charge neutral, and their overall structures make them highly soluble in aqueous medium. Along with their low to no metabolic degradation *in vivo* and high structural stability, these combined properties have made them a key candidate for therapeutic use. PMOs have been approved by the US Food and Drug Administration (FDA) for the treatment of Duchenne muscular dystrophy (DMD) since 2016,[5–7] and PMO backbone-based molecules also have been designed to target Marburg virus, Ebola virus,[8,9] picornaviruses, and other viruses, along with bacterial targets.[10–12] PMO-guanidinium morpholino oligomer (PMO-GMO) chimeras have been developed as potential anti-cancer agents. These chimeras incorporate guanidinium linkages into the morpholino backbone to improve cell permeability and antisense activity without the need for external delivery agents.[13]

While conformational transitions in native biomolecules (DNA, RNA, and proteins) have been studied extensively, little is known about dynamic structural transitions in PMOs, whose backbone structure is distinctly different from canonical nucleic acids. In our previous paper, we presented the first detailed solution structures of 22-mer, 25-mer, and 30-mer PMOs using a combination of experiments, computational molecular modeling, and machine learning.[14] This study showed that conformational dynamics of PMOs are

Received 18 September 2025; accepted 20 January 2026;
https://doi.org/10.1016/j.omtn.2026.102845.

[3]Present address: Department of Biological Engineering, Massachusetts Institute of Technology, Cambridge, MA 02139, USA

Correspondence: Arani Chanda, Technical Operations, Sarepta Therapeutics, Cambridge, MA 02142, USA.
E-mail: achanda@sarepta.com
Correspondence: Valeri Barsegov, Department of Chemistry, University of Massachusetts, Lowell, MA 01854, USA.
E-mail: valeri_barsegov@uml.edu

© 2026 The Authors.
Published by Elsevier Inc. on behalf of The American Society of Gene and Cell Therapy.
This is an open access article under the CC BY license (http://creativecommons.org/licenses/by/4.0/).

defined by the competition between the non-polar faces of nucleobases and uncharged phosphorodiamidate groups for shielding PMOs from solvent exposure. PMO molecules form non-canonical, partially helical, yet folded structures with a small radius of gyration (R_g) and low counts of base pairing and stacking. Intrinsic viscosity data and calculated Huggins constants for the PMOs studied were indicative of potentially extended systems.[14] This understanding of PMO structures and their underlying principles forms a paradigm to delineate the structure-property-function relationships for therapeutic PMOs.

Structure plays a key role in the physicochemical properties of biologically active molecules. Therefore, equipped with the solution-state structures of PMOs and an understanding of the unique conformational dynamics that are at play between the non-polar nucleobases and uncharged phosphorodiamidate groups of these molecules, in this study we focus our attention on understanding their physical behavior further, especially for solution-state properties and their stability. One of the key features that are critical for biomolecules from a manufacturability standpoint is their amphiphilic nature. This property is not only critical for their solution-phase higher-order structure but is also equally important for their interactions with hydrophobic and hydrophilic surfaces, including the air-water interface.[15] This aspect is often used to understand and thereby control their adsorption, aggregation[16] and other key quality attributes. In that regard, one of the most common approaches to control adsorption and aggregation is the use of surfactants.[15,17,18] Surfactants are widely used in the pharmaceutical industry, and their interactions with associated surfaces and with other biomolecules have been described in the literature.[18] Mechanisms of interaction between various biomolecules and surfactants have been shown to depend on the nature of the molecules, the surfaces involved, and the nature of the solution medium, among other factors.[17]

In our previous study,[14] we carried out combined experimental and computational studies of the dynamic structural properties of three separate PMO molecules in aqueous solution, of lengths 22 nucleobases (22-mer), 25 nucleobases (25-mer), and 30 nucleobases (30-mer), which have sequences complementary to target regions of exon 45, exon 53, and exon 51, respectively, of the dystrophin gene pre-mRNA transcript.[1] By correlating the experimental and theoretical circular dichroism (CD) spectra and concentration-dependent viscosity profiles of PMO solutions, we were able to resolve the ensemble of PMO conformer structures that exist in an aqueous solution for these three therapeutic PMOs at room temperature and then to calculate their molecular properties and thermodynamic state functions, including the entropy, enthalpy, and free-energy changes associated with PMO folding in solution.[14] These data indicated that the presence of non-polar nucleobases and the uncharged phosphorodiamidate backbone imparts a unique secondary structure to the PMO molecules, different from canonical charged oligonucleotides. Considering their unique structure, it would be valuable to explore interactions of PMO molecules with surfactants using both experimental and computational modeling approaches. Insights from our study of interactions between PMOs and surfactants provide a unique opportunity to decouple charge interactions and primarily focus on hydrophobic and hydrophilic aspects of PMOs.

In this study, to probe these aspects, we employed surface tension measurements, CD spectroscopy, and a computational modeling approach developed in our previous study,[14] to explore the molecular properties and solution structures of the 25-mer and 30-mer PMOs in the presence of surfactants Polysorbate 80 and Polysorbate 20. We focused computationally on 1:1 complexes of the surfactants with just the 25-mer and 30-mer, not the 22-mer, because while all PMOs exhibited largely similar properties, these two PMOs showed the largest differences.[14] The results obtained provide detailed insight into the way in which one surfactant molecule approaches a PMO, interacts with the individual PMO residues over time, and finally establishes a steady-state energetic interaction in the 1:1 complex. These data help to elucidate the important role played by PMO-surfactant interactions in preventing PMOs from unfolding and, possibly, from aggregating.[16] The structure and energetics insights gained here substantially extend the current understanding of the solution structure-function relationship for PMOs. The results obtained provide unique insights into the protein-like behavior of PMO molecules in their interactions with surfactants, which can be useful in the design of a new generation of RNA-mimic drugs.

RESULTS

25-mer and 30-mer PMOs

The sequences of nucleotides forming the 25-mer and 30-mer PMOs, complementary to exons 53 and 51, respectively, of the Dystrophin gene pre-mRNA, are displayed in Figure 1A. Each sequence shows the total number of nucleobases adenine, cytosine, guanine, and thymine and the percentage of guanine in each sequence. The 25-mer and 30-mer PMO molecules contain a triethylene glycol (TEG) piperazine linker at the 5′-end. Runs of guanines in sequences are known to form preferred secondary structures.[19] Since there are no more than two adjacent guanines in any of the PMO structures, those secondary structures are not expected to form in either the 25-mer or 30-mer PMOs. The chemical structures of Polysorbate 20 and Polysorbate 80 are shown in Figure 1C, for which the extended 3D structures shown in Figure 1D were derived and parametrized as described in the supplemental information. We used v = x = y = z = 5 as the short chain lengths for simulating the surfactant structures. The length L and width W of both Polysorbate 80 (right) and Polysorbate 20 (left), including the central ring (cr1), long, more hydrophobic tail (lt2), and short tails (st3, st4, and st5), are indicated.

Surface tension studies of 25-mer and 30-mer PMOs with Polysorbate 80 and Polysorbate 20

The molecular interplay between surfactants and biological molecules, largely proteins, contributing to changes in the air-water interface, has been described by Lee et al, Gunning et al., and Arsiccio et al.[20–22] A stepwise equilibrium behavior is expected to be observed with increasing surfactant concentration, and simple surface tension

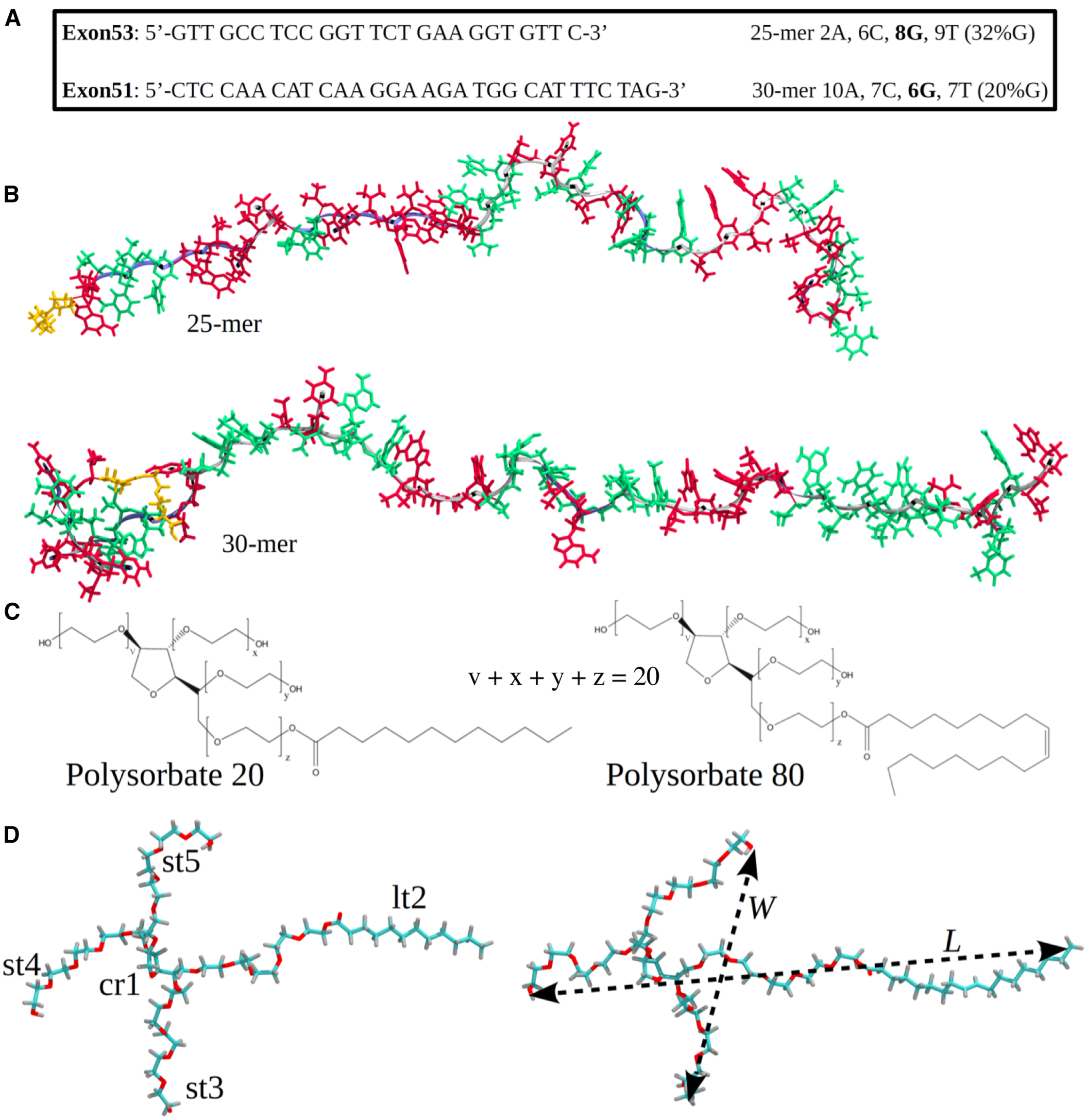

Figure 1. PMO and surfactant structures

Shown for two therapeutic PMOs, Exon53 (25-mer PMO) and exon51 (30-mer PMO), are their primary sequences (A) and the unfolded (reference) structures (B). Each sequence is shown with its base composition, the total amount of nucleobases—adenine (A), cytosine (C), guanine (G), and thymine (T), and the relative amount of G, which is bolded (percentage). The conformers are shown in Licorice representation (sticks) and in Twister representation (blue line) describing the backbone. The TEG piperazine linker is shown in orange. A and T bases are shown in green, whereas C and G bases are shown in red. Based on the chemical structures of Polysorbate 20 and Polysorbate 80 (C), the 3D structures were derived (D) and parametrized (see SI). We used v = x = y = z = 5 as chain lengths for simulating the surfactant structures. The length *L* and width *W* of surfactant Polysorbate 80 (right) and the structure elements of Polysorbate 20 (left), including the central ring (cr1), long tail (lt2), and short tails (st3, st4, and st5), are illustrated in (D).

titration measurements can be used to estimate this behavior via changes in surface tension. The different stages of interactions are described schematically in Figure 2A by regions 2–5 for constant PMO concentration as surfactant concentration increases. The existence of region 1 has been mentioned in the literature; however, this very low concentration region was not included in the figure, as this was not observed in our analysis. These steps, described in Figure 2A, are as follows. Region 1 (very low[21,22] surfactant concentration, not shown): trace surfactant concentrations have little impact. Region 2 (low surfactant concentration): surface tension decreases as surfactant molecules occupy empty sites at the air-water interface. Region 3 (moderate surfactant concentration): surfactant concentration reaches a range where interaction with PMO is energetically favored, and hence surface tension plateaus as surfactant loads the

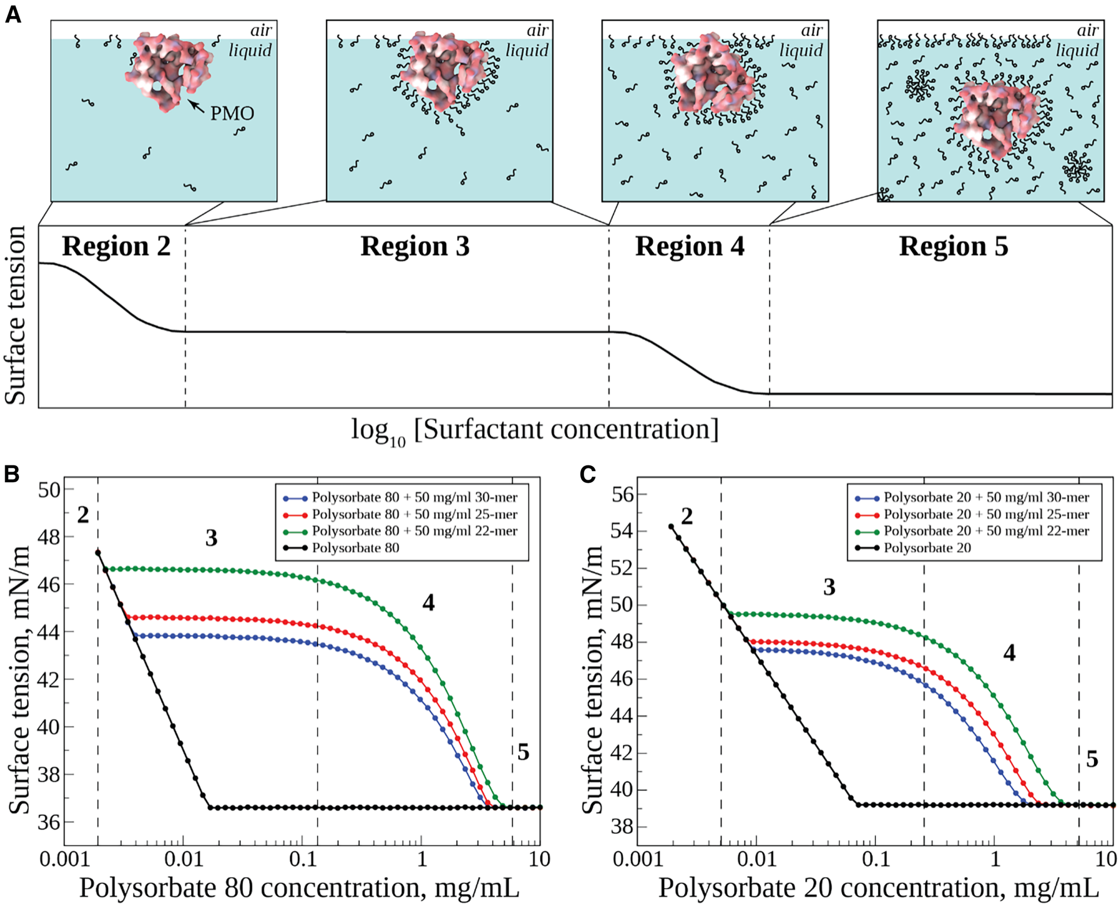

Figure 2. Surface tension titration of PMOs with Polysorbate 20 or Polysorbate 80

An idealized representation of four titration regions showing gradual changes is displayed, with a visual representation of PMO and Polysorbate molecules (not to scale; A) along with similar experimental data for Polysorbate 80 (B) and Polysorbate 20 (C), either alone or interacting with PMOs.

PMO, not the air-water interface. Region 4 (high surfactant concentration): interactions of surfactants with PMO are sufficient for its displacement from the air-water interface, and it is energetically favorable for surfactant molecules to interact with the interface, thereby decreasing the interfacial tension. Region 5 (very high surfactant concentration): a second plateau is reached when no further surfactants can absorb to the air-water interface since the critical micelle concentration (CMC) is reached, and surfactant molecules load the micellar structures. In the absence of interacting biomolecules, the CMC of the surfactant is expected to be in the moderate range (region 3). In the presence of interacting biomolecules, the CMC value significantly increases due to surfactants interacting with biomolecules in a predictable and well-behaved manner (region 5).

Results from air-water interfacial surface tension studies using Polysorbate 80 and Polysorbate 20 with three different PMO molecules (22-mer: from our earlier study[14] and not further studied here but used to establish the PMO size trend; 25-mer and 30-mer) are shown in Figures 2B and 2C, respectively. Equilibrium surface tension was measured for varying Polysorbate 80 and Polysorbate 20 concentrations in the 0.002–10 mg/mL range in phosphate-buffered saline (PBS) medium at 25°C in the absence of PMO (Figures 2B and 2C). The CMC values, representing the sharp negative-slope to zero-slope boundary points, were calculated to be 0.02 mg/mL and 0.07 mg/mL, respectively, for Polysorbate 80 and Polysorbate 20 (Table S1). These values are similar to those previously reported in the literature.[23,24] Surface tension results from titrations of Polysorbate 80 and Polysorbate 20 into PMO solutions do not overlay with those of surfactant solutions alone, showing remarkably distinct regions 2–5, very similar to the idealized experimental diagram in Figure 2A. This indicates that the surfactants interact with PMO molecules in a specific and well-defined way, much as proteins do in similar surface tension experiments. Analysis of Figures 2B and 2C shows that in both cases an initial steep drop in surface tension (region 2) was observed, mimicking the results for surfactant alone. This was followed by an abrupt departure from the surfactant-only curve that described a plateau (region 3), transitioning to a gradual drop (region 4), and leading to the final plateau (region 5) that

matched the surfactant-only surface tension values. Region 1, corresponding to very low surfactant concentrations, was not observed under the experimental conditions used. While the overall features are the same for both surfactants, some clear differences exist. The transition from region 2 to region 3 was significantly delayed for Polysorbate 20 relative to Polysorbate 80, indicating qualitatively that Polysorbate 80 interacts more strongly with PMO molecules than Polysorbate 20.

The inflection point between region 2 and region 3 is sometimes referred to as the critical aggregation concentration (CAC) to highlight the fact that, at this concentration, surfactants begin to interact with another molecule or component present in the system. Table S1 lists CAC values for all six systems measured. PMO molecules are somewhat amphiphilic in nature, as a drop in surface tension is observed when PMO molecules are added to the PBS buffer. However, this initial drop is significantly lower compared to the surfactant-dependent changes observed in these studies and is, in fact, already accounted for in the initial concentration surface tension values. All CAC values associated with Polysorbate 80 are between 0.002 and 0.004 mg/mL, while CAC values associated with Polysorbate 20 are between 0.006 and 0.009 mg/mL, clearly indicating that Polysorbate 80 begins to interact with PMO molecules at much lower concentrations compared to Polysorbate 20. CMC values for the same set of analyses are also included in Table S1. Similar Polysorbate 80 CMC values were observed for the 25-mer and 30-mer, while a slightly higher value was observed for the 22-mer PMO. The trend was the same for Polysorbate 20, although slightly lower values were observed in that case. While CAC and CMC values are reported in mg/mL, all three PMOs' concentrations, irrespective of their molecular weights, were fixed at 50 mg/mL, where the molar concentrations corresponded to 4.9, 5.8, and 6.6 mM for the 30-mer, 25-mer, and 22-mer, PMOs, respectively. To estimate the stoichiometry of interaction between these PMO and surfactant molecules, their ratios at CMC were measured on a molar basis. This value, *n*, measured as the ratio of Polysorbate molecules to PMO at CMC, is reported in Table S1. These values vary between approximately 1.7 and 2.9 and describe a somewhat inverse relationship relative to the surfactant CMC values. While these numbers are not significantly different for the two surfactants, the overall trends suggest that the interaction is well defined within these two sets of molecules.

CD spectroscopy of 25-mer and 30-mer PMOs with Polysorbate 80 and Polysorbate 20

To assess whether surfactants interacting with PMOs cause disruptions in chirality due to changes in their folding patterns, we carried out CD spectroscopy experiments. Previously, we determined the CD spectra of all three PMOs—the 22-mer, 25-mer, and 30-mer.[14] Their CD spectra were all very similar and resembled an A-type canonical RNA helical spectra, with dominant features of right-handed chirality (trough below 250 nm and peak at 275 nm), due to the interacting bases evident in simulations of the PMOs' solution structures. Since these three PMOs' CD spectra were similar, we decided to carry out CD measurements for only one PMO, the 30-mer, in the presence of either Polysorbate 80 or Polysorbate 20. In Figure 3A we present three CD spectra: the 30-mer PMO alone at 0.04–0.06 mg/mL and the 30-mer at the same concentration in the presence of Polysorbate 80 at two concentrations, 0.02 mg/mL and 0.2 mg/mL. In Figure 3B, we present the CD spectra of the 30-mer alone again and in the presence of Polysorbate 20 at 0.2 mg/mL. The CD spectra for both the Polysorbate 80:30-mer complex and the Polysorbate 20:30-mer complex are nearly identical to that of the 30-mer alone, even at the large surfactant molar excess condition of 0.2 mg/mL. These data indicate that both surfactants interact with the 30-mer PMO in a way that causes no significant changes in the chiral properties of its folded solution conformers, either in the backbone or in the bases.

Computational modeling of Polysorbate 80 and Polysorbate 20 and the 25-mer and 30-mer PMO-surfactant complexes

Based on the results of surface tension studies, we conducted computational modeling only on the 25-mer and 30-mer PMOs, focusing on conditions below the surfactant concentrations corresponding to region 5 (Figure 2A), where PMO-surfactant complexes with 1:1 stoichiometry would form (Table S1). Since surfactants are always added, in practice, to pre-existing PMOs solutions, we restricted our modeling to the folded PMO conformers interacting with added surfactants. The focus on simulating only 1:1 complexes of the surfactants with PMOs is for several reasons: (1) we could estimate the 1:1 PMO-surfactant binding energies; (2) we could clearly understand the 1:1 binding pattern and PMO site preferences for the interacting surfactant molecules without the complications arising from multiple surfactant molecules interacting with the PMO simultaneously; and (3) from a purely practical standpoint, simulating 2–3 or more surfactant molecules per PMO molecule would require a prohibitive simulation time.

In our previous study of PMO molecules,[14] we employed the RNA force field $bsc0_{\chi OL3}$ with improved torsion angles as the basis, and we used general Amber force field (GAFF) to account for atoms not described in $bsc0_{\chi OL3}$, i.e., the atoms forming the morpholino ring, phosphorodiamidate linkage, and TEG piperazine linker (Figure 1; materials and methods). In this study, we extended the description of solution PMOs by taking into account the surfactant molecules Polysorbate 20 and Polysorbate 80, including the interactions between the folded 25-mer and 30-mer PMO conformers and these surfactants. Calculation of atomic partial charges in Polysorbate 20 and Polysorbate 80 (Figure S1) and development of the atomic force field parameters for the surfactants are described in the SI (Tables S2–S4). Reconstruction of the initial structures of PMO molecules (Figure 1B), along with Polysorbate 20 and Polysorbate 80 (Figure 1D) is described in materials and methods.

For each Polysorbate 20 and Polysorbate 80, we generated 2 independent 1-μs long molecular dynamics (MD) simulation runs (2 μs for each molecule) using their extended conformations as initial structures (Figure 1D). In our previous study,[14] we found that the existence of multiple different 25-mer and 30-mer PMO conformations

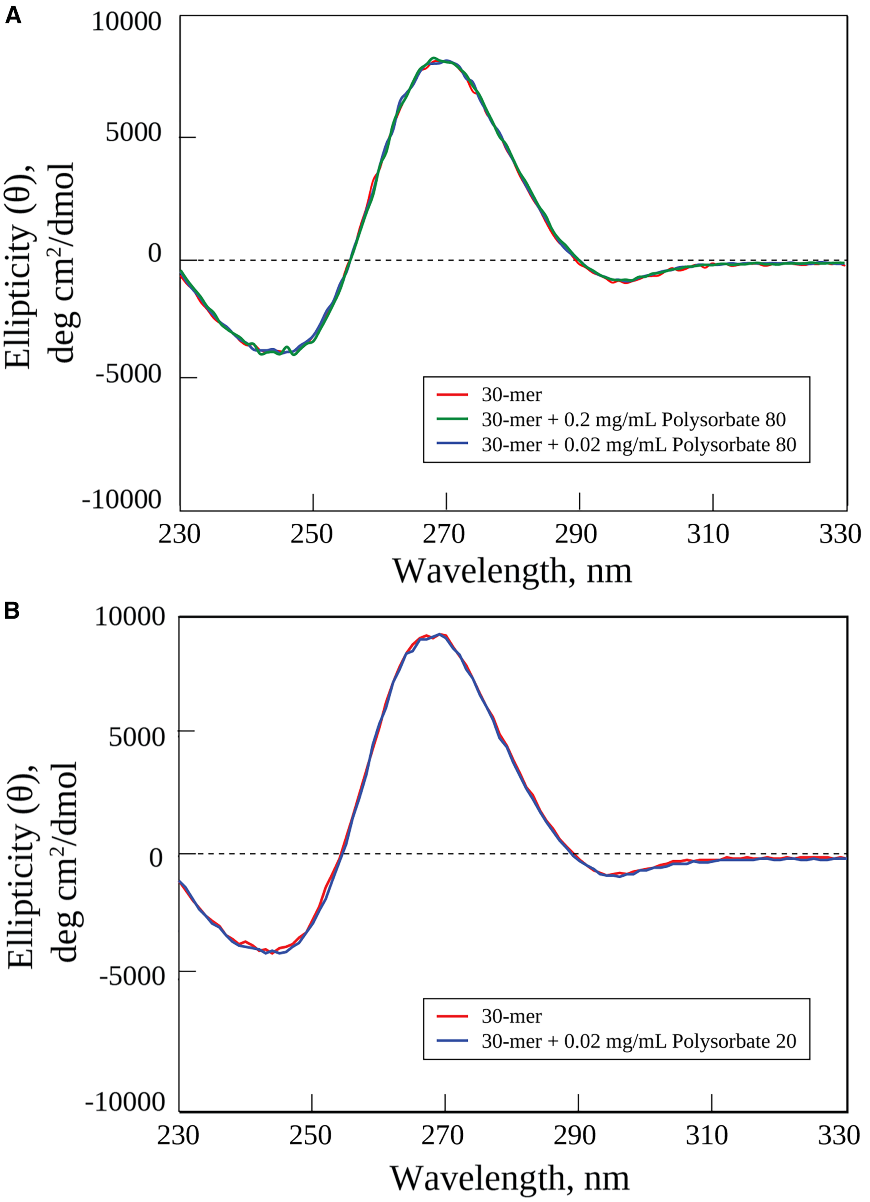

Figure 3. Experimental CD spectra

Shown are circular dichroism profiles collected at 25°C for the 30-mer PMO, the 30-mer PMO in 0.2 mg/mL Polysorbate 80 solution, and the 30-mer PMO in 0.02 mg/mL Polysorbate 80 solution (A); and the 30-mer PMO and the 30-mer PMO in 0.2 mg/mL Polysorbate 20 solution (B). All solutions contained 30-mer PMO at 0.04–0.06 mg/mL.

give rise to distinctly different CD profiles. Therefore, we used non-linear regression to fit the ensemble of theoretical CD spectral curves to the average experimental CD spectrum in order to resolve the most relevant solution structures, which we refer to as "principal solution conformers," and to evaluate their weights in the statistical ensemble. In this work, we used the principal solution conformers I–III for the 25-mer and 30-mer PMOs to explore PMO-surfactant interactions. For each PMO plus surfactant system, *i.e.*, 25-mer PMO with Polysorbate 80, 25-mer PMO with Polysorbate 20, 30-mer PMO with Polysorbate 80, and 30-mer PMO with Polysorbate 20, we generated 10 independent 1-μs long MD simulation runs (10 μs for each system), using the folded conformations of the 25-mer and 30-mer PMO and extended conformations of the surfactants as initial structures (Figure S2B). Videos S1 and S2 provide visualizations highlighting the structural fluctuations between interacting PMOs and surfactant molecules.

We profiled R_g, the numbers of base pairs N_{bp} and base stacks N_{bs}, the solvent-accessible surface area *SASA*, and the root-mean-square deviation *RMSD* (Table 1). For Polysorbate 80 and Polysorbate 20 (both as single molecules and in PMO-surfactant complexes), we profiled R_g, number of hydrogen bonds n_{hb}, *SASA*, *RMSD*, and molecular length *L* and width *W* (Table S5; Figure 1D). Moreover, for the complexes of surfactants with PMOs, we calculated the *SASA* of the complexes (Table 1). R_g for PMOs (and *L* and *W* for surfactants) provides information about the spatial distribution of atoms in a molecule in 3D space, whereas N_{bp} and N_{bs} for PMOs (and n_{hb} for surfactant molecules) reflect the propensity to form secondary structure. While *RMSD* measures molecular structural variability, *SASA* quantitates the amount of molecular surface exposed to solvent.

Structure fluctuations in Polysorbate 80 and Polysorbate 20 and in 25-mer and 30-mer PMOs

First, we explored the solution properties of isolated Polysorbate 20 and Polysorbate 80 (i.e., without PMOs). These can be viewed as control experiments *in silico* for subsequent analysis of the structures of the 25-mer and 30-mer PMOs in the presence of these surfactants. After the surfactant molecules transform from extended conformations to collapsed conformations (see Video S3 for Polysorbate 80), the molecular structure characteristics fluctuate around their average values for both surfactants (Figure S3; Table S5). R_g fluctuates around 0.8 nm for both Polysorbate 20 and polysorbate 80; *RMSD* varies around 1.2 nm for Polysorbate 80 and 1.00 nm for Polysorbate 20. *SASA* fluctuates around 1,760 Å^2 for Polysorbate 80 and around 1,700 Å^2 for Polysorbate 20 (Figure S3). *RMSD* is the only parameter that shows any sizable difference between Polysorbate 20 and Polysorbate 80; n_{hb}, *L*, and *W* gravitate toward similar average values ($n_{hb} \approx 0.1$,

Table 1. Structural characteristics of PMO molecules obtained from all-atom MD simulations of PMOs with and without surfactants

PMO	Surfactant	R_g, nm	N_{bp}	N_{bs}	$SASA$, Å^2	$SASA_{tot}$, Å	RMSD, nm
25mer I	Polysorbate 80	1.34 ± 0.07	9.6 ± 1.5	6.0 ± 1.6	4,609 ± 244	5,895 ± 328	0.35 ± 0.13
25mer I	Polysorbate 20	1.33 ± 0.05	9.5 ± 1.5	6.0 ± 1.7	4,406 ± 266	5,740 ± 336	0.31 ± 0.10
25mer I	w/o surfactant	1.26 ± 0.02	9.8 ± 1.4	6.1 ± 1.6	4,967 ± 193	N/A	0.31 ± 0.11
25mer II	Polysorbate 80	1.31 ± 0.08	7.3 ± 2.4	4.5 ± 1.9	4,402 ± 340	5,671 ± 374	0.73 ± 0.25
25mer II	Polysorbate 20	1.31 ± 0.06	8.3 ± 2.0	4.1 ± 1.9	4,458 ± 336	5,634 ± 391	0.59 ± 0.11
25mer II	w/o surfactant	1.21 ± 0.04	7.9 ± 1.9	3.9 ± 1.6	4,737 ± 271	N/A	0.57 ± 0.22
25mer III	Polysorbate 80	1.43 ± 0.12	7.3 ± 1.6	5.6 ± 2.4	4,433 ± 267	5,729 ± 352	0.85 ± .20
25mer III	Polysorbate 20	1.38 ± 0.10	6.8 ± 2.0	5.7 ± 1.9	4,423 ± 267	5,669 ± 361	0.84 ± 0.17
25mer III	w/o surfactant	1.34 ± 0.09	8.0 ± 1.4	5.6 ± 1.8	4,757 ± 203	N/A	0.75 ± 0.22
30mer I	Polysorbate 80	1.47 ± 0.07	7.8 ± 1.6	9.5 ± 3.0	5,224 ± 244	6,428 ± 349	0.59 ± 0.15
30mer I	Polysorbate 20	1.44 ± 0.07	7.3 ± 1.7	10.3 ± 3.2	5,058 ± 276	6,219 ± 367	0.56 ± 0.26
30mer I	w/o surfactant	1.42 ± 0.06	7.7 ± 1.5	9.9 ± 2.6	5,599 ± 174	N/A	0.56 ± 0.17
30mer II	Polysorbate 80	1.47 ± 0.05	10.5 ± 1.5	6.1 ± 1.5	5,017 ± 183	6,319 ± 321	0.33 ± 0.06
30mer II	Polysorbate 20	1.49 ± 0.05	10.5 ± 1.4	6.3 ± 1.8	5,655 ± 612	6,386 ± 354	0.42 ± 0.12
30mer II	w/o surfactant	1.42 ± 0.05	10.2 ± 1.3	6.3 ± 1.6	5,493 ± 172	N/A	0.45 ± 0.16
30mer III	Polysorbate 80	1.47 ± 0.07	6.6 ± 1.7	5.8 ± 2.0	5,347 ± 273	6,531 ± 441	0.85 ± 0.12
30mer III	Polysorbate 20	1.50 ± 0.07	7.4 ± 1.9	5.3 ± 1.9	5,412 ± 240	6,586 ± 409	0.97 ± 0.23
30mer III	w/o surfactant	1.51 ± 0.10	6.9 ± 1.8	4.7 ± 1.8	5,928 ± 263	N/A	0.85 ± 0.25

Shown for each principal solution conformer I–III of 25-mer and 30-mer PMOs are averages and standard deviations for the radius of gyration R_g, number of base pairs N_{bp} number of base stacks N_{bs}, solvent accessible surface area for PMO only (*SASA*) and for PMO-surfactant complex ($SASA_{tot}$), and root-mean-square deviation *RMSD*.

$L \approx 1.5$ nm and $W \approx 1.6$ nm) for both surfactants (Table S5). A low count of internal hydrogen bonds (Figure S3D) points to a weak propensity of surfactants to form any local or secondary structure. The standard deviations for all the quantities considered (R_g, *RMSD*, *SASA*, n_{hb}, *L*, and *W*) are quite large, implying large-amplitude structural variations for both Polysorbate 80 and Polysorbate 20 in solution (Table S5). These results show that Polysorbate 20 and Polysorbate 80 do not form any stable folded structures.

To better understand the structural variability of Polysorbate 80 and Polysorbate 20, we display in Figure S2A, as an example, typical structures of Polysorbate 80, of which there are many. We used a total of 500 structure snapshots of Polysorbate 80 to construct a scatterplot of R_g vs. *RMSD* and *SASA* (Figure S2B) and support vector machines (SVM; see SI) to separate these structures into two classes of "extended structures" and "collapsed structures". A Polysorbate 80 structure is categorized as 'extended' if $R_g > 0.84$ nm and $RMSD < 1.1$ nm; otherwise, it is considered "collapsed." As Figure S2B shows, there is a large number of structure snapshots for Polysorbate 80 (389 structures; ~78%) that form a dense set centered around the average collapsed conformation, characterized by small *SASA* and R_g but large *RMSD*. There is also a large number of Polysorbate 80 structures that form extended conformations (111 structures; ~22%; see Figure S2B). We obtained a similar scatterplot for isolated Polysorbate 20 (data not shown). These findings reiterate our earlier results, namely that neither Polysorbate 20 nor Polysorbate 80 forms any stable folded solution structures and that these molecules undergo rapid conformational fluctuations (see Video S3).

Next, we explored the properties of Polysorbate 20 and Polysorbate 80 in their interactions with the 25-mer and 30-mer PMO principal solution conformers I–III (Table 1) and compared these properties with those of isolated Polysorbate 20 and Polysorbate 80 (Table S5). R_g, *SASA*, *RMSD*, *L*, and *W* all change, but n_{hb} does not, when Polysorbate 20 and Polysorbate 80 form complexes with PMOs. R_g, *RMSD*, *L*, and *W* increase, and *SASA* decreases for surfactants in all four complexes between Polysorbate 80 or Polysorbate 20 and 25-mer or 30-mer PMOs. The increased values of R_g, *RMSD*, *L*, and *W* indicate that the surfactant molecules are more extended and undergo larger structure fluctuations when they form the PMO-surfactant complex, compared to free surfactants in solution. The decreased values of *SASA* imply that Polysorbate 80 and Polysorbate 20, through their interactions with the PMOs, are overall less exposed to solvent (water). The fact that n_{hb} remains small for free and PMO-bound Polysorbate 80 and Polysorbate 20 implies that these surfactant molecules remain unstructured in both the free and PMO-bound forms.

We present in Figures 4A and 4C snapshots of Polysorbate 80 with the 25-mer and Polysorbate 80 with the 30-mer, respectively, prior to complex formation; then, following complex formation, snapshots of the respective complexes are illustrated in Figures 4B and 4D. While the structure differences in PMOs prior to complex formation

are not easily discernable compared to their structures in the complexes, Polysorbate 80 prior to complex formation can be seen to be more compact compared to their extended structures within the complexes, in agreement with the data in Table S5. In the complexes, the extended Polysorbate 80 can often be seen interacting with a large portion of the PMO surface, and in some cases, clearly "tying" together the ends of the PMO molecule (Figure 4B, conformer III).

Dynamic structural transitions in PMO sequences with Polysorbate 80 and Polysorbate 20

Next, we explored the dynamic structural properties of the 25-mer and 30-mer PMO principal solution conformers I, II, and III within the PMO-surfactant complexes (Figures 4B and 4D). We analyzed the time profiles for R_g, N_{bp}, N_{bs}, and *SASA* for the PMOs in the PMO-surfactant complexes, as well as the *SASA* values of the whole PMO-surfactant complexes.

In Figure 5 we display, as an example, the profiles of R_g, N_{bp}, N_{bs} and *SASA* for the 25-mer PMO conformer I, with and without Polysorbate 80. The time profile of *SASA* for the 25-mer PMO alone lies above the *SASA* profile for the 25-mer PMO with Polysorbate 80 (Figure 5A), implying that interacting Polysorbate 80 partially shields the 25-mer PMO molecule, thereby making the PMO less accessible to solvent. The time profile of R_g for the 25-mer PMO alone lies below that of the 25-mer PMO in the presence of Polysorbate 80 (Figure 5A), indicating that the PMO molecule slightly swells in the presence of Polysorbate 80, thereby increasing its size. This occurs because Polysorbate 80 interacts with the 25-mer PMO, at least in part, not only by inserting its flexible arms (Figure 4B) into the PMO molecule but also by remaining extended as it interacts (Table S5). We found similar results for the 25-mer PMO conformers II and III (data not shown). Structure snapshots of the 25-mer PMO conformers I, II, and III interacting with Polysorbate 80 at different times during the simulations are displayed in Figure 4B. These snapshots illustrate that Polysorbate 80 binds in an extended form to the 25-mer PMO, either by making surface contacts (weak coupling; see snapshots for conformer III in Figure 4B) or by partially inserting itself into the PMO structure (stronger coupling; see snapshots for conformers I and II in Figure 4B). By contrast, the time profiles of N_{bp} and N_{bs} for the 25-mer PMO conformer I, with and without Polysorbate 80, are very similar in magnitude (Figure 5C), implying that Polysorbate 80 does not perturb the secondary structure of the 25-mer PMO (Figure S6). We observed similar results for the 25-mer PMO conformers II and III (data not shown).

R_g, N_{bp}, N_{bs}, and *SASA* for the 30-mer PMO conformer I, with and without Polysorbate 80, are profiled in Figures 5B and 5D. We observe the same trends as in the 25-mer PMO case, namely a higher *SASA* level but lower values of R_g (Figure 5B), and yet similar levels of both N_{bp} and N_{bs} for the PMO molecule without Polysorbate 80 compared to the case of PMO with surfactant (Figure 5D). We obtained similar results for R_g, N_{bp}, N_{bs}, and *SASA* for the 30-mer PMO conformers II and III interacting with Polysorbate 80 (data not shown). Snapshots of the 30-mer PMO conformers I, II, and III interacting with Polysorbate 80 at different times in the simulations are displayed in Figure 4D. These show extended Polysorbate 80 making weak surface contacts (snapshots for conformers I and II in Figure 4D) and stronger interactions (snapshot for conformer III in Figure 4D) with the 30-mer PMO and Polysorbate 80. We

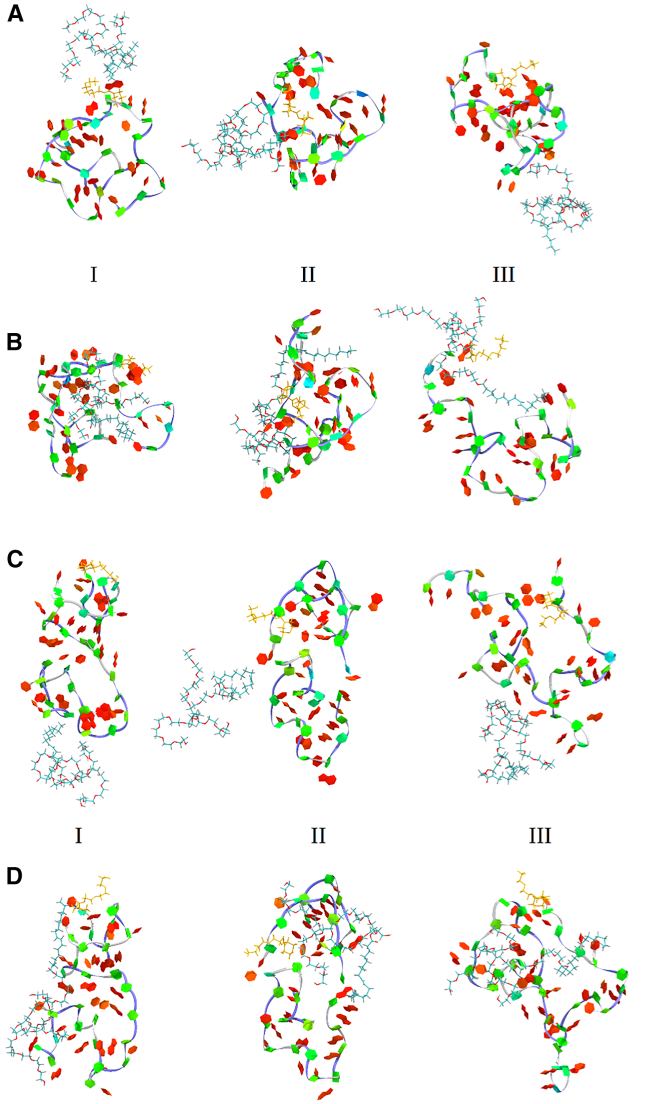

Figure 4. 25-mer and 30-mer PMO principal solution conformers interacting with Polysorbate 80

Snapshots of the initial structures of 25-mer PMOs before interaction with Polysorbate 80 (A) and 30-mer PMOs before interaction with Polysorbate 80 (C), and after 25-mer PMOs have interacted with Polysorbate 80 (B) for 550 ns (conformer I), 650 ns (conformer II), and 700 ns (conformer III), and after 30-mer PMOs have interacted with Polysorbate 80 (D) for 250 ns (conformer I), 450 ns (conformer II), and 150 ns (conformer III). All PMO structures are shown in the twister representation (blue line along the backbone) and in PaperChain representation (for nucleobases). Surfactant molecules are shown in the Licorice representation, and The TEG piperazine linker is colored yellow.

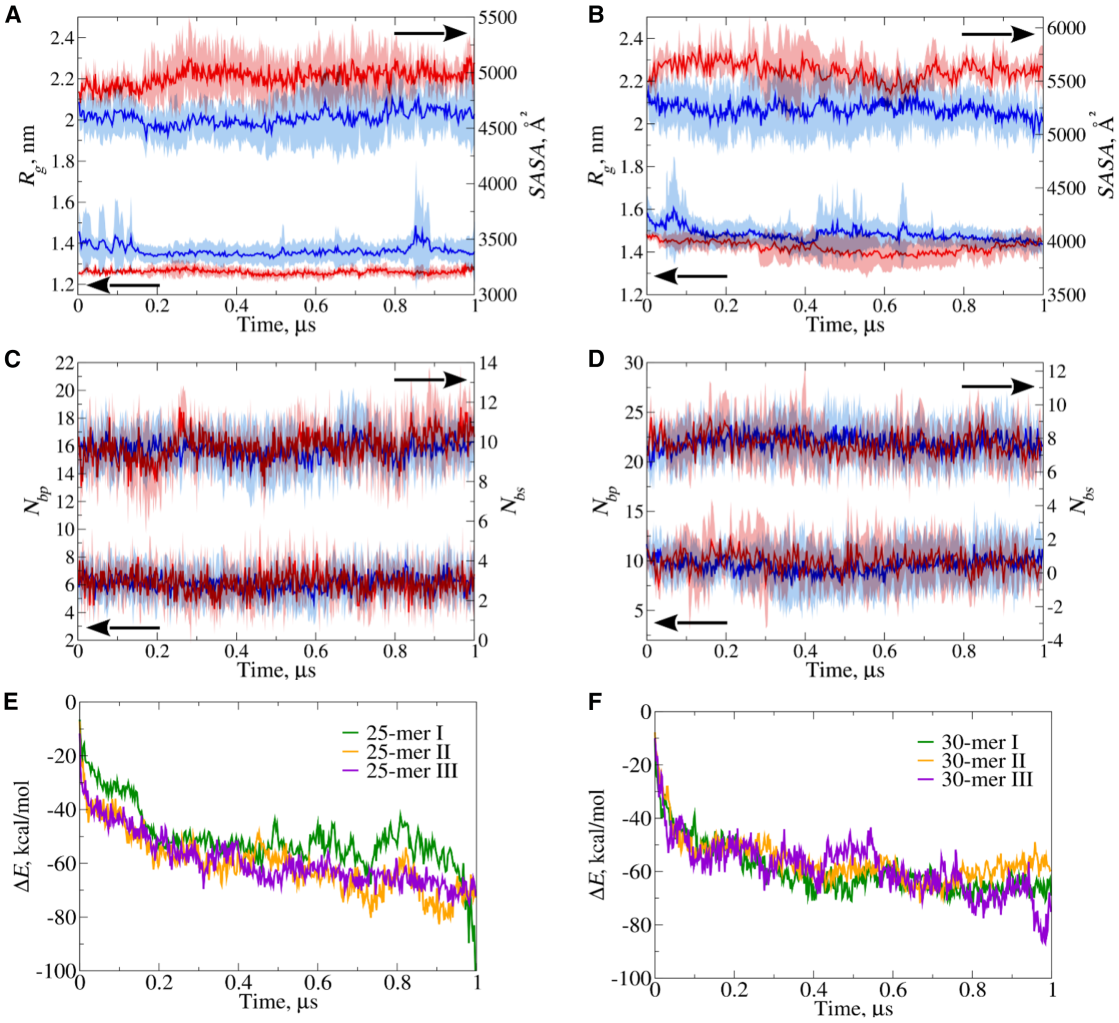

Figure 5. Structural properties and interaction energies of PMOs with and without Polysorbate 80

(A)–(D) show time profiles of structural properties for 25-mer and 30-mer PMO conformation I, with (blue curves) and without (red curves) Polysorbate 80: radius of gyration R_g (A, B; left *y* axes), solvent-accessible surface area (SASA; A, B; right *y* axes), numbers of base pairs N_{bp}(C, D; left *y* axes), and base stacks N_{bs}(C, D; right *y* axes). (E) and (F) display the time evolution of interaction energy ΔE between Polysorbate 80 and PMOs, averaged over 10 all-atom MD simulations. Panel E shows results for the 25-mer PMO (conformers I: green, II: orange, III: purple); (F) shows the data for the 30-mer PMO. PMO–Polysorbate 20 interaction energies are shown in Figure S4.

also probed the PMO-surfactant interactions for the 25-mer and 30-mer PMO molecules in the presence of Polysorbate 20. The results are quite similar to those obtained for Polysorbate 80 (data not shown). These results reiterate that, for the 30-mer PMO, our findings are similar to those of the 25-mer PMO described above, pointing to the existence of nondisruptive contacts in the PMO-surfactant interactions.

Energetics of interactions of PMO sequences with Polysorbate 80 and Polysorbate 20

The results of all-atom MD simulations for the 25-mer and 30-mer PMOs with Polysorbate 80 and Polysorbate 20 demonstrate the importance of nondisruptive surface intermolecular contacts in PMO-surfactant interactions (Figures 4 and 5). This motivated us to explore the energetics of interactions of the 25-mer and 30-mer PMOs with Polysorbate 80 and Polysorbate 20. In Figure 5, we display the time profiles of the average interaction energy, ΔE, for the 25-mer (Figure 5E) and 30-mer PMO conformers I–III (Figure 5F) with Polysorbate 80.

Initially, at time $t = 0$, when there are no interactions between the PMO molecules and Polysorbate 80 molecules (Figures 4A and 4C), $\Delta E = 0$. As soon as the PMO-surfactant interactions develop (Figures 4B and 4D), ΔE starts to decrease (i.e., becomes increasingly negative) over time, attaining nearly constant values at $t = 1$ μs of $\Delta E \approx -60$ to -80 kcal/mol for the 25-mer PMO conformers I–III (Figure 5E) and $\Delta E \approx -50$ to -70 kcal/mol for the 30-mer PMO conformers I–III (Figure 5F). Because stronger PMO-surfactant

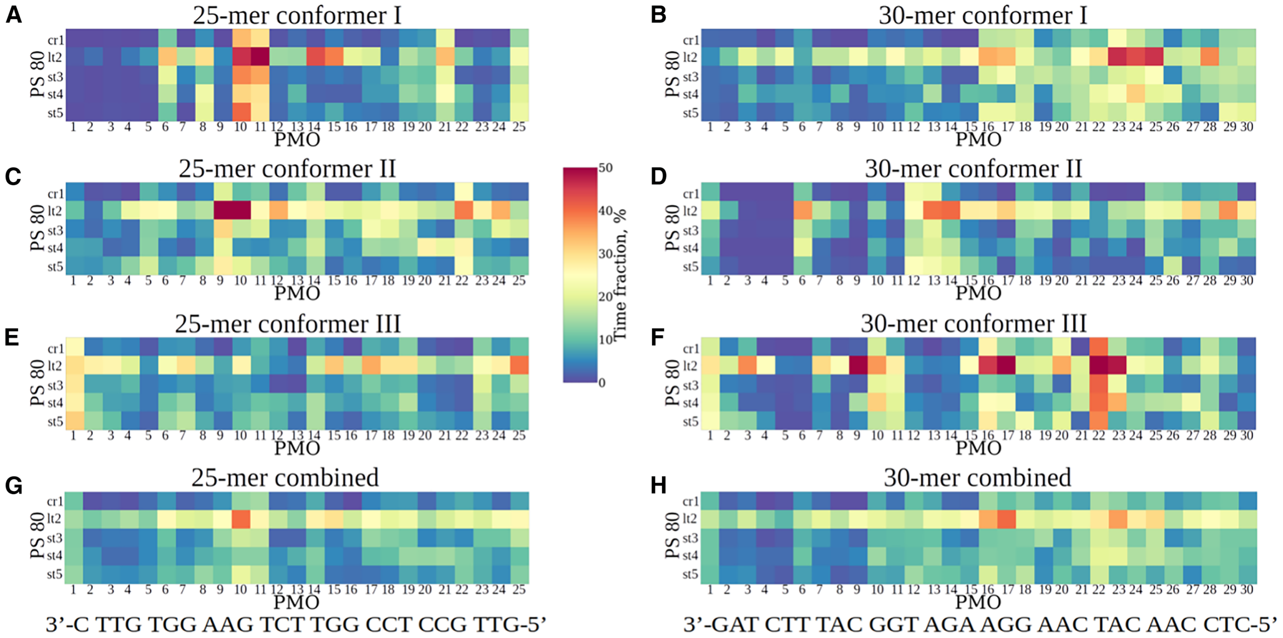

Figure 6. PMO-Polysorbate 80 interaction maps for 25-mer and 30-mer PMOs

Displayed are maps showing the time fraction (percentage) of interactions between PMO nucleotide positions and surfactant side chains for the 25-mer PMO conformer I (A), conformer II (C), and conformer III (E), as well as for the 30-mer PMO conformer I (B), conformer II (D), and conformer III (F). Also shown are the interactions maps for all three conformers of the 25-mer (G) and 30-mer (H) combined. The nomenclature for PMO bases starts from the 3′-end of the PMO, and for the surfactant, cr1 is the central ring (see Figure 1D), lt2 is the long tail (see Figure 1D), and st3, st4, and st5 are the short tails (see Figure 1D). The PMO-Polysorbate 20 interaction maps are shown in Figure S5. PS stands for Polysorbate. The time-fraction vertical scale is displayed in the middle.

interactions correspond to more negative ΔE values, this implies that the strength of PMO-surfactant interactions increases over time. The statistics of ΔE (i.e., the average values and standard deviations) for all PMO-surfactant systems are summarized in Table S6. For example, for the 25-mer PMO conformers I–III interacting with surfactants, ΔE varies in the −55 to −64 kcal/mol range for Polysorbate 80 and in the −53 to −63 kcal/mol range for Polysorbate 20. The small ∼1–2 kcal/mol difference in interaction energy between the surfactants likely arises because Polysorbate 80 is slightly larger in size than Polysorbate 20 (Figures 1D and S1), and there is also minor variability in ΔE values among different conformers (Table S6).

To understand the origin of the large PMO-surfactant interaction energies observed for the 25-mer (−60 to −80 kcal/mol) and 30-mer (−50 to −70 kcal/mol) PMOs at the molecular level, we analyzed the individual energy contributions to ΔE, including changes in van der Waals energy (ΔE_{vdW}), electrostatic energy (ΔE_{el}), and solvation energy (ΔE_{solv}). The energy statistics for ΔE_{vdW}, ΔE_{el}, and ΔE_{solv} are presented in Table S6. These data show that ΔE_{solv} is large and positive (40 to −54 kcal/mol), implying unfavorable changes in solvation energetics when the 25-mer and 30-mer PMOs interact with Polysorbate 80 and Polysorbate 20, compared with the separate PMO and surfactant molecules. ΔE_{vdW} and ΔE_{el} are quite large and negative: ΔE_{vdW} varies between −86 and −66 kcal/mol, and ΔE_{el} varies between −31 and −21 kcal/mol (Table S6). These numbers imply favorable changes in excluded-volume interactions (ΔE_{vdW}) and Coulombic interactions (ΔE_{el}). Overall, the energy analyses indicates strong PMO-surfactant interactions for all 25-mer and 30-mer PMO conformers with both Polysorbate 80 and Polysorbate 20 (Figure 5). The favorable changes in steric interactions and electrostatic couplings in the PMO-surfactant complexes vs. separate PMO and surfactant molecules outweigh the unfavorable changes in solvation energy for the PMO-surfactant complex, as compared to the isolated PMO and surfactant molecules.

Interaction patterns and binding sites for PMO-PMO and PMO-surfactant interactions

We explored the structural basis for strong PMO-surfactant interactions between the 25-mer and 30-mer PMO conformers and Polysorbate 80 and Polysorbate 20. In the surfactants, we denoted the central ring as cr1, the hydrophobic ester chain branch from C2 as long tail lt2, the polyethylene glycol (PEG) branch from C2 as short tail st3, the PEG at C3 as short tail st4, and the PEG at C4 as short tail st5 (Figure 1). We define that an interaction exists between a position in the PMO molecule and a position in the surfactant molecule if the distance between their centers is less than 7.5 Å. We determined the existence and time fraction of specific interactions as described in the supplemental information. In Figures 6 and S5, we display the conformer-specific PMO-surfactant interaction maps, showing the time fraction of the persistence of interactions between the

PMO modified nucleotides and surfactant side chains for each conformer I, II, and III of the 25-mer PMO and 30-mer PMO, as well as the total PMO-surfactant interactions maps for each of the 25-mer and 30-mers' conformers I, II, and III combined. Figures 6 and S5 show the results for Polysorbate 80 and Polysorbate 20, respectively. The first clear finding is that the hydrophobic long tails (lt2) of Polysorbate 80 and Polysorbate 20 interact with PMO residues more often than the more hydrophilic short tails (st3–st5), in both the individual PMO conformers and in the combined conformer representation at nearly all PMO residue positions.

Next, we examined the conformer-specific interaction patterns. For the 25-mer PMO conformer I, the most interactive parts of the molecule are the nucleotides at positions 10 (G),11 (T), 14 (T), and 15 (G). These bases interact with both Polysorbate 80 and Polysorbate 20 for longer than ∼35% of the time (persistent contacts), forming preferred, stronger interacting sites compared to other positions. Some other positions, such as 6 (G), 8 (A), and 21 (C), interact with Polysorbate 80 and Polysorbate 20 less frequently (>∼25% of the time) but still form persistent PMO-surfactant contacts. Positions 10 (G) and 11 (T) of the 25-mer PMO also interact ∼50% of the time with other portions of Polysorbate 80 (central ring cr1 and short tails st3–st5), forming other preferred, stronger interacting sites. Next, for the 25-mer PMO conformer II, positions 9 (A), 10 (G), and 12 (C) form persistent contacts (∼35% of the time) with both Polysorbate 80 and Polysorbate 20. Positions 22 (G) and 24 (T) interact with ∼35%–40% of the time with Polysorbate 80 (long tail lt2) and for ∼25% of the time with Polysorbate 20 (long tail lt2). Positions 6 (G), 7 (G), and 8 (A) interact with Polysorbate 20 about 30%–35% of the time, but only 5%–10% of the time with Polysorbate 80. Finally, for the 25-mer PMO conformer III, positions 1 (C), 2 (T), 14 (T), 15 (G), 19 (T), 23 (T), 24 (T), and 25 (G) interact >35% of the time with Polysorbate 20 (persistent contacts). Positions 15 (G), 17 (C), and 25 (G) strongly couple with Polysorbate 80, forming other preferred, stronger interacting sites with long 30%–40% interaction time. Additionally, positions 1 (C), 7 (G),10 (G), 18 (C), and 19 (T) in the 25-mer PMO conformer III form persistent contacts with the long tail lt2 of Polysorbate 80 (28%–33% of the time).

For the 30-mer PMO conformer I, the most interactive nucleotides are at positions 23 (A), 24 (C), and 25 (A). These bases interact with both surfactants Polysorbate 80 and Polysorbate 20 (long tail lt2 and short tail st4) for longer than ∼35% of the time (persistent contacts), forming preferred, strongly interacting sites. Other positions, such as 16 (A), 17 (G), and 28 (C), also form strong persistent contacts (>∼30% of the time) with long tail lt2. Position 15 (A) also interacts ∼40% of the time with other fragments of Polysorbate 20 (long tail lt2), forming another preferred, stronger interacting site. Next, for the 30-mer PMO conformer II, positions 14 (G) and 27 (C) show long interaction time (35%–50%) with the long tails lt2 of both surfactants. Positions 6 (T), 13 (A), 17 (G), and 29 (T) are occupied ∼30%–45% of the time by the long tail lt2 of Polysorbate 80, while positions 15 (A), 16 (A), and 28 (C) are occupied ∼30%–35% of the time by the long tail lt2 of Polysorbate 20. Finally, for the 30-mer PMO conformer III, positions 9 (C), 16 (A), 17 (G), 22 (T), and 23 (A) interact ∼50% of the time with the long tail lt2 of Polysorbate 80 (persistent contacts). Position 22 (T) interacts with the central ring cr1 and short tails st3, st4, and st5 of Polysorbate 80 ∼40% of the time, representing another preferred, stronger interaction. These same positions interact with the long tail lt2 of Polysorbate 20 for ∼35–40% of the time. Positions 3 (T), 10 (G), and 20 (A) strongly interact with Polysorbate 80, forming another preferred, stronger interacting site (30–40% interaction time), and positions 7 (T), 10 (G), 15 (A), and 21 (C) are occupied by the long tail lt2 of Polysorbate 20 for ∼30%–40% of the time.

Clearly, the folding patterns of individual conformers affect the position-specific strong interactions observed in the 25-mer and 30-mer PMOs. However, it is informative to take an overall view of the interactions of the PMOs with the individual surfactants. In the combined conformers' representation, nearly all PMO positions interacted most strongly with the long hydrophobic lt2 tail of both surfactants. The strongest interactions with Polysorbate 80 (Figures 6G and 6H) occurred at position 10 (G) for the 25-mer and at positions 16 (A), 17 (G), and 23 (A) for the 30-mer; in both cases, these strongest interactions were all at purine base positions. In contrast, for Polysorbate 20 interacting with PMOs (Figures S5G and S5H), positions 10 (G), 11 (T), and 14 (T) in the 25-mer and 14 (G), 15 (A), and 25 (C) in the 30-mer were most strongly interacting. The Polysorbate 20 strongly interacting sites showed no clear preference for purine bases, unlike Polysorbate 80. This may reflect the shorter hydrophobic tail of Polysorbate 20 (11 aliphatic C-atoms) compared to Polysorbate 80 (17 aliphatic C-atoms), with the latter surfactant lt2 tail seeking out the larger purine base faces for stronger hydrophobic interactions.

Next, we analyzed the intramolecular PMO interactions occurring in the 25-mer and 30-mer PMOs and compared these interactions for the PMO molecules in the absence and presence of Polysorbate 80 and Polysorbate 20. The results are displayed in Figures S6 and S7 for the 25-mer and 30-mer PMOs, respectively, showing interaction patterns (displayed as colored binary interaction maps for various nucleotide positions). The pixels near the main diagonal reflect near-adjacent position base stacking, whereas the pixels farther from the diagonal account for base pairing and chain folding features, both of which reflect the extent and specificity of non-local structure formation. For all three PMO conformers I–III of both the 25-mer (Figure S6) and 30-mer PMOs (Figure S7), with and without Polysorbate 80 and Polysorbate 20, the patterns of base stacking and base pairing do not change substantially in the complexes. This implies that PMO-surfactant interactions do not change the patterns of PMO intramolecular base pairing, base stacking, or folding and hence do not lead to significant changes in the secondary or tertiary structures of the 25-mer and 30-mer PMOs. This confirms our observations in Figures 5C and 5D (see also Table 1) and reiterates our finding from the CD spectra, namely, that PMOs are largely unaffected structurally and chirally by interactions with Polysorbate 80 or Polysorbate 20.

To summarize this section, the presence of Polysorbate 80 and Polysorbate 20 results in the formation of both weak and strong (preferred position-specific PMO-surfactant contact) interactions; yet, these strong PMO-surfactant interactions do not alter the secondary structure or significantly affect the tertiary structure of PMO molecules.

DISCUSSION

PMOs play an increasingly important role in the development of ASO-based approaches to drug discovery, which have led to the approval of several nucleic acid therapeutics for clinical use.[1] However, there is limited information on how PMOs interact with even the most widely used surfactant molecules. This represents a substantial gap in knowledge because surfactants are essential components of pharmaceutical formulations: they reduce interfacial tension, stabilize colloidal systems, and prevent aggregation or precipitation of active pharmaceutical ingredients (APIs), including oligonucleotides.[25] Surfactants also enhance wetting and solubility of hydrophobic domains, mitigate shear-induced degradation during processing, and limit adsorption to container surfaces.[26] Thus, understanding how PMOs interact with these excipients is critical for guiding formulation decisions that directly influence the stability, bioavailability, and overall viability of PMO-based therapeutics.

In our previous study,[14] we carried out a combined experimental and computational exploration of a range of phosphorodiamidate morpholino oligonucleotides, including the 25-mer PMO and the 30-mer PMO (Figure 1) analyzed in this study. This was the first systematic study of the solution structural, molecular, and thermodynamic properties of PMOs at the atomic level of structure detail. Molecular experiments *in silico* play an important role in advancing our understanding of the properties of *in vivo* biomolecules, including DNA, RNA, proteins, and RNA derivatives.[27,28] We found that PMO molecules form non-canonical, partially helical, extended, stable, and folded structures (on the sub-microsecond timescale) with a small radius of gyration (1.4–1.7 nm) and a low count of base pairs (3–6) and base stacks (6–9). The PMOs' structural stability is characterized by −35 to −50-kcal/mol of unfolding free energy. Because the PMO conformational dynamics highlighted the importance of the conformational ensemble view of PMO solution structures and properties,[14] here, we studied the properties of the three most important conformations of the 25-mer and 30-mer PMOs (principal solution conformers I–III; see Figures 4A and 4C). We aimed to explore the molecular properties and solution structures of these same key 25-mer and 30-mer PMO conformers I–III in the presence of the widely used nonionic surfactant molecules Polysorbate 80 and Polysorbate 20, at varying stoichiometries in surface tension and CD spectroscopy experiments, and at a 1:1 interaction stoichiometry computationally. Considering that PMO molecules are charge-neutral and Polysorbates are non-ionic, all interactions are expected to be primarily hydrophilic/hydrophobic in nature, with long-range electrostatic interactions playing a minor role. This represents one of the few examples in which hydrophilic/hydrophobic interactions, rather than electrostatics, are most predominant in a well-defined surfactant-biomolecule interaction.

Equilibrium surface tension was measured for varying concentrations of Polysorbate 80 and Polysorbate 20, both alone and in combination with PMOs. For surfactants alone (Figures 2B and 2C), CMC values were determined to be 0.02 mg/mL and 0.07 mg/mL for Polysorbate 80 and Polysorbate 20, respectively (Table S1). These values are similar to those previously reported in the literature.[23,24] Surface tension measurements were then performed in the presence of a fixed 50 mg/mL PMO concentration over the same range of Polysorbate 80 and Polysorbate 20 concentrations. Surface tension results from these studies do not overlay with those of the surfactant solutions alone but show remarkably distinct regions 2–5, very similar to the idealized protein-surfactant experimental diagram presented in Figure 2A,[20] indicating that the surfactants interact with PMO molecules in a well-defined way. Figures 2B and 2C show that, in both cases, an initial steep surface tension decrease (region 2) was observed that mimicked the results for surfactant alone. This was followed by an abrupt departure from the surfactant-only curve, forming a plateau (region 3), transitioning to a gradual drop (region 4), and leading to a final plateau (region 5) that again matched the surfactant-only surface tension values.

Overall, the features are the same for both surfactants, although some clear differences are observed. The region 2 to region 3 transition (CAC values, Table S1) was significantly delayed for Polysorbate 20 relative to Polysorbate 80, indicating a stronger interaction of Polysorbate 80 with PMO molecules. The CAC values for both surfactants followed a similar low-to-high order for the increasing PMO size series of 22-mer, 25-mer, and 30-mer PMOs. This may reflect the increasing PMO size giving rise to a slightly decreasing solution molarity for the surfactant to interact with at the constant 50 mg/mL concentration used in the surface tension measurements. Then, since the order of molar concentrations is 22-mer >25-mer >30-mer, this also corresponds to the order of initial surfactant concentrations (CAC values), from lower to higher, at which interactions with PMOs begin in the system. Finally, the calculated stoichiometry of interaction, *n*, the surfactant-to-PMO number ratio at the CMC (Table S1), was shown to be lower for Polysorbate 80 compared to Polysorbate 20. This again indicates a stronger interaction of Polysorbate 80 with PMOs. On average, a lower number of Polysorbate 80 molecules are bound per PMO molecule compared to Polysorbate 20s. This is undoubtedly due to the constraint of a larger size of Polysorbate 80, which leads to a lower stoichiometric ratio compared to the smaller Polysorbate 20. Behaviors similar to these PMO surface tension results have been reported for Polysorbate 80 or 20 titration-based experiments in protein systems, although the regions 3 and 4 deviations for the complex behavior from that of the pure polysorbate behavior are less well defined in the reported cases of BSA-Polysorbate 80[29] and BSA-Polysorbate 80 and Polysorbate 20 complexes.[30] In the latter case, and in other literature reports,[31] the results also clearly show that these mild Polysorbate 80 or Polysorbate 20 surfactants prevent or kinetically slow down the formation

of protein aggregates. Since proteins exhibit interactions with nonionic surfactants, as observed from experimental surface tension curves, similar to PMOs, and since both proteins and lipids contain hydrophobic functional groups with properties similar to the planar faces of the bases in PMOs, we would expect that these molecules might compete with PMOs for binding to Polysorbate 80 and Polysorbate 20 surfactants.

CD spectroscopy can reveal how surfactants interact with PMOs and whether they cause any disruptions in the folding patterns of the ensemble of solution conformers. Previously,[14] we found that the CD spectra of the 22-mer, 25-mer, and 30-mer PMOs were similar and resembled an A-type canonical RNA helical spectrum, with a dominant feature of right-handed chirality due to the interacting stacked bases evident in the PMOs' solution structures (Figures 5A and 5C). Since these three PMOs' CD spectra were so similar, in this study, we determined the CD spectra of only one representative PMO, the 30-mer, in the presence of either Polysorbate 80 or Polysorbate 20 (Figures 3A and 3B). At both higher and lower Polysorbate 80 concentrations of 0.2 and 0.02 mg/mL, the CD spectra of the complexes are nearly identical at all wavelengths to that of the 30-mer alone, with a similar result observed for Polysorbate 20 at the higher concentration (0.2 mg/mL). These data indicate that both surfactants interact with the 30-mer PMO in a way that causes no significant changes in the chiral properties of the folded solution conformers, either in the backbone (∼210–245 nm region) or in the bases (∼245–300 nm region). These CD results agree with other studies of these mild surfactants, including Polysorbate 80 and Polysorbate 20 interacting with proteins and antibodies.[32]

To provide interpretations of the surface tension and CD experiments in terms of an atomic-level view of the PMO-surfactant interactions, we carried out all-atom MD simulations for surfactants and then for their combinations with each of the three most prominent conformers I, II, and III, of both the 25-mer and 30-mer PMOs. As described previously, we chose only the 25-mer and 30-mer for MD simulations as representative structures, since the properties of the three PMOs were so similar.[14] As previously stated, we focused on simulating only 1:1 complexes of the surfactants with PMOs for several reasons: (1) we could estimate the 1:1 PMO-surfactant binding energies; (2) we could clearly understand the 1:1 binding pattern and PMO site preferences for the interacting surfactant molecules, without the complications arising from multiple surfactants competing for binding; and (3) simulating 2–3 or more surfactant molecules per PMO would require a prohibitive simulation time (this will be addressed in a separate study). Notwithstanding the practical difficulty of carrying out MD simulations on larger systems, there remains the interesting question of whether the presence of multiple surfactant molecules leads to synergistic stabilization of PMO structure or to competitive binding. Our results indicate that, while PMO-surfactant interactions are moderately strong (several tens of kcal/mol), they exhibit demonstrable but limited specificity, and surfactants do not alter the PMO secondary or tertiary structures. This suggests that the presence of several surfactant molecules per PMO at high surfactant concentrations is expected to provide additional stabilization of PMO structure, so long as the PMO surface coverage is not full, but will likely lead to competitive binding at high surfactant concentration when the number of surfactants per PMO exceeds what the PMO molecular surface can accommodate.

Through simulations, we found the following: (1) Polysorbate 80 and Polysorbate 20 alone exhibit dynamic structure behavior, rapidly transitioning between a continuum of extended and collapsed conformations; (2) Polysorbate 80 and Polysorbate 20 interact with PMOs, either weakly or more strongly, mostly at the PMO surface, forming slightly more extended surfactant structures with a variety of different PMO-surfactant interfaces; these interactions help prevent the PMO from unfolding and potentially aggregating[16] (in rare cases, we observed that PMO-surfactant interactions facilitate PMOs' partial unfolding and subsequent refolding into more compact structures); (3) the PMO-surfactant interactions do not perturb the PMO secondary-structure base-pairing and base-stacking arrangements but slightly enlarge the PMO tertiary structure (size); (4) surfactant molecules partially shield PMO molecules in the complex, lowering their *SASA* values, thereby making PMO conformations less exposed to solvent (water) and, hence, less prone to interact intermolecularly; (5) strong PMO-surfactant interactions are characterized by −60 to −80 kcal/mol interaction energy (for the 25-mer PMO) and −50 to −70 kcal/mol energy (for the 30-mer PMO); 6) there are preferred, stronger surfactant interaction sites in PMO structures that involve positions 10 (G), 14 (T), and 15 (G) in the 25-mer PMO and positions 14 (G), 15 (A), 16 (A), 17 (G), 22 (T), 23 (A), 24 (C), and 25 (A) in the 30-mer PMO, which involve atomic groups in the long hydrophobic tail (lt2) and also the three shorter, more hydrophilic polyoxyethylene tails (st3–st5) in Polysorbate 80 and Polysorbate 20. In the remaining part of this paper, we discuss these findings in more detail.

Polysorbate 80 and Polysorbate 20 strongly interact with PMOs

The experiments detailing the significant effect of increasing Polysorbate 80 and Polysorbate 20 concentrations on the measured surface tension of PMO solutions, compared to surfactant-only solutions (Figure 2), strongly suggested that significant interactions exist between PMOs and the surfactant molecules. We found that both Polysorbate 80 and Polysorbate 20 in solution exhibit dynamic structural changes, rapidly transitioning back and forth between extended and collapsed conformations (Figure S2). The extended and collapsed structures for Polysorbate 80 (R_g ranging between 1.0 and 0.6 nm, respectively, Table S5), forming a continuum of physical properties (Figure S2), and the low level of hydrogen bonds (Table S5) point to the lack of significant internal secondary structure in both extended and collapsed conformations for both surfactants. Analysis of surfactant molecules shows that their size (R_g, L, and W) increases and *SASA* decreases (Table S5) in the presence of PMO. Dynamic properties of Polysorbate 80 and Polysorbate 20 are almost the same, except for *SASA*. For all cases, with and without PMO complexation, Polysorbate 80 has slightly higher values of

SASA, in line with its slightly greater size compared to Polysorbate 20. These features of the surfactants' solution structures, properties, and dynamic behavior are more detailed than those provided for these molecules in a few previous studies,[33–36] with the first study[33] providing a specific point of comparison, an R_g = 0.75 nm value for Polysorbate 80, nearly identical to our R_g = 0.77 nm value (Table S5). Next, we studied the interaction of Polysorbate 80 and Polysorbate 20 with the three most populated solution conformers, I – III, for 25-mer and 30-mer PMOs. The interactions were transient, ranging from weak ones at the PMO surface only, formed by more extended bound surfactant structures with differing PMO-surfactant interfaces (Figure 4), to stronger, longer-lived interactions, formed by surfactant molecules sometimes penetrating the PMO folded structures (Figure 4B conformer I; Figure 4D conformers II and III).

Polysorbate 80 and Polysorbate 20 do not alter PMOs' secondary and tertiary structures

For almost all conformations of 25-mer and 30-mer PMOs we studied, PMO-Polysorbate 80 and Polysorbate 20 complex interactions do not perturb the PMO secondary structure base-pairing and base-stacking arrangements, but they do slightly enlarge the PMO tertiary structure. For the 25-mer conformers I, II, and III, R_g for PMO within the Polysorbate 80 complex increases by 6.3%, 8.3%, and 6.7%, respectively, compared to PMO alone in solution, while the R_g increases for the Polysorbate 20 complex tend to be smaller, at 5.6%, 8.3%, and 3.0%, respectively, compared to PMO alone. For the 30-mer conformers I and II, similar increases were observed in R_g for PMO within the complexes compared to PMO alone: 3.5% and 3.5% for Polysorbate 80, respectively, and 1.4% and 4.9% for Polysorbate 20, respectively. For the more extended 30-mer conformer III, the R_g decreased for the PMO within the Polysorbate 80 complex compared to PMO alone, a −2.6% change, while for the Polysorbate 20 complex, the decrease was very small, a −0.7% change. This different behavior of the complex for the 30-mer conformer III agrees visually with the snapshots of the PMO and the Polysorbate 80 complex shown in Figures 4C and 4D, where the complex appears overall smaller than the PMO alone.

PMO-surfactant interactions lead to increased PMO solubility

The *SASA* values for PMO conformers in both 25-mer and 30-mer PMOs free in solution decrease significantly for the PMOs within the complexes (Table 1), indicating that the interacting surfactant molecules partially shield the PMO interacting surface from solvent, thereby making the PMO conformations less prone to forming intermolecular complexes. Beginning at PMO concentrations slightly above those used in the MD simulations here (50 mg/mL), evidence for intermolecular interactions was observed in our previous study in the 75–100 mg/mL PMO range, from both experimental viscosity and MD simulation studies.[14] However, when the $SASA_{tot}$ values of the entire surfactant-PMO complexes were determined (including the surfactant), they were found to be significantly larger than those for the isolated PMO molecules (Table 1). These $SASA_{tot}$ values indicate increased exposure of the complexes to solvent compared to the isolated PMO molecules and, hence, increased solubility, which helps prevent the PMOs from aggregating at higher concentrations, as we noted in our previous study.[14] This characterization of surfactant interaction between Polysorbate 80 or Polysorbate 20 and conformers of the 25-mer and 30-mer PMOs is the first study of its kind for this class of RNA mimic oligonucleotides. Therefore, the complete lack of published studies on a similar system with which to compare these results leads us to examine studies of Polysorbate 80 and Polysorbate 20 interacting with proteins. This is especially relevant given the resemblance of our surface tension results (Figure 2) to those of surfactant-protein complexes[37] and the protein-like structure features of PMO solution structures, i.e., lack of canonical nucleic acid secondary structure, lack of charged residues, a somewhat folded conformation that minimizes solvent exposure to less hydrophilic regions and maximizes solvent exposure to more hydrophilic regions (base edges).

Polysorbate 80 and Polysorbate 20 surfactants can exhibit molecular chaperone-like activity

Various surfactants interact with proteins in a range of ways,[38] including disrupting native structures for strong surfactants[39] and helping maintain or refold protein structure for mild surfactants.[32,40] Polysorbate 80 and Polysorbate 20 fall into the latter class in their interactions with proteins. A number of both experimental[41,42] and computational[36] studies of Polysorbate 80-protein complexes demonstrate that there is a significant but overall stabilizing effect on the protein's native structures. In our study, we observed that Polysorbate 80 served in a "chaperone-like" manner to refold the PMOs (see Video S4). The interaction maps of these three different conformation states—initial folded, transiently extended, and final refolded—suggested that a more energetically favorable final refolded state was created, exhibiting more base-stacking interactions, as well as longer persistence of base-pairing, when compared to the initial folded and transiently extended states (Figures 7B, 7D and 7F).

PMO-surfactant complexes have significant binding energies

Strong PMO-surfactant interactions occur rapidly in our MD simulations for both Polysorbate 80 and Polysorbate 20 complexes (by 1 μs, Figures 5E and 5F; Figure S4) and are finally characterized by −60 to −80 kcal/mol interaction energy for the 25-mer PMO and −50 to −70 kcal/mol energy for the 30-mer PMO (Table S6). Interestingly, while the overall interaction energy decreases during most of the simulation period, the measured R_g and *SASA* for the PMO-surfactant complexes reach their final values as soon as the complex initially forms and remain at these values during the 1 μs simulation (Figure 5). These kinetic behaviors suggest that the overall shape and solvent accessibility of the complex are established as soon as the complex initially forms but that the final interaction energy levels are reached with slower kinetics as transient complexes rapidly form, dissociate, rearrange, and reform, with more localized rapid surfactant dynamics and rearrangements within more stable complexes occurring in order to achieve their final energy-minimized conformations.

A number of physicochemical factors could influence the energetics of PMO-surfactant interactions; these include pH, temperature, and

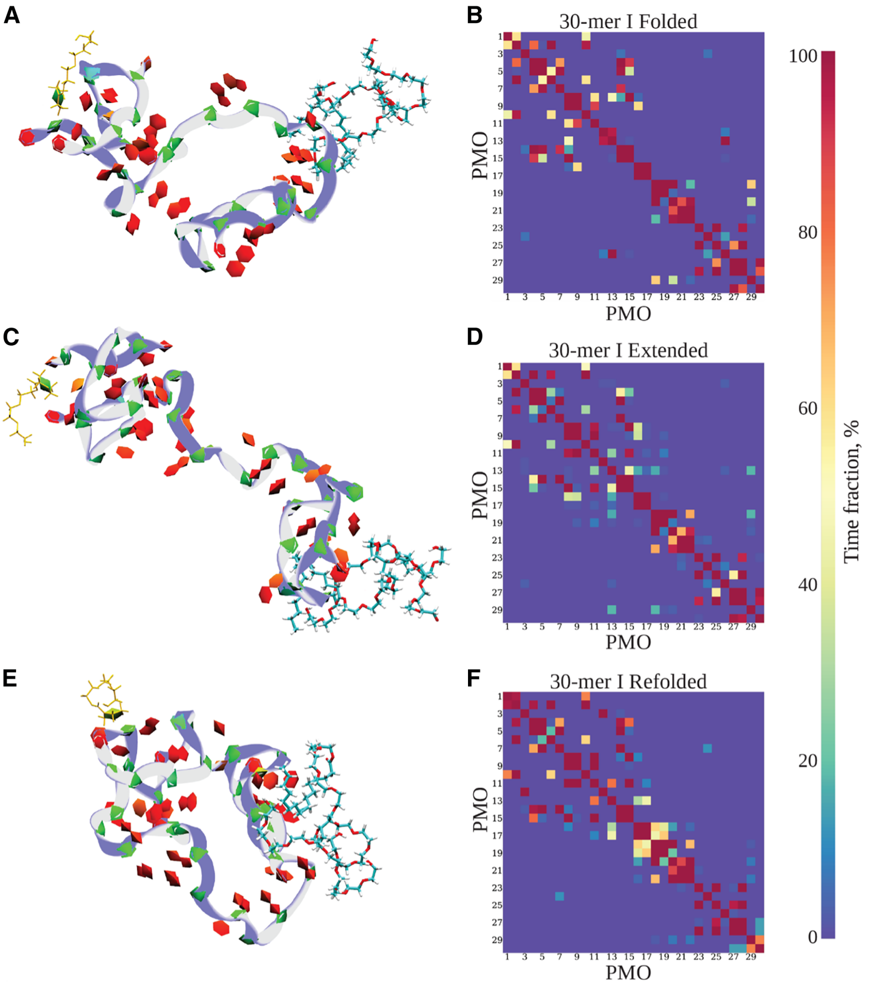

Figure 7. PMO-Polysorbate 80 interaction maps for 30-mer conformer I in folded, extended, and refolded states

Displayed are structure snapshots of PMO 30-mer conformer I interacting with Polysorbate 80 (left) and the corresponding maps showing the time fraction (percentage) of interactions between bases in PMO 30-mer conformer I (right). The PMO-Polysorbate 80 complex started in a folded state (0–400 ns) (A), progressed to an extended state (400–560 ns) (C), resumed to a new folded state (not shown) (560–600 ns), then another extended state (not shown) (600–630 ns), and equilibrated to a refolded state (630–1000 ns) (E) during the 1-μs MD simulation run. The PMO structure representation is the same as in Figure 4. The PMO-PMO interaction maps corresponding to the structures shown in (A), (C), and (E) are presented to the right in (B), (D), and (F), respectively. The time-fraction vertical scale is displayed on the right.

ionic strength. Although the hydrophobic nature of the interaction is sequence-independent, the extent of binding correlates with the local hydrophobic surface area exposed by the sequence-dependent PMO conformation, and this could be influenced by the solution environment through pH, temperature, or ionic strength. Although our simulations did not explicitly probe pH effects, the physiochemical functional group properties of PMOs suggest minimal protonation within the physiologically relevant range (pH 5.5–7.5). Consequently, only subtle shifts in hydrogen bonding or base stacking would be expected. With respect to temperature variation, an increase in temperature can lead to a reduction in the hydration of the surfactant head group.[43] For some oxyethylene containing nonionic surfactants, this can result in a higher degree of dehydration around the head group region, which in turn reduces the head group size and increases its hydrophobicity.[44] This process can lead to closer molecular packing. As a result, the hydrophobic interactions between the surfactant alkyl chains and PMO surfaces might transiently become more favorable. However, the negative binding energy values obtained for all PMO systems interacting with the surfactants Polysorbate 20 and Polysorbate 80 imply exothermic binding, which means that increasing (or decreasing) temperature should result in weakening (or strengthening) of non-specific interactions between PMOs and the nonionic surfactants Polysorbate 20 and Polysorbate 80. Ionic strength can affect the stability and aggregation of proteins in formulations containing Polysorbate 80.[45] In the context of PMO–surfactant systems, the effect of ionic strength cannot be determined with certainty given our data. However, PMOs are charge-neutral, so high-salt conditions are unlikely to directly alter their structure and properties. Instead, ionic strength might influence surfactant behavior, potentially by modulating the bulk solution electronic environment via a change in dielectric constant, through which their approach to binding would be affected.

There exist strong, longer-lived, preferential surfactant interaction sites in PMO structures

While all PMO positions for all conformers interact weakly and briefly with surfactants to some extent, there are preferred, stronger, longer-lived surfactant binding sites in the PMO structures. We find that the long (hydrophobic) tails of Polysorbate 80 and Polysorbate 20 (denoted lt2 in Figures 6 and S5) interact with PMOs significantly more often than the three shorter, more hydrophilic tails (st3, st4, and st5 in Figures 6 and S5). Here, we emphasize only the preferred, stronger interacting sites. These involve positions 10 (G), 14 (T), and 15 (G) in the 25-mer PMO and positions 14 (G), 15 (A), 16 (A), 17 (G) and 22 (T), 23 (A), 24 (C), and 25 (A) in the 30-mer PMO interacting with atomic groups in the long hydrophobic and shorter, more hydrophilic tails found in Polysorbate 80 and Polysorbate 20. Among the PMO positions where stronger interacting sites occur,

there exists a preponderance of purine bases. These larger 2-ring system planar bases, whose hydrophobic faces interact strongly with the surfactants' long hydrophobic tails, represent long-lived interaction sites. These preferential hydrophobic interactions are analogous to certain hydrophobic protein residues interacting with surfactant molecules in complexes, such as in the experimental Nuclear Magnetic Resonance (NMR) examination of Polysorbate 80 and Polysorbate 20 interactions with an antibody and antibody fragments.[32] Protein amino acid residue interaction preferences with surfactants may target the same residues that have been identified as aggregation-prone regions in purely protein solutions that are responsible for aggregation at higher concentrations.[42] Software has been developed that describes how energetically preferential regions of proteins interact, leading to aggregation.[46,47]

While a wide ranging, general discussion of different surfactant classes is beyond the scope of this focused study, it should be noted that anionic and cationic detergents,[48–50] as well as other nonionic surfactants[51–57] have been used in pharmaceutical research. Notably, neutral surfactants such as BigCHAP and Deoxy-BigCHAP have been reported to substantially improve PMO exon-skipping efficiency *in vitro* and in mouse models, enhancing delivery by up to 7-fold while maintaining low cytotoxicity.[57] Additionally, PMOs, being uncharged, were found to electrophorese in Sodium Dodecyl Sulfate-containing gels (SDS).[48] Therefore, SDS interacts with PMOs in the gel matrix much as it does with proteins, causing electrophoretic mobility. This study can be explained by, and supports, our view that the strongest interaction features of Polysorbate 80 or 20 molecules involve the hydrophobic tail, which we believe interacts preferentially with the faces of the large purine bases at specific sequence locations identified in our MD simulation-based interaction patterns. The same base features of PMOs in the gel electrophoresis study must interact with the 12-carbon hydrophobic alkyl chain of the anionic SDS detergent in the gel in order for the uncharged PMOs to exhibit mobility in the electrophoresis experiment.

In general, detailed structural information about biomolecules and their derivatives comes from X-ray crystallography or NMR experiments. However, PMO molecules are too labile to form crystalline substances and too complex (∼880–1300 atoms) for NMR experiments. However, PMOs are small-size molecules for cryo-EM techniques. Small-angle X-ray scattering (SAXS) measurements enable researchers to obtain information about interatomic distances, which can then be used to estimate the ensemble-average shape and size of PMO molecules but not their detailed atomic arrangements. Importantly, none of these methods offers a conformational ensemble view of biomolecules in aqueous solution, which is critically important for therapeutic PMO molecules. In our prior studies of PMO molecules[14] and PPMO (Peptide-conjugated phosphorodiamidate morpholino oligonucleotides) molecules,[58] we combined experimental CD and viscosity measurements with all-atom MD simulations and with machine learning-based modeling to generate new knowledge about PMO and PPMO structure. In those studies, we showed, for the first time, that an ensemble of PMO and PPMO structures exists in solution, rather than a single or several selected structures; these ensembles define the molecular, hydrodynamic, and thermodynamic properties of individual PMOs and PPMOs.[14,58]

In this study, we showed that ensembles of PMOs interacting with the mild nonionic surfactants Polysorbate 80 and Polysorbate 20 behave in a protein-like manner, based on both the experimental surface tension and CD data and the results from MD simulations. These simulations reveal that while surfactants interact with PMOs with favorable interaction energy, they only slightly alter the individual folded PMO conformations. The base pairs and base stacks do not change significantly with the addition of surfactant, in agreement with the CD results showing no alteration of the chiral properties of the complexes relative to those of the isolated PMOs. The PMO-surfactant complexes have increased interaction with solvent, and the resulting increased solubility relative to PMOs alone is an indication that surfactant interactions in the complexes can help prevent potential aggregation of PMOs in solution at higher concentrations. The hydrophobic tails of both Polysorbate 80 and Polysorbate 20 interact more strongly with PMOs at nearly all positions in the ensemble of structures, but in specific conformers there are PMO positions that have much stronger interactions with the surfactant than other positions.

Overall, the intensive properties of PMO-surfactant complexes are similar for both surfactants. These include: (1) CD spectral signatures that are similar for both the Polysorbate 80:PMO and Polysorbate 20:PMO complexes, both in the PMO backbone and in the bases (molecular chirality); and (2) patterns of PMO intramolecular base stacking and base pairing (secondary structure propensity). However, not unexpectedly, some small quantitative differences are observed in the extensive properties of PMO-surfactant complexes, due to the slightly larger size of Polysorbate 80 compared to Polysorbate 20. These include: (1) a small (∼1–2-kcal/mol) interaction energy difference, with the Polysorbate 80:PMO complexes > Polysorbate 20: PMO complexes (PMO-surfactant interactions); (2) slightly higher values of *SASA* for the Polysorbate 80:PMO complexes compared to the Polysorbate 20: PMO complexes (PMO solubility); and (3) a slightly smaller surfactant-to-PMO number ratio at the CMC (interaction stoichiometry) for Polysorbate 80. The slightly stronger PMO-surfactant interaction energy and higher SASA values for the Polysorbate 80:PMO complexes explain, for example, the differences in the transition from region 2 to region 3 and region 4 to region 5 in the surface tension profiles compared to the Polysorbate 20:PMO complexes.

To conclude, the results obtained here are the first of their kind. The MD simulation-based studies explain the experimental CD results of the 1:1 PMO-surfactant complex by showing that the nonionic surfactants Polysorbate 80 and Polysorbate 20 help stabilize the solution conformer structures of the uncharged PMO ensemble. Also, completely novel are the results of the examination of both the energetics and interaction patterns of 1:1 PMO-Polysorbate 80 and

Polysorbate 20 complexes. The interaction patterns identify specific preferred PMO residues that interact preferentially with the surfactants' hydrophobic tails, but these residues also interact with the other three surfactant arms. Based on the data shown in this study, it is concluded that the secondary and tertiary structure of PMOs remain unaffected by the presence of surfactants, being mostly stabilized; in a few cases, however, we observed that the provide a "chaperone-like" function, helping extended conformers to refold. It would be interesting in future studies to explore PMO-surfactant interactions in the presence of more than one non-ionic surfactant, such as Polysorbate 80 and Polysorbate 20. Another valuable physico-chemical aspect of this work is that hydrophilic and hydrophobic interactions can be studied separately, which contributes to a better understanding of non-ionic intermolecular forces. Building on the findings from this study, the behavior of PMOs in the presence of Polysorbate 80 and Polysorbate 20 offers key insights for related systems. In particular, PPMOs, which share the same morpholino backbone and nucleobase composition, are likely to display similar behavior to PMOs; therefore, this study can help guide formulation strategies for PPMOs. More broadly, while other ASO chemistries vary in charge, backbone, or hydrophobicity, these general mechanisms—hydrophobic surface recognition, modulation of interfacial behavior, and surfactant-mediated structure stabilization—might offer a conceptual framework to optimize excipient interactions across a wider range of oligonucleotide therapeutics.

MATERIALS AND METHODS

Surface tension studies

We prepared a 50 mg/mL stock solution of PMO in PBS (using 5.5 g in 100 mL, with 10% excess to compensate for moisture in the PMO drug substance). This solution was split into 25 mL quarters and spiked one 25 mL quarter with concentrated Polysorbate 20 and another 25 mL quarter with Polysorbate 80, sourced from Sigma-Aldrich, each to a surfactant concentration of 20 mg/mL. The stock surfactant solutions were then dosed incrementally into the remaining quarters, which contained only 50 mg/mL PMO and initially no surfactant. In this manner, the PMO concentration was held fixed throughout at 50 mg/mL. All experiments were performed in duplicate. All surface tension work was carried out using the Wilhelmy plate technique on a Kruss K100 Tensiometer with automated dosing at Augustine Scientific, Newbury OH. We used a minimum equilibration time of 2 min after each dose-and-stir concentration augmentation before each measurement. We also required a ±0.02 mN/m standard deviation over ten stationary measurements to determine the surface tension at that current PMO concentration before moving on to the next measurement. CMC surface tension measurements were performed by titrating the PMO solutions with 20 mg/mL Polysorbate 80 or Polysorbate 20 stock solutions into PMO solutions without surfactant. All solutions had a fixed PMO concentration of 50 mg/mL, and all measurements were performed at 25°C.

CD spectroscopy

CD measurements were carried out at 25°C using a Chirascan Q100 Circular Dichroism Spectrometer with Pro-Data Viewer v.4.7.0.194 data analysis software (KBI Biopharma, Louisville, CO). The concentration of the sample was adjusted based on Beer's law to maintain an absorbance signal of ∼1.2 AU. It was noted that the cell pathlength does not have a significant impact on the CD spectrum noise level; therefore, a 1-cm pathlength cell was used for testing with a target PMO concentration of 0.04–0.06 mg/mL. The PMO solutions were prepared by spiking concentrated 10% solutions of Polysorbate 20 (Thermo Fisher Scientific, cat. 28328) or Polysorbate 80 (Thermo Fisher Scientific, cat. 28320) into a stock 50 mg/mL PMO solution in PBS buffer. Solutions were further diluted to the target 0.04–0.06 mg/mL PMO concentration with PBS buffer. The raw CD spectra were buffer subtracted, baseline-corrected, and normalized to the mean residue molar ellipticity.

Construction of PMO structures

The atomic models of PMOs, comprising 25 nucleobases (25-mer) and 30 nucleobases (30-mer), were constructed using the VMD package.[59] The atomic coordinates of the 6-member morpholino ring in the "chair" conformation were obtained by Caleman et al.[60] To obtain "morpholino nucleotides," i.e., the canonical nucleotides with the 5-member ribose ring substituted by the 6-member morpholino ring, we attached guanine (G), cytosine (C), thymine (T), and adenine (A) nucleobases to the morpholino ring. The connection between the morpholino ring and any nucleobase is through the C1′−N1 covalent bond.[14] The linker (PMO) oligomer backbone was formed by connecting "morpholino nucleotides" through phosphorodiamidate groups. The morpholino TEG piperazine linker was attached to the 5′-end of each sequence through the phosphorodiamidate group.[14]

MD simulations

Atomic partial charges and force field parameters for PMO were derived in our previous work.[14] Force field development for the surfactant molecules Polysorbate 20 and Polysorbate 80 is presented in the supplemental information (SI). The all-atom MD simulations were carried out as described in the previous studies.[61,62] *System preparation:* Each of the 25-mer and 30-mer PMOs, combined with either Polysorbate 80 or Polysorbate 20, was solvated in an octahedron water box. The water box contains one PMO molecule and one surfactant molecule, along with: ∼11,500 water molecules (400 nm^3 volume; 34.6 mg/mL PMO concentration) for 25-mer PMO and Polysorbate 20, ∼14,300 water molecules (500 nm^3 volume; 27 mg/mL PMO concentration) for 25-mer PMO and Polysorbate 80, ∼13,600 water molecules (∼475 nm^3 volume; 34 mg/mL PMO concentration) for 30-mer PMO and Polysorbate 20, and ∼15,000 water molecules (515 nm^3 volume; 31 mg/mL PMO concentration) for 30-mer PMO and Polysorbate 80. These simulation setups correspond to 1:1 PMO:surfactant stoichiometries, with ∼3 mM surfactant concentration and approximately 50 mg/mL PMO concentration, consistent with the surface tension experiment. In our previous study of PMO molecules,[14] we showed that the CD spectra obtained for the uncharged PMO molecules reconstituted and diluted in water and in PBS were very

similar, which implies that the ensembles of PMO conformations in aqueous solution and in PBS buffer are similar. In the current study, we describe the PMO molecules in combination with the mild surfactants Polysorbate 20 and Polysorbate 80 in pure aqueous solution. *Energy minimization:* Energy minimization was performed first using the steepest descent algorithm[63] (over 10,000 steps) and then using the conjugate gradient method (over 5000 steps). A 50 kcal/mol energy restraint was applied to all solute atoms during this step. *Heating:* Each system was heated at constant volume from 0 to 300 K over a 50-ps time frame. *Equilibration:* Equilibration for each complex was achieved after 100 ps of restrained MD simulations, with all solute atoms constrained by a 0.05 kcal/mol energy restraint. This was followed by a 1-ns equilibration of each complex at constant pressure and 300 K, with all solute atoms constrained by a 1.0 kcal/mol energy restraint to allow the water density to relax. *Equilibrium simulations:* Unrestrained 1-μs MD simulation runs in water at T = 300 K were carried out for each PMO-surfactant system using the CUDA version of pmemd[64] in the GPU-accelerated[63,65] AMBER 20 package.[66] The following quantities were analyzed, as described in the supplemental information (SI): for PMOs $-R_g$, N_{bp} and N_{bs}, $SASA$, and the $RMSD$ (with respect to the initial structure taken as a reference state); for surfactants – R_g, n_{hb}, $SASA$, $RMSD$, L, and W.

Thermodynamic state functions

The energy (ΔE) for PMO-surfactant interaction was determined for each conformer observed in the equilibrium MD simulations of the 25-mer and 30-mer PMOs. We used the molecular mechanics/generalized Born surface area (MM/GBSA) method,[67] implemented in the MMPBSA.py program,[68] to analyze many thousands of conformations of the 25-mer and 30-mer PMOs, along with Polysorbate 80 and Polysorbate 20. The energy of a state is given by $E = E_{int}+E_{el}+E_{vdW} + E_p+E_{np}$, where E_{int} includes the bond length potential, bond angle potential, and dihedral angle potential; E_{el} is the electrostatic interaction potential; and E_{vdW} is the van der Waals interaction potential. The last two terms, E_p and E_{np}, are the polar and non-polar contributions to the solvation energy $E_{solv} = E_p+E_{np}$. Here, E_p is obtained using the GB model, and E_{np} is calculated from $SASA$. The energy changes, $\Delta E_j = E_j$-E_0 were estimated by taking the difference between the energy of an initial (reference) state, E_0, and that of the j-th conformation, E_j.

Theoretical reconstruction of CD spectra

The procedure is described in detail in our prior study.[14] Briefly, for each i-th structure type, $i = 1, 2, \ldots, N$- (where N is the total number of structures), a CD profile, $\theta_i(\lambda)$, is calculated using the matrix method.[69–71] This methodology is implemented in DichroCalc.[72] The average theoretical profile, $\Theta_{th}(\lambda)$, is constructed using a weighted superposition: $\Theta_{th}(\lambda) = \sum_i w_i\theta_i(\lambda)$, where w_i is the statistical weight for the i-th structure type ($\sum_i w_i = 1$). We used the mean squared error (MSE) as a penalty function, with population weights $w_1,w_2,\ldots,w_N$ as regression coefficients, to identify the primary (highest-weights) solution structures for the 22-mer, 25-mer, and 30-mer PMOs that best fit their experimental CD profiles.

DATA AND CODE AVAILABILITY

All data are available from the corresponding authors upon reasonable request and are included in the main text and supplemental material.

ACKNOWLEDGMENTS

We thank Dr. Christopher Rulison (Augustine Scientific) for his support in obtaining surface tension data and Nikki Machalek (KBI Biopharma) for support with the circular dichroism data. This work was conducted under a Sponsored Research Agreement between Sarepta Therapeutics and the University of Massachusetts, Lowell.

AUTHOR CONTRIBUTIONS

E.K.: Formal analysis, methodology, visualization, investigation, writing − original draft. D.P.: Formal analysis, investigation, visualization, writing − original draft. W.D.: Conceptualization, formal analysis, investigation, methodology, supervision. K.A.M.: Conceptualization, formal analysis, investigation, methodology, supervision, validation, visualization, writing − original draft. A.C.: Conceptualization, formal analysis, investigation, methodology, supervision, validation, visualization, writing − original draft. V.B.: Conceptualization, formal analysis, investigation, methodology, supervision, validation, visualization, writing − original draft.

DECLARATION OF INTERESTS

D.P. and A.C. are employees of Sarepta Therapeutics Inc. and own stock/options in the company. W.D. was an employee of Sarepta Therapeutics during preparation of this manuscript.

SUPPLEMENTAL INFORMATION

Supplemental information can be found online at https://doi.org/10.1016/j.omtn.2026.102845.

REFERENCES

1. Kulkarni, J.A., Witzigmann, D., Thomson, S.B., Chen, S., Leavitt, B.R., Cullis, P.R., and van der Meel, R. (2021). The current landscape of nucleic acid therapeutics. Nat. Nanotechnol. *16*, 630–643. https://doi.org/10.1038/s41565-021-00898-0.
2. Egli, M., and Manoharan, M. (2023). Chemistry, structure and function of approved oligonucleotide therapeutics. Nucleic Acids Res. *51*, 2529–2573. https://doi.org/10.1093/nar/gkad067.
3. Rinaldi, C., and Wood, M.J.A. (2018). Antisense oligonucleotides: The next frontier for treatment of neurological disorders. Nat. Rev. Neurol. *14*, 9–21. https://doi.org/10.1038/nrneurol.2017.148.
4. Lauffer, M.C., van Roon-Mom, W., and Aartsma-Rus, A.; N = 1 Collaborative (2024). Possibilities and limitations of antisense oligonucleotide therapies for the treatment of monogenic disorders. Commun. Med. *4*, 6. https://doi.org/10.1038/s43856-023-00419-1.
5. FDA (2021). Drug Approval Package: AMONDYS 45 (FDA).
6. FDA (2019). Drug Approval Package: Vyondys 53 (golodirsen) (FDA).
7. FDA (2016). Drug Approval Package: Exondys 51 Injection (eteplirsen) (FDA).
8. Warfield, K.L., Swenson, D.L., Olinger, G.G., Nichols, D.K., Pratt, W.D., Blouch, R., Stein, D.A., Aman, M.J., Iversen, P.L., and Bavari, S. (2006). Gene-specific countermeasures against Ebola virus based on antisense phosphorodiamidate morpholino oligomers. PLoS Pathog. *2*, e1. https://doi.org/10.1371/journal.ppat.0020001.
9. Iversen, P.L., Warren, T.K., Wells, J.B., Garza, N.L., Mourich, D.V., Welch, L.S., Panchal, R.G., and Bavari, S. (2012). Discovery and early development of AVI-7537 and AVI-7288 for the treatment of Ebola virus and Marburg virus infections. Viruses *4*, 2806–2830. https://doi.org/10.3390/v4112806.
10. Howard, J.J., Sturge, C.R., Moustafa, D.A., Daly, S.M., Marshall-Batty, K.R., Felder, C.F., Zamora, D., Yabe-Gill, M., Labandeira-Rey, M., Bailey, S.M., et al. (2017). Inhibition of Pseudomonas aeruginosa by Peptide-Conjugated Phosphorodiamidate Morpholino Oligomers. Antimicrob. Agents Chemother. *61*, e01938-16. https://doi.org/10.1128/aac.01938-16.
11. Warren, T.K., Shurtleff, A.C., and Bavari, S. (2012). Advanced morpholino oligomers: A novel approach to antiviral therapy. Antiviral Res. *94*, 80–88. https://doi.org/10.1016/j.antiviral.2012.02.004.

12. Nan, Y., and Zhang, Y.J. (2018). Antisense phosphorodiamidate morpholino oligomers as novel antiviral compounds. Front. Microbiol. *9*, 750. https://doi.org/10.3389/fmicb.2018.00750.

13. Das, U., Kundu, J., Shaw, P., Bose, C., Ghosh, A., Gupta, S., Sarkar, S., Bhadra, J., and Sinha, S. (2023). Self-transfecting GMO-PMO chimera targeting Nanog enable gene silencing in vitro and suppresses tumor growth in 4T1 allografts in mouse. Mol. Ther. Nucleic Acids *32*, 203–228. https://doi.org/10.1016/j.omtn.2023.03.011.

14. Maksudov, F., Kliuchnikov, E., Pierson, D., Ujwal, M.L., Marx, K.A., Chanda, A., and Barsegov, V. (2023). Therapeutic phosphorodiamidate morpholino oligonucleotides: Physical properties, solution structures, and folding thermodynamics. Mol. Ther. Nucleic Acids *31*, 631–647. https://doi.org/10.1016/j.omtn.2023.02.007.

15. Israelachvili, J.N. (2011). Intermolecular and Surface Forces (Academic Press).

16. H. Moulton, and J. Moulton, eds. (2017). Morpholino Oligomers (New York, NY: Humana).

17. Khan, T.A., Mahler, H.C., and Kishore, R.S.K. (2015). Key interactions of surfactants in therapeutic protein formulations: A review. Eur. J. Pharm. Biopharm. *97*, 60–67. https://doi.org/10.1016/j.ejpb.2015.09.016.

18. Koulov, A.V., and Singh, S.K. (2023). Surfactants in Biopharmaceutical Development (Academic Press).

19. Sen, D., and Gilbert, W. (1988). Formation of parallel four-stranded complexes by guanine-rich motifs in DNA and its implications for meiosis. Nature *334*, 364–366. https://doi.org/10.1038/334364a0.

20. Lee, H.J., McAuley, A., Schilke, K.F., and McGuire, J. (2011). Molecular origins of surfactant-mediated stabilization of protein drugs. Adv. Drug Deliv. Rev. *63*, 1160–1171. https://doi.org/10.1016/j.addr.2011.06.015.

21. Gunning, P.A., Mackie, A.R., Gunning, A.P., Woodward, N.C., Wilde, P.J., and Morris, V.J. (2004). Effect of surfactant type on surfactant-protein interactions at the air-water interface. Biomacromolecules *5*, 984–991. https://doi.org/10.1021/bm0344957.

22. Arsiccio, A., McCarty, J., Pisano, R., and Shea, J.E. (2018). Effect of Surfactants on Surface-Induced Denaturation of Proteins: Evidence of an Orientation-Dependent Mechanism. J. Phys. Chem. B *122*, 11390–11399. https://doi.org/10.1021/acs.jpcb.8b07368.

23. Mittal, K.L. (1972). Determination of CMC of polysorbate 20 in aqueous solution by surface tension method. J. Pharm. Sci. *61*, 1334–1335. https://doi.org/10.1002/jps.2600610842.

24. Patist, A., Bhagwat, S.S., Penfield, K.W., Aikens, P., and Shah, D.O. (2000). On the measurement of critical micelle concentrations of pure and technical-grade nonionic surfactants. J. Surfactants Deterg. *3*, 53–58. https://doi.org/10.1007/s11743-000-0113-4.

25. Tadros, T.F. (2005). Applications of Surfactants in Emulsion Formation and Stabilisation. In Applied Surfactants (John Wiley & Sons, Ltd), pp. 115–185. https://doi.org/10.1002/3527604812.ch6.

26. Attwood, D., and Florence, A.T. (1983). Surfactant Systems: Their Chemistry, Pharmacy and Biology (Springer Nature).

27. Golyshev, V.M., Abramova, T.V., Pyshnyi, D.V., and Lomzov, A.A. (2019). Structure and Hybridization Properties of Glycine Morpholine Oligomers in Complexes with DNA and RNA: Experimental and Molecular Dynamics Studies. J. Phys. Chem. B *123*, 10571–10581. https://doi.org/10.1021/acs.jpcb.9b07148.

28. Sponer, J., Bussi, G., Krepl, M., Banas, P., Bottaro, S., Cunha, R.A., Gil-Ley, A., Pinamonti, G., Poblete, S., Jurečka, P., et al. (2018). RNA structural dynamics as captured by molecular simulations: A comprehensive overview. Chem. Rev. *118*, 4177–4338. https://doi.org/10.1021/acs.chemrev.7b00427.

29. Zadymova, N.M., Yampol'skaya, G.P., and Filatova, L.Y. (2006). Interaction of bovine serum albumin with nonionic surfactant Tween 80 in aqueous solutions: Complexation and association. Colloid J. *68*, 162–172. https://doi.org/10.1134/S1061933X06020074.

30. Ruiz-Peña, M., Oropesa-Nuñez, R., Pons, T., Louro, S.R.W., and Pérez-Gramatges, A. (2010). Physico-chemical studies of molecular interactions between non-ionic surfactants and bovine serum albumin. Colloids Surf. B Biointerfaces *75*, 282–289. https://doi.org/10.1016/j.colsurfb.2009.08.046.

31. Arakawa, T., and Kita, Y. (2000). Protection of bovine serum albumin from aggregation by Tween 80. J. Pharm. Sci. *89*, 646–651. https://doi.org/10.1002/(SICI)1520-6017(200005)89:5<646::AID-JPS10>3.0.CO;2-J.

32. Singh, S.M., Bandi, S., Jones, D.N.M., and Mallela, K.M.G. (2017). Effect of Polysorbate 20 and Polysorbate 80 on the Higher-Order Structure of a Monoclonal Antibody and Its Fab and Fc Fragments Probed Using 2D Nuclear Magnetic Resonance Spectroscopy. J. Pharm. Sci. *106*, 3486–3498. https://doi.org/10.1016/j.xphs.2017.08.011.

33. Karjiban, R.A., Basri, M., Rahman, M.B.A., and Salleh, A.B. (2012). Structural Properties of Nonionic Tween80 Micelle in Water Elucidated by Molecular Dynamics Simulation. APCBEE Procedia *3*, 287–297. https://doi.org/10.1016/j.apcbee.2012.06.084.

34. Mobasheri, M., Attar, H., Sorkhabadi, S.M.R., Khamesipour, A., and Jaafari, M.R. (2016). Solubilization behavior of polyene antibiotics in nanomicellar system: Insights from molecular dynamics simulation of the amphotericin B and Nystatin interactions with polysorbate 80. Molecules *21*, E6. https://doi.org/10.3390/molecules21010006.

35. Luz, A.M., Barbosa, G., Manske, C., and Tavares, F.W. (2023). Tween-80 on Water/Oil Interface: Structure and Interfacial Tension by Molecular Dynamics Simulations. Langmuir *39*, 3255–3265. https://doi.org/10.1021/acs.langmuir.2c03001.

36. Lou, H., Wu, Y., Kuczera, K., and Schöneich, C. (2024). Coarse-Grained Molecular Dynamics Simulation of Heterogeneous Polysorbate 80 Surfactants and their Interactions with Small Molecules and Proteins. Mol. Pharm. *21*, 5041–5052. https://doi.org/10.1021/acs.molpharmaceut.4c00461.

37. Joshi, O., Chu, L., McGuire, J., and Wang, D.Q. (2009). Adsorption and function of recombinant factor VIII at the air-water interface in the presence of tween 80. J. Pharm. Sci. *98*, 3099–3107. https://doi.org/10.1002/jps.21569.

38. Otzen, D.E., Pedersen, J.N., Rasmussen, H.Ø., and Pedersen, J.S. (2022). How do surfactants unfold and refold proteins? Adv. Colloid Interface Sci. *308*, 102754. https://doi.org/10.1016/j.cis.2022.102754.

39. Poghosyan, A.H., Shahinyan, A.A., Kirakosyan, G.R., Ayvazyan, N.M., Mamasakhlisov, Y.S., and Papoian, G.A. (2021). A molecular dynamics study of protein denaturation induced by sulfonate-based surfactants. J. Mol. Model. *27*, 261. https://doi.org/10.1007/s00894-021-04882-2.

40. Chou, D.K., Krishnamurthy, R., Randolph, T.W., Carpenter, J.F., and Manning, M.C. (2005). Effects of Tween 20® and Tween 80® on the stability of Albutropin during agitation. J. Pharm. Sci. *94*, 1368–1381. https://doi.org/10.1002/jps.20365.

41. Rabe, M., Kerth, A., Blume, A., and Garidel, P. (2020). Albumin displacement at the air–water interface by Tween (Polysorbate) surfactants. Eur. Biophys. J. *49*, 533–547. https://doi.org/10.1007/s00249-020-01459-4.

42. Nichols, P., Li, L., Kumar, S., Buck, P.M., Singh, S.K., Goswami, S., Balthazor, B., Conley, T.R., Sek, D., and Allen, M.J. (2015). Rational design of viscosity reducing mutants of a monoclonal antibody: Hydrophobic versus electrostatic inter-molecular interactions. mAbs *7*, 212–230. https://doi.org/10.4161/19420862.2014.985504.

43. Hierrezuelo, J., Molina-Bolívar, J., and Ruiz, C. (2014). An Energetic Analysis of the Phase Separation in Non-Ionic Surfactant Mixtures: The Role of the Headgroup Structure. Entropy *16*, 4375–4391. https://doi.org/10.3390/e16084375.

44. Islam, M.N., and Kato, T. (2005). Influence of Temperature and Headgroup Size on Condensed-Phase Patterns in Langmuir Monolayers of Some Oxyethylenated Nonionic Surfactants. Langmuir *21*, 2419–2424. https://doi.org/10.1021/la047544p.

45. Wang, N., Hu, B., Ionescu, R.M., Mach, H., Sweeney, J.A., Hamm, C., Kirchmeier, M.J., and Meyer, B.K. (2009). Opalescence of an IgG1 Monoclonal Antibody Formulation is Mediated by Ionic Strength and Excipients. Biopharm Int. *22*, 36–47.

46. Tartaglia, G.G., Pawar, A.P., Campioni, S., Dobson, C.M., Chiti, F., and Vendruscolo, M. (2008). Prediction of Aggregation-Prone Regions in Structured Proteins. J. Mol. Biol. *380*, 425–436. https://doi.org/10.1016/j.jmb.2008.05.013.

47. Fernandez-Escamilla, A.M., Rousseau, F., Schymkowitz, J., and Serrano, L. (2004). Prediction of sequence-dependent and mutational effects on the aggregation of peptides and proteins. Nat. Biotechnol. *22*, 1302–1306. https://doi.org/10.1038/nbt1012.

48. Pavlova, A.S., Dyudeeva, E.S., Kupryushkin, M.S., Amirkhanov, N.V., Pyshnyi, D.V., and Pyshnaya, I.A. (2018). SDS-PAGE procedure: Application for characterization of new entirely uncharged nucleic acids analogs. Electrophoresis *39*, 670–674. https://doi.org/10.1002/elps.201700415.

49. Inácio, Â.S., Costa, G.N., Domingues, N.S., Santos, M.S., Moreno, A.J.M., Vaz, W.L.C., and Vieira, O.V. (2013). Mitochondrial Dysfunction Is the Focus of Quaternary Ammonium Surfactant Toxicity to Mammalian Epithelial Cells. Antimicrob. Agents Chemother. *57*, 2631–2639. https://doi.org/10.1128/aac.02437-12.

50. de Freitas Araújo Reis, M.Y., de Araújo Rêgo, R.I., Rocha, B.P., Guedes, G.G., de Medeiros Ramalho, Í.M., de Medeiros Cavalcanti, A.L., Guimarães, G.P., and de Lima Damasceno, B.P.G. (2021). A General Approach on Surfactants Use and Properties in Drug Delivery Systems. Curr. Pharm. Des. *27*, 4300–4314. https://doi.org/10.2174/1381612827666210526091825.

51. FDA (2012). Package Insert - Fluarix Quadrivalent. https://www.fda.gov/media/84804/download.

52. FDA Package Insert - Fluzone High-Dose Quadrivalent. https://www.fda.gov/media/132238/download.

53. Bodratti, A.M., and Alexandridis, P. (2018). Formulation of Poloxamers for Drug Delivery. J. Funct. Biomater. *9*, 11. https://doi.org/10.3390/jfb9010011.

54. Rey-Rico, A., and Cucchiarini, M. (2018). PEO-PPO-PEO Tri-Block Copolymers for Gene Delivery Applications in Human Regenerative Medicine—An Overview. Int. J. Mol. Sci. *19*, 775. https://doi.org/10.3390/ijms19030775.

55. Yue, L., Yan, Z., Li, H., Liu, X., and Sun, P. (2020). Brij-58, a potential injectable protein-stabilizer used in therapeutic protein formulation. Eur. J. Pharm. Biopharm. *146*, 73–83. https://doi.org/10.1016/j.ejpb.2019.12.001.

56. de Fougerolles, A., and Elbashir, S.M. (2021). Delivery and formulation of engineered nucleic acids, US010898574B2.

57. Wu, B., Morgan, D., Lu, P., Lu, Q., and Wang, M. (2020). Neutral Surfactant Enhanced Exon-skipping of Morpholino Oligonucleotides in vitro and in MDX Mice. J. Nanomed. Nanotechnol. *11*, 553.

58. Kliuchnikov, E., Maksudov, F., Pierson, D., Marx, K.A., Chanda, A., and Barsegov, V. (2025). Molecular properties and intramolecular interactions of peptide-conjugated phosphorodiamidate morpholino oligonucleotides. Mol. Ther. Nucleic Acids *36*, 102685. https://doi.org/10.1016/j.omtn.2025.102685.

59. Humphrey, W., Dalke, A., and Schulten, K. (1996). VMD: Visual molecular dynamics. J. Mol. Graph. *14*, 33–38. https://doi.org/10.1016/0263-7855(96)00018-5.

60. Caleman, C., Van Maaren, P.J., Hong, M., Hub, J.S., Costa, L.T., and Van Der Spoel, D. (2012). Force field benchmark of organic liquids: Density, enthalpy of vaporization, heat capacities, surface tension, isothermal compressibility, volumetric expansion coefficient, and dielectric constant. J. Chem. Theory Comput. *8*, 61–74. https://doi.org/10.1021/ct200731v.

61. Harikrishna, S., and Pradeepkumar, P.I. (2017). Probing the Binding Interactions between Chemically Modified siRNAs and Human Argonaute 2 Using Microsecond Molecular Dynamics Simulations. J. Chem. Inf. Model. *57*, 883–896. https://doi.org/10.1021/acs.jcim.6b00773.

62. Bottaro, S., Bussi, G., Kennedy, S.D., Turner, D.H., and Lindorff-Larsen, K. (2018). Conformational ensembles of RNA oligonucleotides from integrating NMR and molecular simulations. Sci. Adv. *4*, eaar8521. https://doi.org/10.1126/sciadv.aar8521.

63. Salomon-Ferrer, R., Götz, A.W., Poole, D., Le Grand, S., and Walker, R.C. (2013). Routine microsecond molecular dynamics simulations with AMBER on GPUs. 2. Explicit solvent particle mesh ewald. J. Chem. Theory Comput. *9*, 3878–3888. https://doi.org/10.1021/ct400314y.

64. Darden, T., York, D., and Pedersen, L. (1993). Particle mesh Ewald: An N·log(N) method for Ewald sums in large systems. J. Chem. Phys. *98*, 10089–10092. https://doi.org/10.1063/1.464397.

65. Le Grand, S., Götz, A.W., and Walker, R.C. (2013). SPFP: Speed without compromise - A mixed precision model for GPU accelerated molecular dynamics simulations. Comput. Phys. Commun. *184*, 374–380. https://doi.org/10.1016/j.cpc.2012.09.022.

66. Case, D.A., Belfon, K., Ben-Shalom, I.Y., Brozell, S.R., Cerutti, D.S., Cheatham, T.E., III, Cruzeiro, V.W.D., Darden, T.A., Duke, R.E., Giambasu, G., et al. (2020). AMBER 2020 (San Francisco: University of California).

67. Hou, T., Wang, J., Li, Y., and Wang, W. (2011). Assessing the performance of the MM/PBSA and MM/GBSA methods. 1. The accuracy of binding free energy calculations based on molecular dynamics simulations. J. Chem. Inf. Model. *51*, 69–82. https://doi.org/10.1021/ci100275a.

68. Miller, B.R., 3rd, McGee, T.D., Jr., Swails, J.M., Homeyer, N., Gohlke, H., and Roitberg, A.E. (2012). MMPBSA.py: An Efficient Program for End-State Free Energy Calculations. J. Chem. Theory Comput. *8*, 3314–3321. https://doi.org/10.1021/ct300418h.

69. Johnson, W.C. (1996). Determination of the Conformation of Nucleic Acids by Electronic CD. In Circular Dichroism and the Conformational Analysis of Biomolecules, G.D. Fasman, ed. (Springer US), pp. 433–468. https://doi.org/10.1007/978-1-4757-2508-7_12.

70. Micsonai, A., Wien, F., Kernya, L., Lee, Y.-H., Goto, Y., Réfrégiers, M., and Kardos, J. (2015). Accurate secondary structure prediction and fold recognition for circular dichroism spectroscopy. Proc. Natl. Acad. Sci. USA *112*, E3095–E3103. https://doi.org/10.1073/pnas.1500851112.

71. Chin, S.L., Lu, Q., Dane, E.L., Dominguez, L., McKnight, C.J., Straub, J.E., and Grinstaff, M.W. (2016). Combined Molecular Dynamics Simulations and Experimental Studies of the Structure and Dynamics of Poly-Amido-Saccharides. J. Am. Chem. Soc. *138*, 6532–6540. https://doi.org/10.1021/jacs.6b01837.

72. Bulheller, B.M., and Hirst, J.D. (2009). DichroCalc—circular and linear dichroism online. Bioinformatics *25*, 539–540. https://doi.org/10.1093/bioinformatics/btp016.

OMTN, Volume *37*

Supplemental information

Polysorbates' effects on molecular and thermodynamic properties of phosphorodiamidate morpholino oligonucleotides' structures

Evgenii Kliuchnikov, Daniel Pierson, Ying Chou, Willow DiLuzio, Kenneth A. Marx, Arani Chanda, and Valeri Barsegov

Supplemental Methods

Force field development for all-atom simulations of PMO with surfactant molecules: In our previous study [1], we derived the atomic partial charges and force-field parameters for the following three parts of the PMO structure: i) for the phosphorodiamidate group attached to the 5'-end and capping the HN atomic group attached to the N3' atom (mimicking the PMO 5'-end); ii) for the phosphorodiamidate group attached to the 3'-end and capping the HO atomic group attached to the O5' atom (mimicking the PMO 3'-end); and iii) for the phosphorodiamidate group attached to both 5'- and 3'-ends. In the same study [1], we also derived the force field parameters and partial charges for the morpholino triethylene glycol (TEG) piperazine linker attached to the 5'-end of PMO through the phosphorodiamidate group. For both surfactants Polysorbate 20 and Polysorbate 80, we first reconstructed the initial structure based on the 2D-schemes (Fig 1C in the main text) in GaussView 5.0 [2]. Next, we performed energy minimization and optimization of the structures, using Hartree-Fock (HF) theory with the 6-31G* basis set using the Gaussian 16 package [3]. In the derivation of partial charges for the PMO structure carried out in our previous study [1], as well as for the structures of Polysorbate 20 and Polysorbate 80, we employed the Restrained Electro Static Potential (PESP) charge fitting procedure [4], implemented in the RED server (RESP ESP charge Derive Server) [5]. We utilized the RESP method in conjunction with HF theory and the 6-31G* basis set. Several *ab initio* calculations were carried out for each fragment, including the morpholino ring (5'-, 3'-, and central) with guanine, cytosine, thymine, and adenine bases, with the TEG piperazine linker, and for the entire Polysorbate 20 and Polysorbate 80 molecules. For each fragment, we carried out 3 independent runs (total of 39 runs) over which the final partial charges were averaged. The Antechamber package [6] was employed to assign the all-atom types for atoms in these fragments. Molecular mechanical parameters were obtained from the bsc0$_{\chi OL3}$ force field for nucleic acids and from the ff14SB force field for proteins, for these atom types. These include the equilibrium bond distances (r_0), spring constants for covalent bonds (k_b), equilibrium bond angles (θ_0), and spring constants for bending of bond angles (k_θ), as well as torsional angle parameters (magnitude associated with torsion energy ($V_n/2$), phase offset (γ) and periodicity (n)). For the atom types not covered in the bsc0$_{\chi OL3}$ and ff14SB force fields, the molecular mechanical parameters were generated using the general Amber force field GAFF [7]. For model details of the force field development procedure, including the molecular mechanics parameters and partial charges, the reader should consult our previous work [1] (see also the SI in Ref. [1]). For each PMO molecule and for each surfactant molecule, we created the topology files for each of the "morpholino nucleotides", for the TEG piperazine linker, and for the surfactants. Next, we linked "morpholino nucleotides" together through the phosphorodiamidate groups and added the TEG piperazine linker at the 5'-end (see Fig. 1 in the main text). These structures were then used in all the MD simulations described in the main text part.

Analysis of MD simulation output: The results of MD simulations for PMO-surfactant complexes (coordinate and energy files) were used in data analysis and visualization. *The radius of gyration R_g* was calculated using the coordinates of all atoms,

$$R_g = (\sum_p m_p \boldsymbol{r}_p^2 / \sum_p m_p)^{1/2} \qquad (Equation\ S1)$$

where m_p is the mass and $\boldsymbol{r}_p$ is the position of atom p, relative to the center of mass of the molecule. *Base stacking and base pairing:* If three conditions are satisfied:

$$|z_{kj}|\ and\ |z_{jk}|\ >\ 2\text{Å} \qquad (Equation\ S2)$$

and

$$\rho_{kj}\ or\ \rho_{jk} <\ 2.5\text{Å} \qquad (Equation\ S3)$$

and

$$\left|\theta_{kj}\right| < 40° \qquad (Equation\ S4)$$

bases were categorized as stacked. Here,

$$\rho_{ij} = \sqrt{x_{kj}^2 + y_{kj}^2} \qquad (Equations\ S5)$$

where the x- and y-axes lie in the plane of the base (x_{kj} and y_{kj} are the distances between the centers of mass of the two bases along the x- and y-axes, respectively) and the z-axis is perpendicular to the xy-plane, z_{kj} is the distance between the centers of mass of the two bases, and θ_{kj} is the angle between the normal vectors of the two bases (see Fig. S1 in Ref. [8]). All the non-stacked bases are considered to be base-paired if $\left|\theta_{kj}\right| < 60°$ and there is at least one hydrogen bond (H-bond) between k-th and j-th bases (see Fig. S1 in Ref. [8]). The total number of base pairs and number of base stackings were calculated using Barnaba software [8] with the structure schematic shown in Fig. S1 in Ref. [1] guiding the calculation. *The Solvent Accessible Surface Area* (SASA) was estimated using the LCPO algorithm [9] implemented in the CPPTRAJ module [10] in AmberTools20 [11]. For RMSD analysis, all frames in the simulation trajectories were superimposed onto the initial energy-minimized structure of the PMO or the PMO–surfactant complex after removing all water molecules. The RMSD was then calculated using the atom selection keyword "nucleic" in VMD, which includes atoms from both the backbone and the nucleobases of the nucleic acid. We used the formula

$$RMSD = \sqrt{\frac{1}{N}\sum_{i=1}^{N} {\delta_i}^2} \qquad (Equation\ S6)$$

where δ_i is the distance between atom i in the current frame and its corresponding atom in the reference structure at $t = 0$, and N is the number of atoms in the nucleic acid selection. *PMO-PMO and PMO-surfactant interactions:* We assume that a pair of atomic groups (in modified nucleotides of PMOs and surfactant tails in Polysorbate 80 and Polysorbate 20) forms a contact if the distance between the center of mass of the modified nucleotide and any C or O atom in surfactant' tails $d_{RES} < 7.5$Å-cutoff. The statistics of the distances was obtained with the MDAnalysis Python package [12,13]. *Hydrogen bonds:* We assume that the H-bond D–H…A between the hydrogen donor atom (D) and acceptor atom (A) is formed if the donor–acceptor distance $d_{DA} < 3.3$Å-cutoff and if the bond angle is larger than the 140°-cutoff [8]. To find the time fraction for each interaction, we used the H-bond option in the CPPTRAJ module [14] implemented in AmberTools20 [11].

Classification of Polysorbate 80 conformations: Based on the numerical output from the all-atom MD simulations of Polysorbate 80, a total of 500 conformations (structure snapshots) of Polysorbate 80 structures were selected for the statistical modeling and classification. For each of these 500 conformations, we calculated the corresponding values of R_g, $SASA$, $RMSD$, L and W described in the main text. The reference structures for calculation of RMSD values for Polysorbate 80 were set to the initial structure of Polysorbate 80 (at time $t = 0$). By combining these values together, we constructed a dataset containing the values of R_g, $SASA$, $RMSD$, L and W for structure classification. We employed the Support Vector Machines (SVM) approach to perform data classification of Polysorbate 80 molecular conformations into the "collapsed conformations" and the "extended conformations". "Classify method" option implemented in Mathematica with radial basis function (RBF) kernel was used as a numerical implementation of the SVM algorithm [15]. The centroids of each class (central structures) for the extended and collapsed conformation class were constructed as the average collapsed conformation and the average extended conformation. The results obtained are displayed in Fig. 4B in the main text.

Supplemental references:

1. Maksudov, F., Kliuchnikov, E., Pierson, D., Ujwal, M.L., Marx, K.A., Chanda, A., and Barsegov, V. (2023). Therapeutic phosphorodiamidate morpholino oligonucleotides: Physical properties, solution structures, and folding thermodynamics. Molecular Therapy-Nucleic Acids *31*, 631–647.
2. Dennington, R., Keith, T., Millam, J., and others (2009). GaussView, version 5.
3. Frisch, M.J., Trucks, G.W., Schlegel, H.B., Scuseria, G.E., Robb, M.A., Cheeseman, J.R., Scalmani, G., Barone, V., Petersson, G.A., Nakatsuji, H., et al. (2016). Gaussian 16. Preprint at Gaussian, Inc. Wallingford, CT.
4. Cieplak, P., Cornell, W.D., Bayly, C., and Kollman, P.A. (1995). Application of the multimolecule and multiconformational RESP methodology to biopolymers: Charge derivation for DNA, RNA, and proteins. J Comput Chem *16*, 1357–1377.
5. Vanquelef, E., Simon, S., Marquant, G., Garcia, E., Klimerak, G., Delepine, J.C., Cieplak, P., and Dupradeau, F.-Y. (2011). RED Server: a web service for deriving RESP and ESP charges and building force field libraries for new molecules and molecular fragments. Nucleic Acids Res *39*, W511–W517.
6. Wang, J., Wang, W., Kollman, P.A., and Case, D.A. (2006). Automatic atom type and bond type perception in molecular mechanical calculations. J Mol Graph Model *25*, 247–260.
7. Wang, J., Wolf, R.M., Caldwell, J.W., Kollman, P.A., and Case, D.A. (2004). Development and testing of a general amber force field. J Comput Chem *25*, 1157–1174.
8. Bottaro, S., Bussi, G., Pinamonti, G., Reißer, S., Boomsma, W., and Lindorff-Larsen, K. (2019). Barnaba: software for analysis of nucleic acid structures and trajectories. Rna *25*, 219–231.
9. Weiser, J., Shenkin, P.S., and Still, W.C. (1999). Approximate atomic surfaces from linear combinations of pairwise overlaps (LCPO). J Comput Chem *20*, 217–230.
10. Roe, D.R., and Cheatham III, T.E. (2013). PTRAJ and CPPTRAJ: software for processing and analysis of molecular dynamics trajectory data. J Chem Theory Comput *9*, 3084–3095.
11. Case, D.A., Belfon, K., Ben-Shalom, I., Brozell, S.R., Cerutti, D., Cheatham, T., Cruzeiro, V.W.D., Darden, T., Duke, R.E., Giambasu, G., et al. (2020). Amber 2020. Preprint.
12. Michaud-Agrawal, N., Denning, E.J., Woolf, T.B., and Beckstein, O. (2011). MDAnalysis: a toolkit for the analysis of molecular dynamics simulations. J Comput Chem *32*, 2319–2327.
13. Gowers, R.J., Linke, M., Barnoud, J., Reddy, T.J.E., Melo, M.N., Seyler, S.L., Domanski, J., Dotson, D.L., Buchoux, S., Kenney, I.M., et al. (2016). MDAnalysis: a Python package for the rapid analysis of molecular dynamics simulations. In Proceedings of the 15th python in science conference, p. 105.
14. Roe, D.R., and Cheatham III, T.E. (2013). PTRAJ and CPPTRAJ: software for processing and analysis of molecular dynamics trajectory data. J Chem Theory Comput *9*, 3084–3095.
15. Suchok, S. (2015). Mathematica Data Analysis (Packt Publishing Ltd).

Supplemental Movies

Movie S1. 25-mer conformer III interacting with Polysorbate 80: The movie shows the molecular interactions between the 25-mer PMO and Polysorbate 80 as observed in a 1-μs MD simulation at $T = 300$ K. The MD simulation run was carried out in explicit water (cyan transparent spheres). The PMO molecule is shown in the Twister representation for the backbone (blue line) and paper chains for the nucleobases (red and green). Polysorbate 80 is shown in the Licorice representation (sticks). The length of the movie is 50 s (the movie is played 5×10^7 times slower than the computational experiment).

Movie S2. 30-mer conformer III interacting with Polysorbate 80: The movie shows the molecular interactions between the 30-mer PMO and Polysorbate 80 as observed in a 2.75-μs MD simulation at $T = 300$ K. The MD simulation run was carried out in explicit water (cyan transparent spheres). The PMO molecule is shown in the Twister representation for the backbone (blue line) and paper chains for the nucleobases (red and green). Polysorbate 80 is shown in the Licorice representation (sticks). The length of the movie is 138 s (the movie is played 5×10^7 times slower than the computational experiment).

Movie S3. Polysorbate 80 equilibrium dynamics: The movie shows the conformational fluctuations of Polysorbate 80 between the extended and collapsed conformations observed in a 1-μs MD simulation run at $T = 300$ K. The MD simulation run was carried out in explicit water (cyan transparent spheres). Polysorbate 80 is shown in the Licorice representation (sticks). The length of the movie is 39 s (the movie is played 3.9×10^7 times slower than the computational experiment).

Movie S4. 30-mer conformer I interacting with Polysorbate 80: The movie shows the molecular interactions between 30-mer conformer I PMO and Polysorbate 80 as observed in a 1-μs MD simulation at T = 300K. The MD simulation run was carried out in explicit water (cyan transparent spheres). The PMO molecule is shown in the Twister representation for the backbone (blue line) and paper chains for the nucleobases (red and green). Polysorbate 80 is shown in the Licorice representation (sticks). The length of the movie is 106 s (the movie is played 1.1×10^8 times slower than the computational experiment).

Supplemental Tables

Table S1. CAC, CMC and PMO: Surfactant interaction stoichiometry (*n*:1) analysis from surface tension data: Shown for the 22-mer, 25-mer, and 30-mer PMO solutions with Polysorbate 80 and Polysorbate 20 surfactants are the critical aggregation concentration (CAC), the critical micelle concentration (CMC), and the relative number of surfactant molecules per one PMO molecule n at CMC The CMC values are also given for solutions of both Polysorbate 80 and Polysorbate 20 surfactants in the absence of PMOs (molecular weights of Polysorbate 80 and Polysorbate 20 surfactants are 1310 amu and 1227.5 amu, respectively).

	Polysorbate 80			Polysorbate 20		
	CAC (mg/mL)	CMC (mg/mL)	*n*	CAC (mg/mL)	CMC (mg/mL)	*n*
No PMO	NA	0.016	NA	NA	0.068	NA
30-mer	0.0039	3.68	1.74	0.0094	2.10	2.86
25-mer	0.0035	3.93	1.93	0.0084	2.53	2.80
22-mer	0.0022	5.18	1.67	0.0059	4.22	1.92

Table S2. Molecular Mechanical parameters for covalent bonds in surfactants Polysorbate 20 and Polysorbate 80: Shown for each covalent bond are the equilibrium covalent bond distance r_0 and spring constant k_b. These force field parameters were determined for the surfactant molecules explored in this study (see Material and Methods in the main text). Atom types: OH - sp^3 hybridized oxygen in hydroxyl group; HO – hydrogen on oxygen; CT – sp^3 hybridized carbon with 4 explicit substituents; H1 – hydrogen on aliphatic carbon with 1 electron-withdrawing group; HC – hydrogen on aliphatic carbon; OS – ester oxygen; C - sp^2 hybridized carbon in C=O and C=S; O - sp^2 hybridized oxygen in C=O and COO^-; C2 – aliphatic sp^2 hybridized carbon.

Bond	k_b, kcal/(mol·Å^2)	r_0, Å
OH-HO	371.4	0.973
OH-CT	316.7	1.423
CT-CT	300.9	1.537
CT-H1	330.6	1.097
CT-HC	330.6	1.097
CT-OS	308.6	1.432
OS-C	390.8	1.358
C-O	637.7	1.218
C-CT	313.0	1.524
CT-C2	326.8	1.509
C2-C2	569.4	1.334
C2-HC	344.3	1.087

Table S3. Molecular Mechanical parameters for bond angles in surfactants: Shown for each bond angle are the equilibrium bond angle θ_0 and bending angle spring constant k_a.

Angle	k_a, kcal/(mol·rad^2)	θ_0,deg
HO-OH-CT	47.4	107.26
OH-CT-CT	67.5	110.19
OH-CT-HC	51.1	109.50
HC-CT-HC	39.4	107.58
CT-CT-HC	46.3	109.80
CT-CT-OS	68.0	107.97
OS-CT-HC	51.0	108.70
CT-OS-CT	62.7	112.48
CT-CT-CT	62.9	111.51
CT-OS-C	63.3	115.98
OS-C-O	75.3	123.25
O-C-CT	67.4	123.20
C-CT-HC	46.9	108.77
C-CT-CT	63.3	111.04
CT-C2-C2	64.1	123.63
C2-CT-HC	47.0	110.36
CT-C2-HC	45.1	120.00
C2-C2-HC	50.0	119.70

Table S4 Molecular Mechanical parameters for torsion angles in surfactant molecules: Shown for each torsion angle are the number of bond paths and the magnitude of torsion energy $V_n/2$, the phase offset γ, and the periodicity of torsion n. Atom X denotes any atom of the atom type described in the caption to Table S1.

Torsion angle	no. of paths	V_n/2, kcal/mol	γ, deg	n
HO-OH-CT-HC	3	0.500	0	3
HO-OH-CT-CT	1	0.250	0	1
X-CT-CT-X	1	0.250	0	1
OH-CT-CT-OS	1	1.175	0	2
CT-CT-OS-CT	1	0.100	180	2
X-OS-CT-X	3	1.150	0	3
OS-CT-CT-OS	1	1.175	0	2
HC-CT-CT-HC	1	0.150	0	3
X-CT-CT-X	9	1.400	0	3
CT-CT-CT-CT	1	0.200	180	1
CT-CT-CT-HC	1	0.160	0	3
CT-CT-OS-C	1	0.800	180	1
CT-OS-C-O	1	1.400	180	1
X-C-CT-X	6	0.000	180	2
O-C-CT-HC	1	0.080	180	3
X-CT-C2-X	6	0.000	0	2
CT-C2-C2-CT	1	1.900	180	1
CT-C2-C2-HC	4	26.600	180	2

Table S5. Dynamic properties of surfactant molecules obtained from MD simulations of PMO-surfactant complexes: Shown for each PMO-surfactant combination are the properties of surfactants: radius of gyration R_g, number of hydrogen bonds formed n_{hb}, solvent accessible surface area ($SASA$), root-mean-square deviation $RMSD$, length L, and width W (averages and standard deviations).

Surfactant	PMO	R_g, nm	n_{hb}	$SASA$, Å^2	$RMSD$, nm	L, nm	W, nm
Polysorbate 80	w/o PMO	0.77±0.08	0.10±0.06	1,757±177	1.22±0.15	1.49±0.61	1.58±0.56
Polysorbate 20	w/o PMO	0.77±0.08	0.10±0.07	1,696±171	1.01±0.12	1.48±0.62	1.56±0.55
Polysorbate 80	25mer I	0.88±0.11	0.07±0.05	1,282±219	2.70±0.60	1.78±0.69	1.75±0.53
Polysorbate 20	25mer I	0.84±0.10	0.08±0.07	1,185±209	2.53±0.32	1.76±0.76	1.57±0.56
Polysorbate 80	25mer II	0.92±0.11	0.07±0.06	1,221±168	2.21±0.40	1.98±0.66	1.79±0.61
Polysorbate 20	25mer II	0.85±0.09	0.06±0.05	1,156±262	2.60±0.49	1.79±0.67	1.75±0.56
Polysorbate 80	25mer III	0.86±0.09	0.08±0.05	1,234±184	1.92±0.52	1.68±0.64	1.70±0.52
Polysorbate 20	25mer III	0.87±0.08	0.07±0.05	1,180±172	2.05±0.41	1.92±0.62	1.69±0.54
Polysorbate 80	30mer I	0.87±0.10	0.06±0.05	1,161±208	2.49±0.58	1.71±0.64	1.73±0.53
Polysorbate 20	30mer I	0.86±0.10	0.09±0.07	1,087±160	2.44±0.50	1.94±0.77	1.69±0.56
Polysorbate 80	30mer II	0.88±0.11	0.07±0.06	1,243±202	2.62±0.69	1.74±0.66	1.74±0.56
Polysorbate 20	30mer II	0.87±0.10	0.07±0.05	1,161±220	2.09±0.44	2.14±0.70	1.71±0.54
Polysorbate 80	30mer III	0.86±0.10	0.09±0.06	1,093±250	2.49±0.42	1.82±0.65	1.65±0.57
Polysorbate 20	30mer III	0.89±0.09	0.07±0.05	1,077±241	2.37±0.38	2.00±0.81	1.63±0.54

Table S6. Interaction energies stabilizing the PMO-surfactant complexes: Shown are the components of the interaction energy (averages and standard deviations) between 25-mer and 30-mer PMO principal solution conformers I–III and surfactants Polysorbate 20 and Polysorbate 80: the van der Waals interaction energy ΔE_{vdW}; the electrostatic interaction energy ΔE_{el}, the solvation free energy ΔE_{solv}, and the total interaction energy ΔE.

PMO	Surfactant	ΔE_{vdW}, kcal/mol	ΔE_{el}, kcal/mol	ΔE_{solv}, kcal/mol	ΔE, kcal/mol
25mer I	Polysorbate 80	-73±24	-27±13	45±15	-55±21
25mer I	Polysorbate 20	-66±23	-23±14	41±15	-53±21
25mer II	Polysorbate 80	-81±21	-25±13	45±14	-64±17
25mer II	Polysorbate 20	-73±26	-25±14	43±16	-59±24
25mer III	Polysorbate 80	-76±23	-26±13	42±14	-64±21
25mer III	Polysorbate 20	-74±21	-28±13	45±14	-63±17
30mer I	Polysorbate 80	-85±26	-29±15	50±16	-64±23
30mer I	Polysorbate 20	-77±21	-25±12	44±13	-65±14
30mer II	Polysorbate 80	-76±20	-22±11	42±12	-60±16
30mer II	Polysorbate 20	-70±22	-21±11	40±13	-54±15
30mer III	Polysorbate 80	-86±25	-26±13	50±15	-62±18
30mer III	Polysorbate 20	-86±25	-31±13	54±17	-67±21

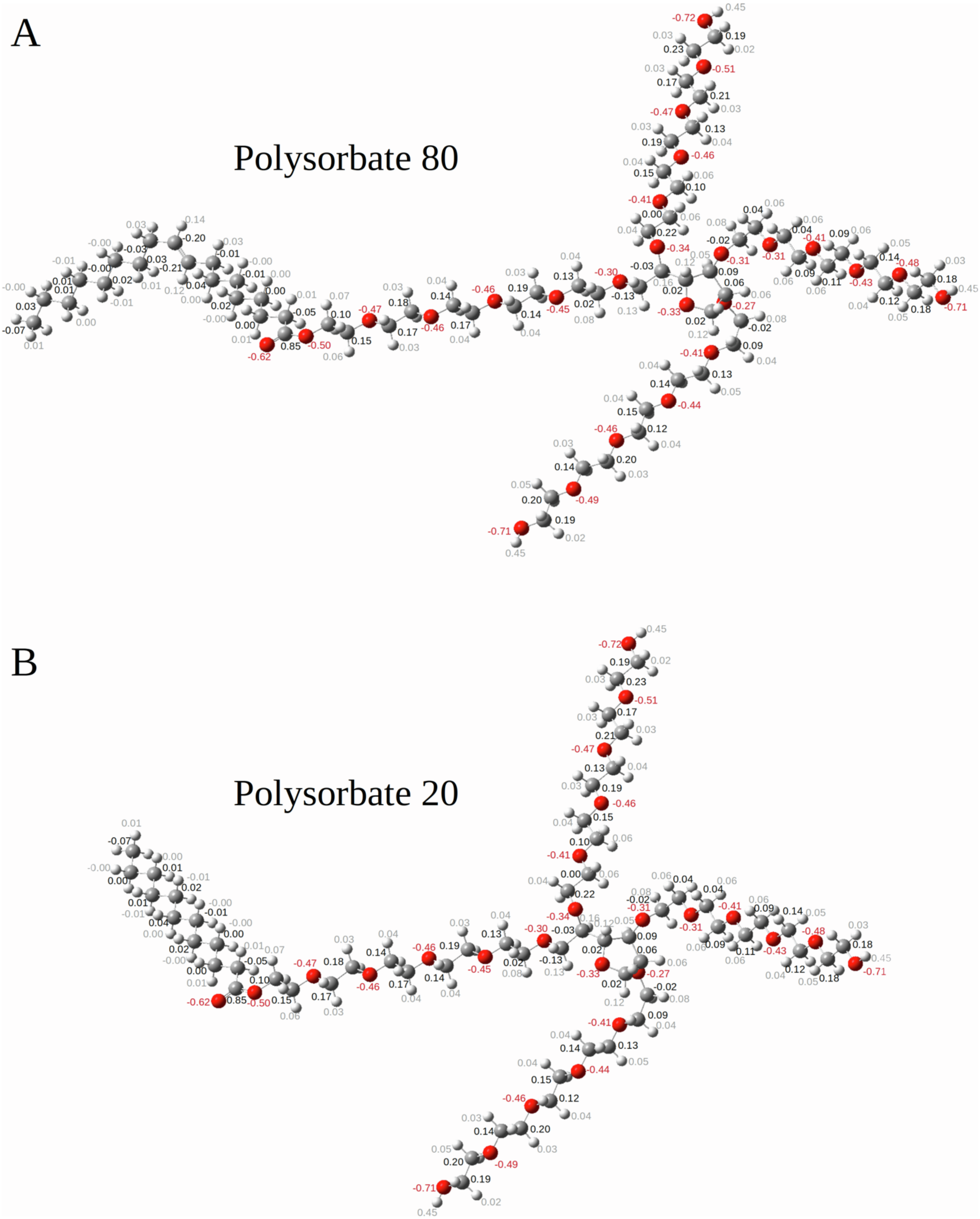

Figure S1. Atomic partial charges for surfactants Polysorbate 80 and Polysorbate 20. Panel **A**: Partial charges for Polysorbate 80. Panel **B**: Partial charges for Polysorbate 20. All atomic partial charges were calculated using the RESP method (see SI Methods).

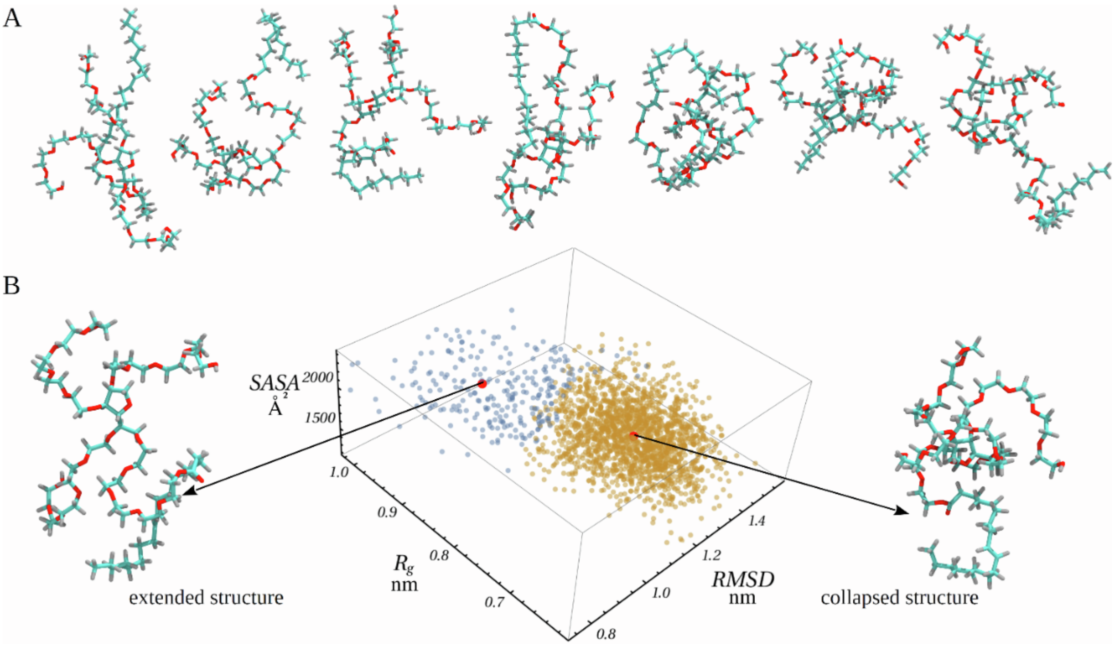

Figure S2. Polysorbate 80's conformations from 1-μs all-atom MD simulations. Seven randomly chosen conformations of Polysorbate 80 show that this surfactant is not able to form a stable structure (panel **A**). These structures were extracted from the set of a total of 500 structures of Polysorbate 80 generated in the 1-μs equilibrium MD simulation run. All 500 structures are shown in a form of 3D scatterplot of R_g vs. $RMSD$ and vs. $SASA$ (panel **B**). The Support Vector Classifier was used to separate the conformations that belong to the 'collapsed' class (yellow dots) and the 'extended' class of conformers (blue dots) as defined in SI. For each cluster, the centroids (red dots) were selected to be shown as the most representative structures.

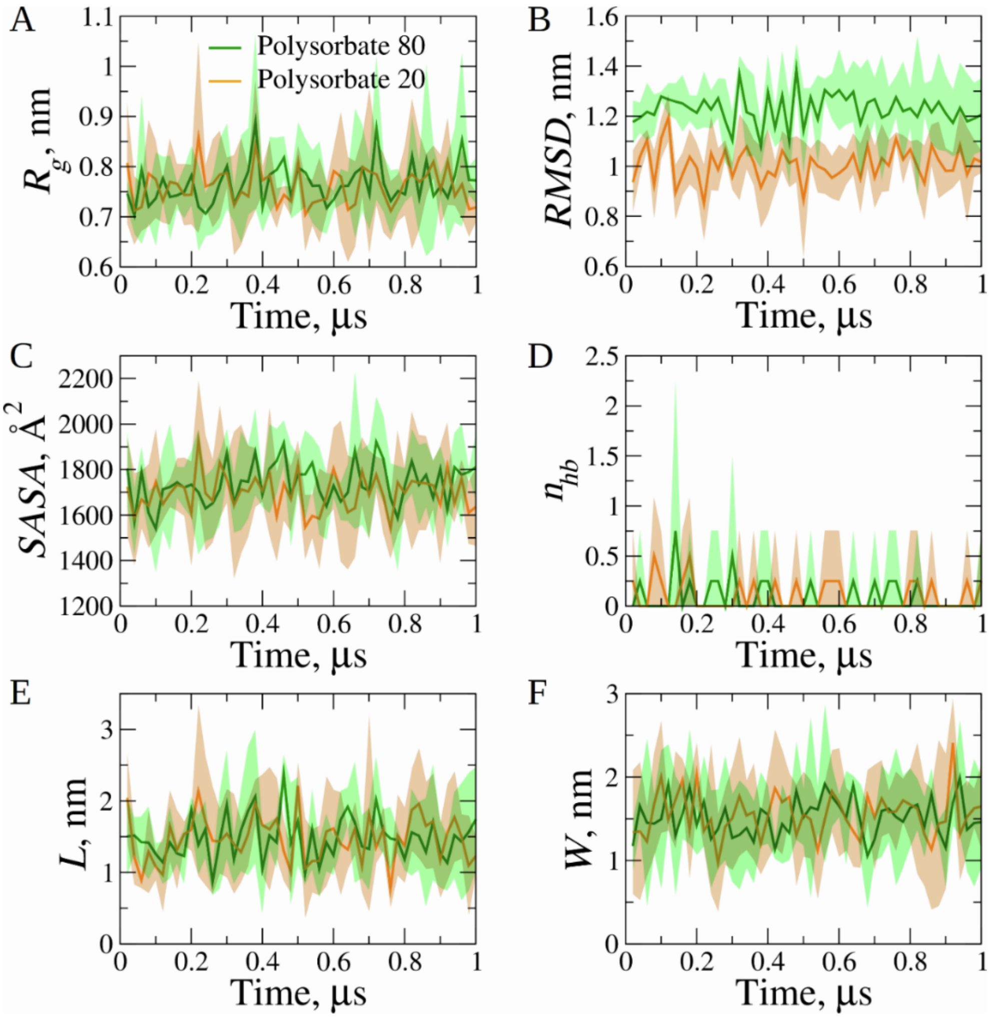

Figure S3. Dynamic structural properties of the surfactants Polysorbate 20 and Polysorbate 80. Time profiles of the radius of gyration R_g (panel **A**), $RMSD$ (panel **B**), $SASA$ (panel **C**), number of hydrogen bonds n_{hb} (panel **D**), length L (panel **E**), and width W (panel **F**). Shown are the averages and standard deviations for Polysorbate 80 (in green color) and Polysorbate 20 (in orange) from 1-µs long MD simulation runs for Polysorbate 20 and Polysorbate 80 (without PMO molecules).

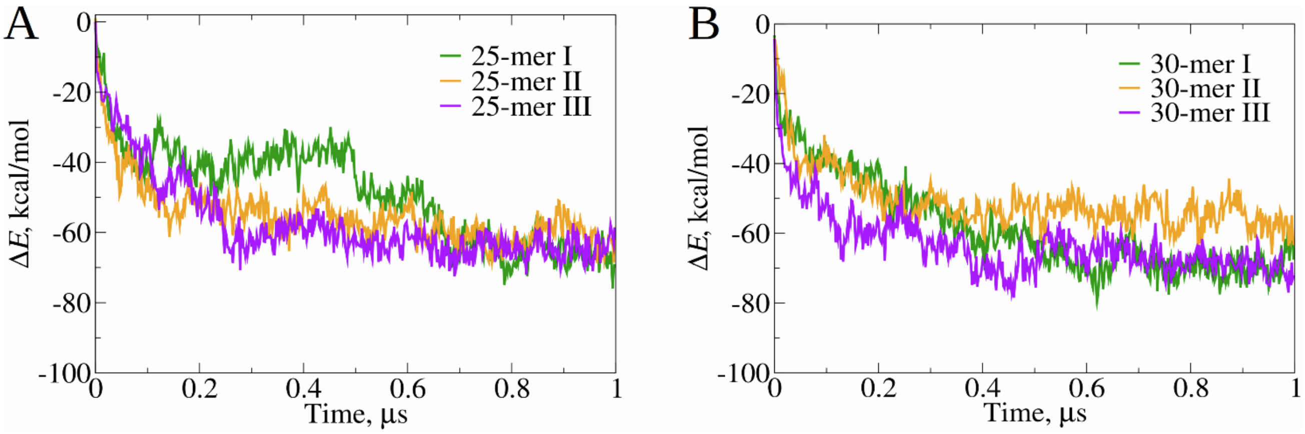

Figure S4. PMO-Polysorbate 20 interaction energies for 25-mer and 30-mer PMOs. Displayed are the time profiles of the total interaction energy ΔE between Polysorbate 20 and the 25-mer PMO conformer I (green), conformer II (orange) and conformer III (purple; panel **A**), and the 30-mer PMO conformer I (green), conformer II (orange) and conformer III (purple; panel **B**). Shown are the averages obtained from 10 all-atom MD simulation runs (see Materials and Methods in the main text). The PMO-Polysorbate 80 interaction energies for the 25-mer and 30-mer PMOs are shown in Fig. 5 in the main text.

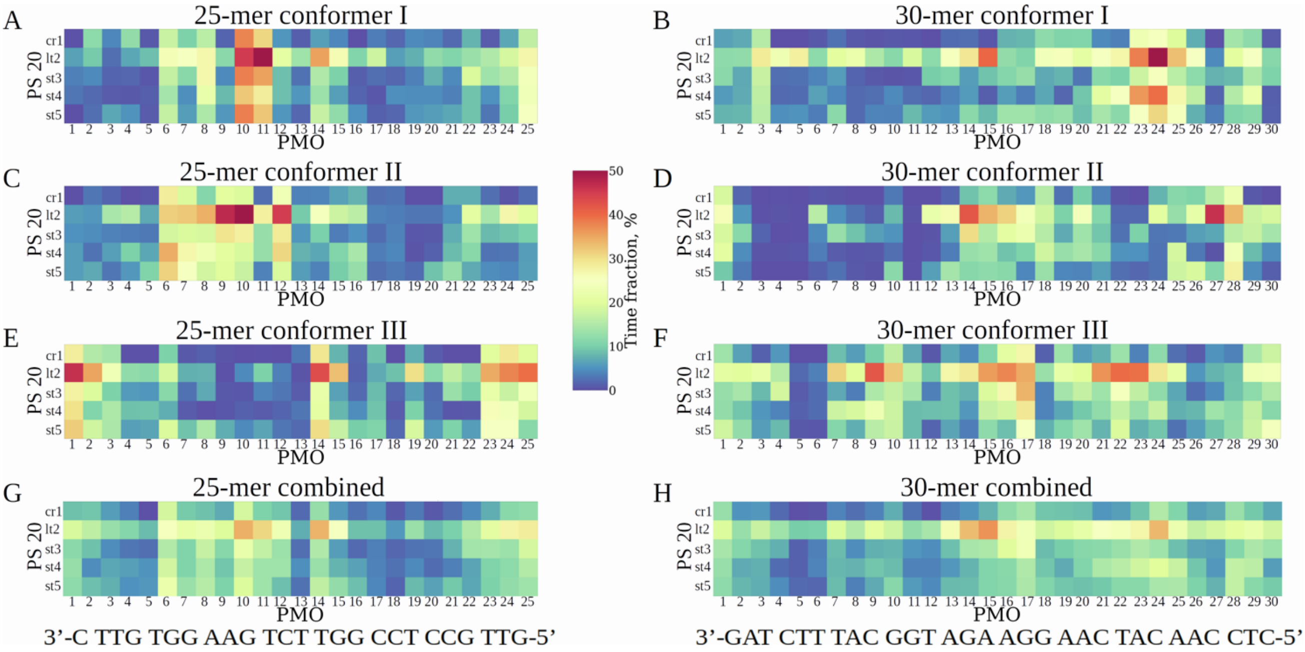

Figure S5. PMO-Polysorbate 20 interactions. Displayed are maps showing the time fraction (percentage of total time) of persistent interactions between PMO bases and surfactant side chains for the 25-mer PMO conformer I (panel **A**), conformer II (panel **C**) and conformer III (panel **E**) as well as the for the 30-mer PMO conformer I (panel **B**), conformer II (panel **D**) and conformer III (panel **F**). Also shown are the interactions maps for all three conformers of 25-mer (panel **G**) and 30-mer (panel **H**) combined. The nomenclature for PMO bases starts from the 3'-end of the PMO, and for the surfactant, cr1 is the central ring (see Fig. 1D), lt2 is the long hydrophobic tail (see Fig. 1D), and st3, st4 and st5 are the short tails (see Fig. 1D). The PMO-Polysorbate 80 interaction maps are shown in Fig. 6 in the main text. PS = Polysorbate.

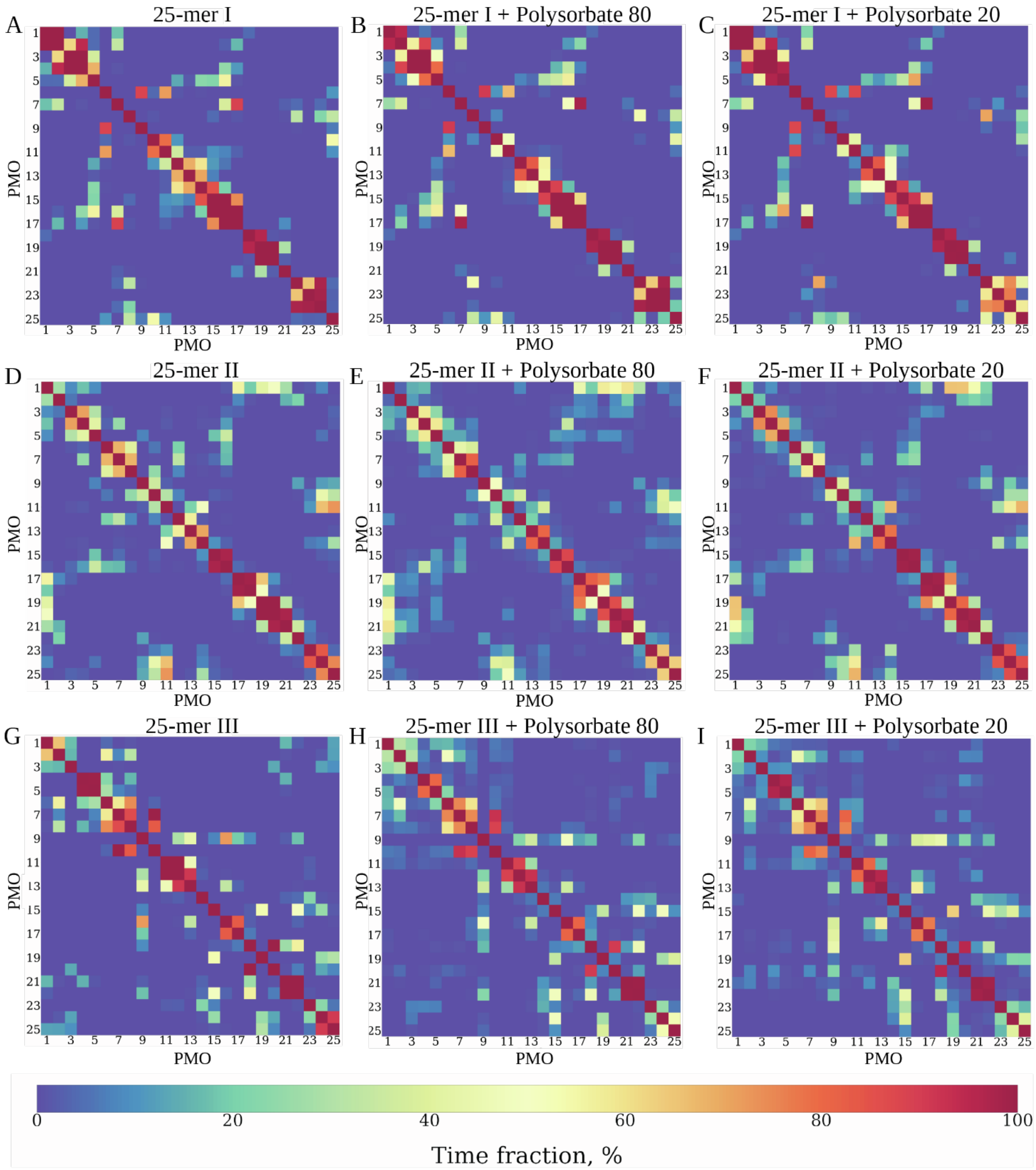

Figure S6. Intramolecular 25-mer-PMO interactions in the presence of Polysorbate 20 and Polysorbate 80. Displayed are maps showing the time fraction of intramolecular interactions between PMO nucleotides obtained from 1-μs MD simulations in the absence of surfactants for the 25-mer PMO conformer I (panel **A**), conformer II (panel **D**) and conformer III (panel **G**), in the presence of Polysorbate 80 for 25-mer PMO conformer I (panel **B**), conformer II (panel **E**) and conformer III (panel **H**), and in the presence of Polysorbate 20 for 25-mer PMO conformer I (panel **C**), conformer II (panel **F**) and conformer III (panel **I**). The numbering for PMO bases starts from the 3'-end of the PMO.

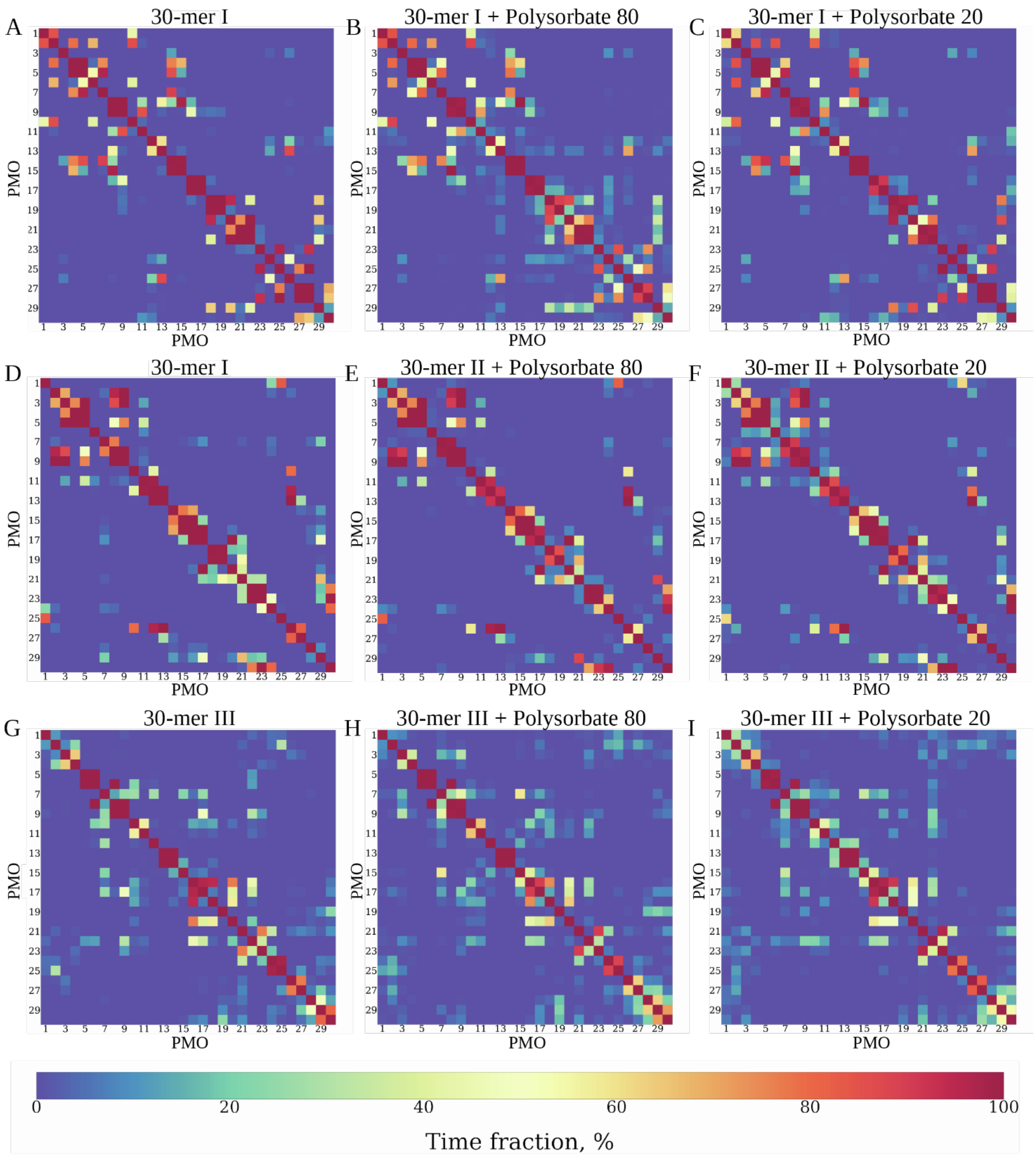

Figure S7. Intramolecular 30-mer-PMO interactions in the presence of Polysorbate 20 and Polysorbate 80. Displayed are maps showing the time fraction of intramolecular interactions between PMO nucleotides obtained from 1-μs MD simulations in the absence of surfactants for the 30-mer PMO conformer I (panel **A**), conformer II (panel **D**) and conformer III (panel **G**), in the presence of Polysorbate 80 for the 30-mer PMO conformer I (panel **B**), conformer II (panel **E**) and conformer III (panel **H**), and in the presence of Polysorbate 20 for the 30-mer PMO conformer I (panel **C**), conformer II (panel **F**) and conformer III (panel **I**). The numbering for PMO bases starts from the 3'-end of the PMO.
